# The causal interplay between depression and alcohol use from adolescence to young adulthood: a Mendelian randomization study

**DOI:** 10.1017/S0033291725102444

**Published:** 2026-01-27

**Authors:** Xuefei Wang, Shitong Xiang, Jujiao Kang, Rongquan Zhai, Chen Zheng, Tobias Banaschewski, Arun L.W. Bokde, Rüdiger Brühl, Sylvane Desrivières, Herta Flor, Hugh Garavan, Penny Gowland, Antoine Grigis, Andreas Heinz, Jean-Luc Martinot, Marie-Laure Paillère Martinot, Eric Artiges, Frauke Nees, Dimitri Papadopoulos Orfanos, Tomáš Paus, Luise Poustka, Michael N. Smolka, Sarah Hohmann, Nathalie Holz, Nilakshi Vaidya, Henrik Walter, Robert Whelan, Gunter Schumann, IMAGEN Consortium, Tianye Jia, Jianfeng Feng

**Affiliations:** 1 Institute of Science and Technology for Brain-Inspired Intelligence (ISTBI), Fudan University, Shanghai, China; 2 Key Laboratory of Computational Neuroscience and Brain-Inspired Intelligence, Fudan University, Ministry of Education, Shanghai, China; 3 Department of Child and Adolescent Psychiatry and Psychotherapy, Central Institute of Mental Health, Medical Faculty Mannheim, Heidelberg University, German Center for Mental Health (DZPG), partner site Mannheim-Heidelberg-Ulm, Mannheim, Germany; 4 Discipline of Psychiatry, School of Medicine and Trinity College Institute of Neuroscience, Trinity College Dublin, Dublin, Ireland; 5 Physikalisch-Technische Bundesanstalt (PTB), Braunschweig and Berlin, Germany; 6 Social, Genetic and Developmental Psychiatry Centre, Institute of Psychiatry, Psychology & Neuroscience, King’s College, London, UK; 7 Institute of Cognitive and Clinical Neuroscience, Central Institute of Mental Health, Medical Faculty Mannheim, Heidelberg University, Mannheim, Germany; 8 Department of Psychology, School of Social Sciences, University of Mannheim, Mannheim, Germany; 9 Departments of Psychiatry and Psychology, University of Vermont, Burlington, VT, USA; 10 Sir Peter Mansfield Imaging Centre School of Physics and Astronomy, University of Nottingham, University Park, Nottingham, UK; 11 NeuroSpin, CEA, Université Paris-Saclay, Gif-sur-Yvette, France; 12 Department of Psychiatry and Psychotherapy, University of Tübingen, Germany; German Center for Mental Health (DZPG), Site Tübingen, Germany; 13 Institut National de la Santé et de la Recherce Médicale, INSERM U A10 “Trajectoires développementales & psychiatrie”, University Paris-Saclay, Ecole Normale Supérieure Paris-Saclay, CNRS; Centre Borelli, Gif-sur-Yvette, France; 14 AP-HP. Sorbonne Université, Department of Child and Adolescent Psychiatry, Pitié-Salpêtrière Hospital, Paris, France; 15 Psychiatry Department, EPS Barthélémy Durand, Etampes, France; 16 Institute of Medical Psychology, Ludwig-Maximilians-Universität (LMU) in Munich, Munich, Germany; 17 Department of Psychiatry and Neuroscience, Faculty of Medicine and Centre Hospitalier Universitaire Sainte-Justine, University of Montreal, Montreal, QC, Canada; 18 Department of Psychiatry, McGill University, Montreal, Quebec, Canada; 19 Department of Child and Adolescent Psychiatry, Center for Psychosocial Medicine, University Hospital Heidelberg, Heidelberg, Germany; 20 Department of Psychiatry and Psychotherapy, Technische Universität Dresden, Dresden, Germany; 21 Centre for Population Neuroscience and Stratified Medicine (PONS), Department of Psychiatry and Psychotherapy, Charité Universitätsmedizin Berlin, Germany; 22 Department of Psychiatry and Psychotherapy CCM, Charité – Universitätsmedizin Berlin, corporate member of Freie Universität Berlin, Humboldt-Universität zu Berlin, and Berlin Institute of Health, Berlin, Germany; 23 School of Psychology and Global Brain Health Institute, Trinity College Dublin, Ireland; 24 Centre for Population Neuroscience and Precision Medicine (PONS), Institute for Science and Technology of Brain-inspired Intelligence (ISTBI), Fudan University, Shanghai, China; 25 German Center for Mental Health (DZPG), Site Berlin-Potsdam, Germany; 26 School of Psychology, Southampton University, Southampton, UK; 27 Department of Neurobiology, School of Basic Medical Sciences, National Institute on Drug Dependence, Peking University, Beijing, China; 28 Beijing Key Laboratory of Drug Dependence, Peking University, Beijing, China; 29 Zhangjiang Fudan International Innovation Center, Shanghai, China; 30 School of Mathematical Sciences and Centre for Computational Systems Biology, Fudan University, Shanghai, China; 31 Department of Computer Science, University of Warwick, Coventry, UK; 32 Fudan ISTBI–ZJNU Algorithm Centre for Brain-inspired Intelligence, Zhejiang Normal University, Jinhua, China

**Keywords:** adolescence, alcohol use disorder, causal interplay, cross-lagged panel model, genetic instrumental variables, longitudinal study, mediation analysis, Mendelian randomization, mood disorder, polygenic risk scores, self-medication, sex differences

## Abstract

**Background:**

Depression is often comorbid with alcohol use problems, and sex differences may further complicate this interplay.

**Methods:**

We conducted a longitudinal study using a large European adolescent cohort assessed at ages 14 (baseline, BL), 16 (follow-up 1, FU1), 19 (follow-up 2, FU2), and 23 (follow-up 3, FU3). Depression and alcohol use were measured using standardized behavioral scales. Cross-lagged analysis, improved Mendelian randomization (MR) analysis, and mediation analysis were conducted to infer the causal interplay.

**Results:**

2110 adolescents were included at baseline (49% male). Depression and alcohol consumption demonstrated a significant positive correlation (*r_BL_* = 0.094, *p_BL_* = 1.58E-05, 95% CI = [0.052, 0.137]), which gradually diminished over time and eventually became significantly negative. Depression and alcohol use problems remained strongly correlated across three timepoints (*r* > 0.074, *p* < 6.76E-03). Cross-lagged analysis suggested that depression predicted future alcohol use problems: *β_BL-FU1_* = 0.058, *p* = 0.021, 95% CI = [0.009, 0.108]; *β_FU2-FU3_* = 0.142, *p* = 8.34E-07, 95% CI = [0.113, 0.263]. MR analyses confirmed this causal interplay (*r_mean_* = 0.043, longitudinal *p_permuation_* < 0.001). Interestingly, MR analyses also indicated that alcohol consumption might alleviate depression (*r_mean_* = −0.022, longitudinal *p_permutation_* = 0.043), particularly in females at FU3, of which the anxiety status and the personality trait neuroticism largely mediated the effect. These findings were validated in an independent matched sample (N = 562) from Human Connectome Project.

**Conclusions:**

Depression may predict future alcohol use problems, whereas moderate alcohol consumption might alleviate depressive symptoms, especially in females.

## Introduction

Depression stands as one of the most prevalent psychiatric conditions, characterized by high incidence and recurrence rates, and it has caused a massive social burden worldwide (Mathers & Loncar, 2006). Extensive epidemiological investigations have indicated frequent comorbidity between depression and other mental illnesses (Stover, Fenton, Rosenfeld, & Insel, 2003). Most notably, alcohol use disorder (AUD) and depression often coexist, with a comorbidity prevalence exceeding 40% (Riper et al., 2014). However, their exact causal relationships are highly complicated, with inconsistent findings in the literature.

Evidence has demonstrated that depressive symptoms precede increased alcohol consumption in adolescents (Edwards et al., 2014) and may underlie dependence symptoms and relapse (Vieten, Astin, Buscemi, & Galloway, 2010). Conversely, light or moderate alcohol use may have a beneficial effect on depression (Baum-Baicker, 1985). For instance, a prospective study by Kim et al. found that moderate alcohol consumption might be associated with a reduced risk of developing depression (Kim, Kim, Oh, & Lee, 2024). Further, there is also literature proposing that alcohol may be generally used to cope with depressive symptoms to temporarily avoid psychological stress, even without a clear therapeutic effect (Boden & Fergusson, 2011). Therefore, it has been proposed that individuals with depression often use alcohol to alleviate negative emotions related to depression or anxiety, that is, as a negative reinforcer, which, however, ends up with maladaptive consumption patterns and an increased risk for comorbidities, as suggested by the self-medication hypothesis (SMH) (Turner, Mota, Bolton, & Sareen, 2018). Despite lacking direct evidence in humans, the putative causal loop motivating self-medication behavior, that is depression leads to raised alcohol use behavior, and light to moderate alcohol use may help to alleviate depression, has been confirmed in animal models (Jain, Kannamwar, & Verma, 2017; Zhao et al., 2020). Interestingly, a recent large population cohort study found no associations between the frequency and quantity of alcohol use and depression in adults (Hammerton et al., 2023). However, this surprising lack of association could be explained by the coexistence of both positive and negative latent associations, where the latent positive associations aligned with the highly comorbid (likely mutual strengthening) depression and alcohol abuse, and the negative association could result from the potential alleviation effect (even temporally) of alcohol use on depression, which together correspond well with the previously mentioned potential mechanism of self-medication behavior. Further, sex differences may also play an important role in this complexity, as evidence suggests that men are more likely to have alcohol use problems (Hasin, Stinson, Ogburn, & Grant, 2007), while women are more likely to self-medicate for depressive symptoms (Robinson, Ingram, & Degan, 2024).

Regrettably, few random trials are available to confirm the causal loop between depression and alcohol use in humans (Nunes, 2023). Nevertheless, previous studies have indicated shared, albeit incongruent, genetic architectures between alcohol use behavior and depression: the quantity of alcohol consumption exhibited a positive genetic correlation with both alcohol dependence and major depression, while the frequency of alcohol consumption demonstrated a negative genetic correlation with major depression and showed no significant genetic correlation with alcohol dependence (Polimanti et al., 2019). While the above genetic findings might imply complicated causal relationships between alcohol use and depression, the effect of pleiotropy or the potential impact of mutual causal influence cannot be ruled out.

As an alternative, Mendelian randomization (MR) is a powerful statistical strategy used to investigate causal relationships between two genetically determined phenotypes in observational data, by employing genetic variations as instrumental variables to form a naturally established random trial (Bowden & Holmes, 2019). MR has previously been used to study the causal relationship between alcohol use and depression (Treur et al., 2021). However, traditional MR analyses are entirely based on summary statistics of genome-wide association study (GWAS) from third-party datasets, which typically do not match characteristic features such as sex or age in different samples. Therefore, the resulting causal inference (whether significant or not) may not be applicable to all datasets if the underlying genetic architectures are sex- or age-dependent, which is likely the case for both alcohol use behavior and depression (Schuler, Vasilenko, & Lanza, 2015). The research team recently proposed an improved MR approach by employing nonpleiotropic polygenic risk scores (i.e. the valid-PRS) as the instrumental variables (Kang et al., 2022), which not only could mitigate the impact of horizontal pleiotropy but also allow us to derive robust and detailed conclusions on the causal loop between depression and alcohol use in any given datasets.

In summary, while a plethora of literature has indicated a potential causal association between depression and alcohol use, there is currently no consensus about their exact causal relationship. This study employs a cross-lagged panel model (CLPM) with longitudinal data and improved MR methods to closely investigate the potential causal effects between depression and alcohol use. The CLPM analyses multiwave longitudinal data to identify temporal associations between variables, though its causal inferences may be limited by unmeasured confounders. In contrast, MR utilizes genetic variants as instrumental variables to circumvent environmental confounding and provide genetic-level causal evidence, which, however, does not capture dynamic developmental processes. The integration of these methods not only validates the temporal nature of observed associations but also strengthens the robustness of causal inference. Divergent findings between methods may indicate time-sensitive or mechanism-specific effects (e.g. gene–environment interactions).

Furthermore, to uncover the psychosocial mechanisms underlying these associations, we will examine the mediating role of potential factors (e.g. the risk of personality for substance abuse, anxiety screening scores, and personality traits). Mediation analysis aims to elucidate the underlying mechanisms through which an independent variable (X) influences a dependent variable (Y) by identifying an intermediate variable (M) that explains the pathway process of ‘how X affects Y’ (Tönnies, Schlesinger, Lang, & Kuss, 2023). Notably, previous studies have indicated that impulsivity plays a mechanistic role in the relationship between depression and alcohol use problems among adolescents (Pang et al., 2014) and young adults (Gonzalez, Reynolds, & Skewes, 2011). Moreover, given the high comorbidity between depression and anxiety (Kalin, 2020), SMH is operationally defined as the self-reported use of alcohol or substances to cope with depressive or anxious moods (Turner, Mota, Bolton, & Sareen, 2018). Finally, as mental disorders are closely related to neuroticism, a characteristic personality that is vulnerable to emotional problems like depression and anxiety disorders (Lahey, 2009), it will be interesting to investigate whether alcohol consumption may alleviate depression by suppressing neurotic behaviors. Specifically, we hypothesize that: 1) depression indirectly leads to alcohol use problems, potentially through impulsive use, such as negative urgency; and 2) alcohol consumption alleviates subsequent depressive symptoms by reducing internal symptoms such as anxiety and neuroticism. This multimethod framework tests whether a causal relationship exists and clarifies how the association arises. Our findings may offer new therapeutic indications for clinical practice.

## Methods

### Participants

This study utilized data from the IMAGEN project, a prospective, multicenter, longitudinal imaging genetics study that recruited over 2,000 adolescents (Schumann et al., 2010). Detailed information about the IMAGEN dataset, including ethics, recruitment, and neuropsychological assessments, is available at http://imagen-project.org. For validation, we included data from the Human Connectome Project (HCP), a large-scale shared neuroscience research dataset accessible at https://www.humanconnectome.org. A subset of 562 white participants (262 females) aged 22 to 30 years was selected for validation analyses. The project was approved by local ethics research committees, and informed consent was obtained from all participants or parents/guardians of adolescent participants (Van Essen et al., 2012).

### Measurement of behavior

In the IMAGEN cohort, depressive symptoms were measured across all timepoints by combining the Development and Well-Being Assessment (DAWBA; 6 items) and the Strengths and Difficulties Questionnaire (SDQ; 2 items) (see Supplementary Method S1 and Supplementary Table S1) as suggested in a previous study by Xie et al., 2021. Alcohol use behavior was assessed using screening questions from the Alcohol Use Disorders Identification Test (AUDIT). Specifically, we defined two correlated but distinct key measures: alcohol consumption (three items) and alcohol use problems (seven items) (see Supplementary Method S2 and Supplementary Table S2). To assess the risk of personality for substance abuse, we employed the Substance Use Risk Profile Scale (SURPS) (see Supplementary Table S3). Additionally, anxiety symptoms were evaluated using the Anxiety Screening Scale (ANXDX) from the Composite Diagnostic Interview (DIA-X/M-CIDI) (see Supplementary Table S4) for mediation analyses. Covariates such as sex and research sites were regressed out in subsequent analyses. This approach is consistent with that used in previous IMAGEN studies (Xiang et al., 2023). A sensitivity analysis was conducted, controlling for additional variables, including externalizing symptoms, socioeconomic status, and personality traits (Costa & McCrae, 1992) (see Supplementary Method S3 and Supplementary Table S5).

For the HCP cohort, depression scores were assessed using the ASR DSM Depressive Problems scale, adjusted for age and sex. We also examined the number of endorsed depressive symptoms that meet DSM-IV criteria for major depression over an individual’s lifetime. Alcohol use problems were evaluated based on the sum of DSM-IV criteria for alcohol dependence and alcohol abuse, while alcohol consumption was measured as drinks consumed per drinking day in the past 12 months. Sensitivity analyses were conducted by additionally adjusting for the top 10 genetic principal components (PCs), along with age and sex. Notably, this is different from the procedure for the IMAGEN data, where the top 10 PCs were not included as covariates, as they are redundant to research sites that not only captured the majority (87–88%) of the variance explained by these PCs (Supplementary Figure S1) but also controlled for nongenetic site effects.

### Cross-lagged analysis

We examined the longitudinal relationship between alcohol consumption, alcohol use problems, and depressive symptoms using a CLPM implemented in the ‘lavaan’ package (version 0.6) in R (version 4.2.2) (Rosseel, 2012). To ensure robustness, all covariates were regressed out before analysis, and the residuals were used in the model. Maximum likelihood estimation was employed to estimate model parameters. Standardized estimates are reported with their 95% confidence intervals and the corresponding two-tailed *p*-values. Given the significant sample attrition across waves, primary analyses were conducted using pairwise CLPMs between consecutive time points. This approach helped maximize statistical power and reduce potential bias due to missing data. In addition, a random-intercept cross-lagged panel model (RI-CLPM) incorporating all four time points was estimated to validate the findings (Madigan et al., 2019).

### Improved Mendelian randomization approach

The GWAS data of daily alcohol use (continuous, log transformed grams per day) was obtained from Schumann et al., 2016 based on a pooled sample (N = 74,711). The major depressive disorder (MDD) GWAS data was sourced from a meta-analysis by Wray NR et al. with 135458 cases and 344901 controls (Wray et al., 2018). To further validate the potential causal effects suggested by the cross-lagged analysis, we applied an improved MR analysis (Kang et al., 2022), which utilizes nonpleiotropy PRSs as instruments to allow robust causal inference between the paired variables of interest. We systematically removed pleiotropic single nucleotide polymorphisms (SNPs) associated with both the exposure and outcome, retaining only SNPs exclusively linked to the exposure, which were then used to compute PRS as effective instrumental variables (valid-PRS). Particularly, we followed the PRS processing methodology described by Xiang et al. (2023) and computed valid-PRS using PLINK software (Purcell et al., 2007). The valid-PRS were denoted as A/B c PRS (where c ranged from 0.05 to 0.5 in increments of 0.05). Here, A represents the PRS for genetic variants related to A (*p* < 0.05) but not to B (*p* > c). To evaluate the longitudinal significance, we established a union of all participant IDs across the four timepoints. The union IDs were then randomly reshuffled 1,000 times to generate the permuted phenotypes at each timepoint, maintaining consistent new ID orders. At each iteration, 40 *r*-values were computed between permuted phenotypes and valid-PRSs across 10 thresholds and 4 timepoints. The null distribution of the mean (*r_mean_*) of these 40 *r*-values could hence be established, as well as the corresponding *p*-value of the observed *r_mean_* of 40 *r*-values across 10 thresholds and 4 timepoints. A longitudinal *p* < 0.05 was considered overall significant, which also alleviates the need for multiple corrections when assessing significance at each timepoint (Chen et al., 2023). Notably, the valid-PRS should have a significant longitudinal correlation with the corresponding phenotypes to be validated as an instrumental variable prior to a meaningful causal inference, which is an essential analogue to the two-stage regression commonly adopted for causal inferences (Angrist & Imbens, 1995).

In the present study, the mutual causal relationship between depression and alcohol was examined through the association of valid-PRS_MDD_ with alcohol use problems and the association of valid-PRS_alcohol_ with depression. We also calculated the mean of 10 *r*-values and its *p*-value at each timepoint with a 1,000 permutation to investigate the corresponding validity of the instrumental variable and the significance of causal inference. A sensitivity analysis was conducted, excluding these participants who never initiated alcohol use across all waves.

### Mediation analysis

The mediation analysis was conducted using the approach proposed by Baron and Kenny (1986). Specifically, we assessed the significance of a potential mediation pathway by employing a hypothetic test using 10,000 times bootstrapped samples to obtain the empirical distribution of the mediation effects at the alternative hypothesis and calculated the corresponding *p*-value. Furthermore, we conducted a serial mediation analysis using the ‘lavaan’ package to examine whether the association between valid-PRS_MDD_ and FU1 alcohol use problems was mediated through hopelessness and impulsivity. Based on theoretical expectations and prior evidence supporting the hypothesized direction of the mediation effect, all *p*-values and confidence intervals are reported as one-tailed.

## Results

### Demographics of participants

This cohort study analysed participants enrolled in the IMAGEN project. Adolescents were recruited at age 14 from schools across eight sites in Germany (Mannheim, Dresden, Berlin, and Hamburg), the United Kingdom (London and Nottingham), Ireland (Dublin), and France (Paris). Self-reported alcohol consumption and depression scores were assessed at ages 14 (baseline), 16 (follow-up 1, FU1), 19 (follow-up 2, FU2), and 23 (follow-up 3, FU3), as shown in Table 1. The results for males and females are shown in Supplementary Table S6. Depression scores were significantly higher in females than in males at all four timepoints. Alcohol consumption was significantly higher in males than in females after age 16 (*p* < 2.65E-11). Alcohol use problems were significantly higher in males than in females after age 19 (*p* < 0.01).

**Table 1.** Characteristics of the study population in the IMAGEN cohort

Variable	Baseline		Follow-up 1		Follow-up 2		Follow-up 3	
Age	14		16		19		23	
Questionnaire	Number	Male (n, %)	Number	Male (n, %)	Number	Male (n, %)	Number	Male (n, %)
DAWBA and SDQ	2109	1034 (49.03)	1660	800 (48.19)	1352	627 (46.38)	1283	596 (46.45)
AUDIT	2110	1034 (49.00)	1672	804 (48.09)	1488	703 (47.24)	1337	622 (46.52)

Quantitative measurements	Mean	SD	Mean	SD	Mean	SD	Mean	SD
Depression scores	2.04	1.86	2.24	2.11	1.73^a^	1.93	1.59^a^	1.87
Alcohol consumption	1.07	1.51	2.86	2.32	4.23	2.54	4.14	2.25
Alcohol use problems	0.46	1.58	1.04	2.05	1.65	2.80	1.94	2.99

*Note:* DAWBA: Development and Well-Being Assessment; SDQ: Strengths and Difficulties Questionnaire; AUDIT: Alcohol Use Disorders Identification Test.

a Depression scores were calculated as the sum of 8 items in baseline and follow-up 1. However, with ‘irritable’ missing for both follow-ups 2 and 3, the corresponding depression scores were computed as the sum of the rest 7 items.

To assess sample retention and missingness across waves, we compared participants who remained in the study with those who dropped out at each follow-up (FU1, FU2, FU3), based on baseline variables including depression scores, alcohol consumption, alcohol use problems, and sex (Supplementary Table S7). As for depression scores, attrition rates increased over time, from 21.4% at FU1 to 39.4% at FU3. There were no statistically significant differences in baseline depression scores between retained and dropped participants at any wave (all *p* > 0.05). However, chi-square tests revealed significant sex differences in attrition at FU2 (χ^2^ = 9.734, *p* = 0.0018) and FU3 (χ^2^ = 8.958, *p* = 0.0028), with males being more likely to drop out. Similarly, attrition rates based on AUDIT measures rose from 21.1% at FU1 to 37.1% at FU3. Participants who dropped out had significantly higher baseline levels of alcohol consumption and alcohol use problems across all waves (all *p* < 0.002). Sex was also significantly associated with attrition at FU2 (χ^2^ = 5.784, *p* = 0.0168) and FU3 (χ^2^ = 8.473, *p* = 0.0036), with higher dropout among males.

The prevalence of any alcohol use (i.e. alcohol consumption or alcohol use problems) was 51.6% at baseline, 82.8% at FU1, 92.9% at FU2, and 94.9% at FU3. No participants met the criteria for lifetime abstainers, defined as individuals with a total alcohol use score (the sum of alcohol consumption and alcohol use problems) > 7 at baseline but with no alcohol use from FU1 to FU3. There were 19 individuals (1.75% of the 1087 participants with data available at all four timepoints) who never initiated alcohol use across all waves. Given the small number of such cases, they were not treated separately in the main analyses. To ensure the robustness of our findings, we conducted sensitivity analyses excluding these participants who never initiated alcohol use across all waves in MR analysis (see Supplementary Table S12), which showed consistent results.

### Behavioral correlation

**Figure 1.**
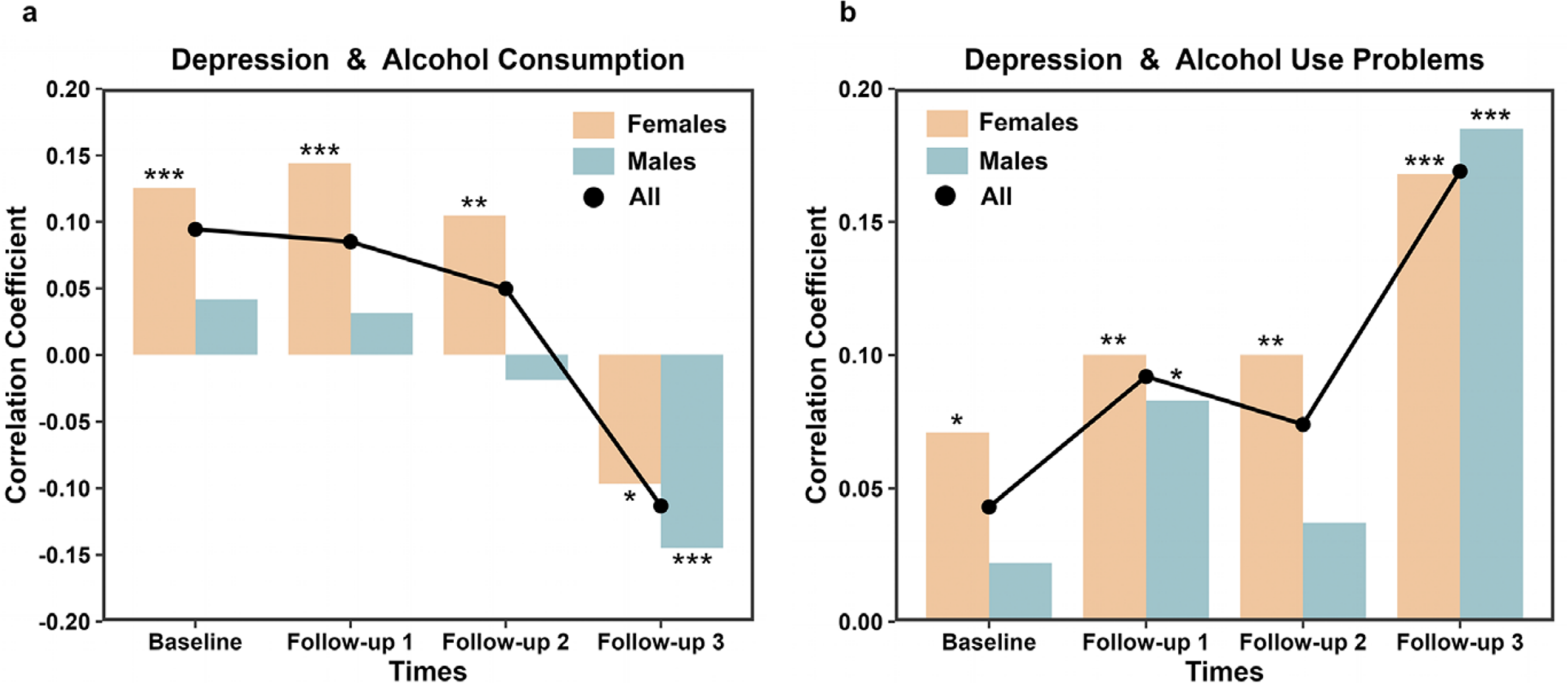
(a) Correlations between depression and alcohol consumption at four timepoints (controlled for alcohol use problems); (b) Correlations between depression and alcohol use problems at four timepoints (controlled for alcohol consumption). *p < 0.05, **p < 0.01, ***p < 0.001.

Depression scores and alcohol consumption showed a significant positive correlation (controlled for alcohol use problems) at baseline (*r_BL_* = 0.094, *p_BL_* = 1.58E-05, 95% CI = [0.052, 0.137]), which gradually diminished over time and eventually became negative (*r_FU1_* = 0.085, *p_FU1_* = 6.08E-04, 95% CI = [0.037, 0.133]; *r_FU2_* = 0.050, *p_FU2_* = 0.070, 95% CI = [−0.004, 0.103]; *r_FU3_* = −0.113, *p_FU3_* = 5.35E-05, 95% CI = [−0.167, −0.059]). This trend was observed in both males and females (Figure 1). In contrast, the correlation between depression scores and alcohol use problems (controlled for alcohol consumption) remained consistently positive across all timepoints (*r_BL_* = 0.043*, p_BL_* = 0.051, 95% CI = [0, 0.086]; *r_FU1_* = 0.092, *p_FU1_* = 2.07E-04, 95% CI = [0.044, 0.140]; *r_FU2_* = 0.074, *p_FU2_* = 6.76E-03, 95% CI = [0.021, 0.128]; *r_FU3_* = 0.169, *p_FU3_* = 1.57E-09, 95% CI = [0.115, 0.222]). These findings suggest a persistent causal influence of depression on increased alcohol use problems, an effect that was primarily observed in females, with males being less affected (Figure 1 and Supplementary Table S8). To further validate these associations, we conducted additional analyses controlling for externalizing symptoms, socioeconomic status, and personality traits. While most additional control variables had minimal impact, personality traits significantly influenced the observed relationships (Supplementary Table S8). Specifically, after adjusting for both alcohol use problems and personality traits, the negative correlation between depression scores and alcohol consumption at FU3 became nonsignificant (*r_FU3_* = −0.003, *p_FU3_* = 0.904, 95% CI = [−0.059, 0.052]). Similarly, the persistent positive correlations between depression scores and alcohol use problems (controlled for alcohol consumption and personality traits) became nonsignificant at all timepoints (|*r|* ≤ 0.048, *p* ≥ 0.088, Supplementary Table S8). Subsequent analysis identified the personality trait neuroticism as the primary factor driving these effects (Supplementary Table S8). Mediation analysis confirmed that neuroticism significantly mediated the relationship between alcohol use problems and depression at FU3 (*β_mediation_* = 0.067, mediation proportion 65.2%, *p_bootstrap_* < 0.0001, 95% CI = [0.044, ∞]) (Supplementary Table S9).

### Cross-lagged analysis findings

**Figure 2.**
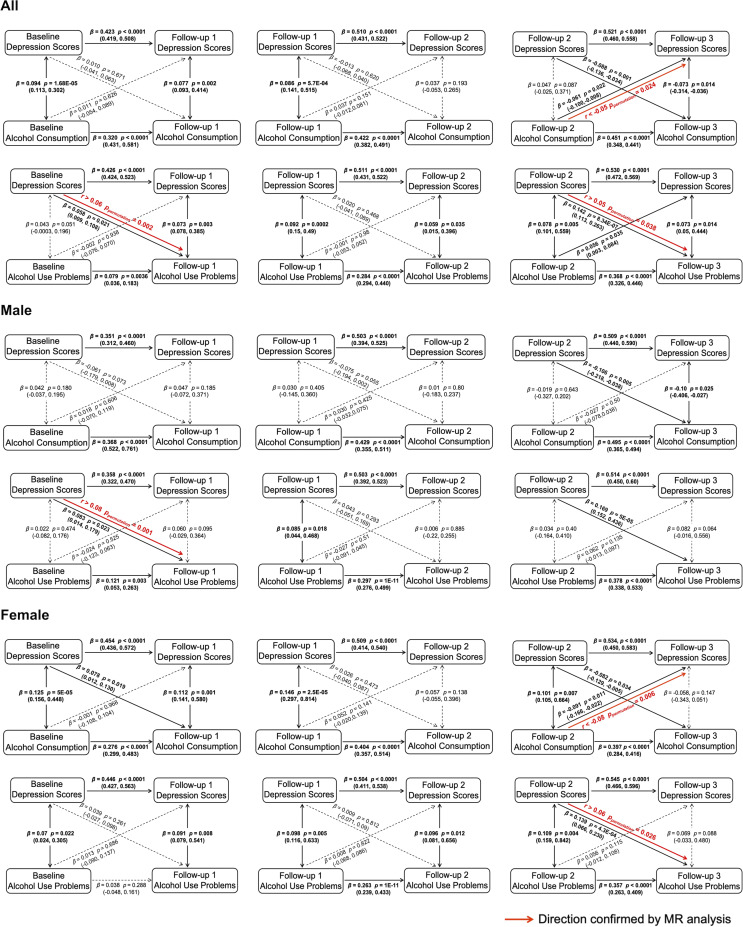
The longitudinal associations between depression scores and alcohol consumption/alcohol use problems were investigated by CLPM (controlled for the other alcohol use behaviour). Solid lines represent significant associations (two-tailed *p* < .05), while dashed lines represent nonsignificant associations (two-tailed *p* > .05). Causal inferences (highlighted in red) were confirmed with improved Mendelian randomization (MR). *r* is the correlation between the valid-PRS and the corresponding behaviour, and *p_permutation_* is the value of the permutation test (see Supplementary Table S11).

To verify the possible causal relationship underlying the self-medication behavior, we employed the CLPM (see Supplementary Table S10 for the model fit measures) to investigate the longitudinal predictive effects between depression and alcohol use across the four timepoints. We found significant positive predictive effects of depression on later alcohol use problems: *β_BL-FU1_* = 0.058, *p* = 0.021, 95% CI = [0.009, 0.108]; *β_FU2-FU3_* = 0.142, *p* = 8.34E-07, 95% CI = [0.113, 0.263]; Male: *β_BL-FU1_* = 0.083, *p* = 0.023, 95% CI = [0.014, 0.179]; *β_FU2-FU3_* = 0.169, *p* = 5E-05, 95% CI = [0.152, 0.436]; Female: *β_FU2-FU3_* = 0.139, *p* = 4.3E-04, 95% CI = [0.066, 0.230] (Figure 2), indicating a potential causal effect of depression on increased alcohol use problems over time. Conversely, we observed significant positive predictive effects of alcohol use problems on later depression, though only at FU2-FU3: *β_FU2-FU3_* = 0.056, *p* = 0.035, 95% CI = [0.003, 0.084] (Figure 2). Interestingly, alcohol consumption was positively associated with subsequent depression in early stages but negatively associated in later stages. This negative association in later stages was significant (*β_FU2-FU3_* = −0.061, *p* = 0.022, 95% CI = [−0.100, −0.008]; Female: *β_FU2-FU3_* = −0.091, *p* = 0.011, 95% CI = [−0.166, −0.022]; Figure 2). These results may indicate the coexistence of different latent effects underlying the relationship between alcohol consumption and depression. These findings were largely consistent in the RI-CLPM, as summarized in Supplementary Figures S2 and S3.

### Improved Mendelian randomization analyses between depression and alcohol use

Our improved MR approach firstly systematically removed all possible pleiotropic SNPs from the PRSs (estimated using PLINK) (Purcell et al., 2007) at various thresholds up to a 50% chance, that is lower than a random association with the outcome phenotype (see Methods for more details), to generate the nonpleiotropic PRS. We then validated the nonpleiotropic PRSs (i.e. the valid-PRSs) as instrumental variables by confirming their associations with the corresponding exposure phenotypes across all the timepoints (depression: *r_mean_* = 0.065, longitudinal *p_permutation_* < 0.001; alcohol consumption: *r_mean_* = 0.067, longitudinal *p_permutation_* < 0.001) in the IMAGEN sample (Supplementary Table S11). The established valid-PRSs, as instrumental variables, by definition, should only have direct contributions to the exposure phenotype, and hence the valid-PRSs’ further associations with the outcome phenotype would indicate a potential causal effect from the exposure to the outcome (the above approach is analogue to the two-stage regression commonly adopted for causal inferences) (Angrist & Imbens, 1995). Across the four timepoints, we found that the valid-PRS_MDD_ was significantly associated with alcohol use problems (*r_mean_* = 0.043, longitudinal *p_permutation_* < 0.001). On the other hand, the valid-PRS_alcohol_ showed significant negative correlations with depression scores across the four timepoints (*r_mean_* = −0.022, longitudinal *p_permutation_* = 0.043). Furthermore, we observed sex differences in the causal inferences (Supplementary Table S11). In males, valid-PRS_MDD_ significantly increased alcohol use problems across all four timepoints (*r_mean_* = 0.056, longitudinal *p_permutation_* < 0.001), whereas valid-PRS_alcohol_ had a negative but nonsignificant effect on depression scores (*r_mean_* = −0.005, longitudinal *p_permutation_* = 0.385). In females, valid-PRS_MDD_ showed positive but nonsignificant associations with alcohol use problems across all four timepoints (*r_mean_* = 0.027, longitudinal *p_permutation_* = 0.058), and a significantly positive association specifically at FU3 (*r* > 0.06, *p_permutation_* = 0.026). Conversely, valid-PRS_alcohol_ significantly reduced depression scores across all four timepoints (*r_mean_* = −0.034, longitudinal *p_permutation_* = 0.027), with a particularly strong effect at FU3 (*r* < −0.08, *p_permutation_* = 0.006). These findings support the causal loop between depression and alcohol use that may underlie self-medication behavior. In sensitivity analyses, MR results remained consistent after excluding 19 individuals who never initiated alcohol use across all four waves (Supplementary Table S12).

To validate the causal inferences identified above, we further investigated the 562 white participants aged 22 to 30 from the HCP cohort. Consistent with the initial findings in IMAGEN, the HCP cohort showed that depression scores were significantly correlated with alcohol use problems (*r* = 0.207, *p* = 8.28E-07, 95% CI = [0.127, 0.293]) but not with alcohol consumption (*r* = −0.061, *p* = 0.160, 95% CI = [−0.144, 0.022]). This association became significantly negative when controlling for alcohol use problems (*r* = −0.131, *p* = 0.002, 95% CI = [−0.215, −0.049]; test for difference *t* = 5.56). The correlation between depression and alcohol use problems remained significant even after controlling for alcohol consumption (alcohol use problems: *r* = 0.235, *p* = 3.40E-08, 95% CI = [0.157, 0.323]) (Supplementary Table S13). Like in the IMAGEN cohort, valid-PRS_MDD_ could significantly increase alcohol use problems (*r* > 0.10, *p_permutation_* = 0.041), while valid-PRS_alcohol_ could significantly reduce the risk for depression (ASR DSM) only in females (*r* < −0.15, *p_permutation_* = 0.047), and valid-PRS_alcohol_ exhibited a more significant reducing effect on the indicator that reflects the number of endorsed depressive symptoms meeting DSM-IV criteria for major depression over an individual’s lifetime among females (*r* < −0.20, *p_permutation_* = 0.002) (Supplementary Table S14). We also conducted sensitivity analyses. Besides controlling for age and sex, we included the top 10 PCs as covariates, and the results remained largely unchanged (Supplementary Table S15).

Altogether, based on the improved MR, we affirm the following conclusion: depression may lead to increased problematic alcohol use in adolescents or young adults, while light or moderate alcohol intake may alleviate depressive symptoms, particularly among young female adults, thus supporting the SMH.

### Mediation analysis

**Figure 3.**
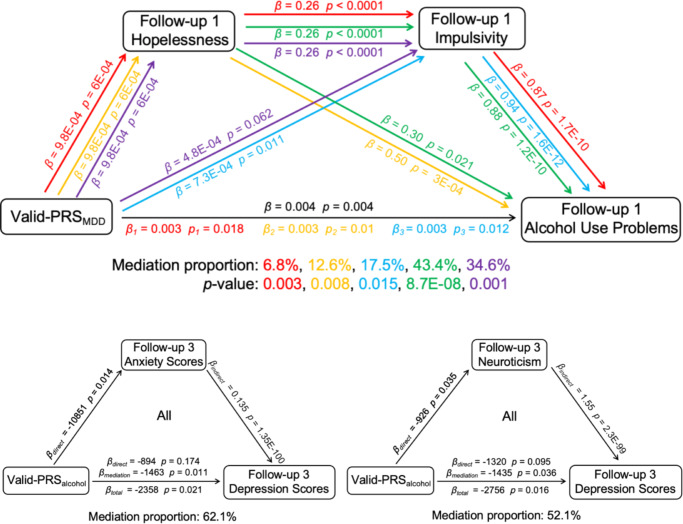
The results of mediation analysis. The *p*-values of the mediation effects were obtained with 10000 times bootstrap samplings. All *p*-values are reported as one-tailed.

We next investigated potential mediation effects in the above causal effects. At FU1, both hopelessness and impulsivity mediated the positive associations between valid-PRS_MDD_ (SNPs with a *p*-value greater than 0.05 for alcohol consumption) and alcohol use problems (hopelessness: *β_mediation_* = 5E-04, mediation proportion 12.6%, *p_bootstrap_* = 0.0007, 95% CI = [1.65E-04, ∞]; impulsivity: *β_mediation_* = 6.87E-04, mediation proportion 17.5%, *p_bootstrap_* = 0.011, 95% CI = [1.6E-04, ∞]). Further, a serial mediation involving both hopelessness and impulsivity accounted for 6.8% (*β_mediation_* = 2.2E-04, *p_bootstrap_* = 0.003, mediation proportion 6.8%, 95% CI = [8.6E-05, ∞]) of the association between valid-PRS_MDD_ and alcohol use problems (Figure 3). At FU3, only hopelessness mediated the positive associations between valid-PRS_MDD_ and alcohol use problems (*β_mediation_* = 9.2E-04, mediation proportion 23.7%, *p_bootstrap_* = 0.0009, 95% CI = [2.7E-04, ∞]; females: *β_mediation_* = 0.0013, mediation proportion 21.2%, *p_bootstrap_* = 0.0035, 95% CI = [2.6E-04, ∞]). Anxiety scores (sum of ANXDX 12 items) mediated the negative association between valid-PRS_alcohol_ (SNPs with a *p*-value greater than 0.05 for depression) and depression scores at FU3 (*β_mediation_* = −1463, mediation proportion 62.1%, *p_bootstrap_* = 0.011, 95% CI = [−∞, −413]), an effect was primarily driven by females (*β_mediation_* = −3023, mediation proportion 68.3%, *p_bootstrap_* = 0.0015, 95% CI = [−∞, −1237]). We also found that neuroticism significantly mediated the relationship between valid-PRS_alcohol_ and depression at FU3 (all: *β_mediation_* = −1435, mediation proportion 52.1%, *p_bootstrap_* = 0.036, 95% CI = [−∞, −103]; females: *β_mediation_* = −2501, mediation proportion 52.6%, *p_bootstrap_* = 0.02, 95% CI = [−∞, −477]). The above findings were summarized in Supplementary Table S16.

## Discussion

In this study, we utilized a large longitudinal dataset of adolescents and employed various analytical methods to comprehensively investigate the causal relationships between alcohol use behaviors and depression from adolescence to early adulthood. We found that the association between depression and alcohol consumption gradually diminished over time and eventually became negative, while the association between depression and alcohol use problems remained highly consistent and positive. With an improved MR approach, which minimized confounding effects such as pleiotropy and reverse inference, we demonstrated the existence of a causal interplay in both the IMAGEN and HCP cohorts, where depression could increase alcohol use problems, a potential aftermath of attempted self-medication with alcohol, and alcohol consumption may indeed alleviate depressive symptoms, that is a reinforcer of using alcohol for self-medication, particularly in young female adults. Together, this causal interplay may help to explain the maladaptive self-medication behavior, also known as the SMH.

Human research, unlike animal experiments, does not allow for in vivo experiments, and the causal relationship between alcohol and depression has not been conclusively demonstrated in previous studies. Establishing the definite causal relationships between depression and alcohol poses a significant challenge for scientists. Genetic correlation analysis revealed a nonsignificant negative relationship between depression and alcohol consumption (*r* = −0.003, *p* = 0.963, using LDSC software at https://github.com/bulik/ldsc), (Bulik-Sullivan et al., 2015) with the top overlapped SNP for both disorders displaying inverse associations (Supplementary Table S17), an indication for potential pleiotropic effects or complex causal interplay. In such a circumstance, it is no wonder that traditional two-sample MR approaches have failed to reach a conclusion on the causality relationships between depression and alcohol use based on some of the largest GWAS findings (Supplementary Figure S4). Therefore, we further investigated the causal relationship between depression and alcohol through cross-lagged analysis and an improved MR approach, the latter effectively controlling for confounding factors such as pleiotropy and reverse causal effect, hence reinforcing the robustness of our findings. Our study revealed the causal interplay among alcohol consumption, depression and alcohol use problems that the maladaptive attempt to use alcohol to alleviate anxiety and depressive symptoms may end up with increased alcohol use problems, as suggested by the SMH. This conclusion was also corroborated in the HCP cohort. Notably, our research also unveiled sex differences, with only females demonstrating beneficial alcohol consumption for depression alleviation in early adulthood.

Various theories have been proposed for a causal relationship between alcohol use and depression but none provide definitive evidence in humans (Boden & Fergusson, 2011). Notably, both previous and present studies have found a nonsignificant association between depression and alcohol consumption in early adulthood (Taylor & Rehm, 2005). Instead, a strong correlation between depression and alcohol use problems has been consistently observed (Hammerton et al., 2023). An intuitive interpretation could be that, since there is no association between alcohol consumption and depression, the strong association between depression and alcohol use problems must stem from alcohol use problems, that is alcohol use problems causally lead to depression. However, strong evidence also suggests a more complex dynamic (Hussong et al., 2011). For instance, in the present study, the negative association between depression and alcohol consumption in early adulthood was preceded by a gradually diminished positive correlation, whereas the correlation between depression and alcohol use problems (which also implies more intensive alcohol use) remained high. Thus, it is likely that the null association between depression and alcohol consumption during early adulthood was not due to independence but rather the result of two opposing effects cancelling each other out. Further, in the sensitivity analyses, we found that adding externalizing symptoms and socioeconomic status as control variables did not significantly alter our primary findings. However, neuroticism, a personality trait characterized by heightened emotional reactivity and a predisposition to negative affect (Kotov, Gamez, Schmidt, & Watson, 2010), significantly mediated the aforementioned associations of depression with reduced alcohol consumption, as well as increased alcohol use problems in early adulthood. These findings may highlight the critical role of neuroticism in the relationship between depression and alcohol use (Lahey, 2009). On the one hand, individuals with high neuroticism may be more likely to engage in alcohol use as a maladaptive coping mechanism to regulate distressing emotions, eventually leading to alcohol abuse (Bolton, Robinson, & Sareen, 2009; Khantzian, 1997); on the other hand, however, alcohol consumption might alleviate depression by suppressing neurotic behavior, such as anxiety and irritability (Bolton, Robinson, & Sareen, 2009), which is further supported by neuroticism’s mediating effect on the causal influence of alcohol consumption on depression (Supplementary Table S9). It has been proposed that the coupling of alcohol consumption and social behavior, such as parties or nightclubs (O’Malley & Johnston, 2002), may contribute to the alleviation effect. In summary, neuroticism, as a stable psychological trait (Kotov, Gamez, Schmidt, & Watson, 2010), may play a central role in the complex interplay between depression and alcohol use.

Several studies have reported sex differences in the relationship between depression and alcohol use (Needham, 2007). Our findings indicate that males exhibit a positive correlation between depression and alcohol use problems at FU1 and FU3. In contrast, females show a consistent positive correlation between depression and alcohol use problems at all four timepoints, along with a significant alleviating effect of alcohol consumption on depression at FU3. This suggests that the impact of alcohol use on depression may differ between males and females at different ages. A potential explanation is that males tend to consume significantly higher quantities of alcohol than females (Supplementary Table S6), which may minimize any potential therapeutic effect. Another possible reason is that as individuals transition into adulthood, the underlying causes of depression may differ between males and females (Needham, 2007), making depressive symptoms in males less responsive to alcohol consumption. This aligns with previous literature indicating that males are less likely to use alcohol as a means of regulating negative emotions (Nolen-Hoeksema, 2004). While our study suggests a self-medication effect of alcohol, it is important to acknowledge the complexity of these causal relationships, as consistent effects were not observed at every time point. Therefore, future studies and clinical trials should focus on verifying the potential alleviation effects of moderate alcohol consumption on depression across candidate subpopulations, such as age and sex, which may help advance our understanding of the underlying mechanisms and identify the optimal time window for maximizing intervention efforts.

The study has several limitations. First, we chose to use the GWAS by Schumann et al. (2016) as our discovery dataset instead of larger GWAS such as that from the GWAS & Sequencing Consortium of Alcohol and Nicotine use (Saunders et al., 2022). While the GSCAN ‘drinks per week’ measure is also continuous and hence a plausible candidate, along with Schumann et al., the choice of a discovery GWAS for MR depends not only on sample size but also on the validation of genetic associations and predictive power in the target sample. Our evaluation revealed that the PRS from Schumann et al.’s GWAS demonstrated superior utility in our specific context, whereas the PRS calculated from the similarly European-ancestry GSCAN GWAS could not serve as a valid instrumental variable (IV) (Supplementary Table S18). Nevertheless, we acknowledge that investigating this topic further with a broader set of suitable GWAS resources is an important direction for future research. Second, the persistent longitudinal correlation between depression and alcohol use problems may result from bidirectional causal effects reinforcing both phenotypes, as suggested by previous animal models and human studies (Nurnberger et al., 2002; Rezvani, Parsian, & Overstreet, 2002). However, due to the overall low prevalence of alcohol abuse in our sample, coupled with a high dropout rate among individuals with high AUDIT scores, we were unable to establish a valid instrumental variable for AUD and thus could not fully verify its putative causal relationship with depression. Future research should aim to examine this relationship in a cohort with a higher prevalence of alcohol use problems. Third, at certain timepoints in the MR analysis, the effect was not significant for either males or females, possibly due to limited sample sizes. Furthermore, additional data from older age groups (e.g. 30–80 years) are needed to better understand the long-term impact of self-medication behavior.

## Conclusion

Our findings provide new insights into the causal interplay between alcohol use and depression, emphasizing the likely causal effect of depression on alcohol use problems and the potential alleviating effect of moderate alcohol consumption on depression in early adulthood, especially in females. Our research enhances the understanding and management of comorbid alcohol use problems and depression, emphasizing the importance of regulating alcohol consumption to improve early intervention strategies, including self-medication attempts. Future studies should focus on identifying populations that may benefit from these findings and investigating how the risk of alcohol use problems evolves, given that depression may contribute to its development.

## Supporting information

10.1017/S0033291725102444.sm001 Wang et al. supplementary material Wang et al. supplementary material

## Data Availability

The IMAGEN data are available from a dedicated database: https://imagen-project.org. The HCP data are available from a dedicated database: https://www.humanconnectome.org.

## Supplementary material

The supplementary material for this article can be found at http://doi.org/10.1017/S0033291725102444.
