# Supplementary material for: The causal interplay between depression and alcohol use from adolescence to young adulthood: a Mendelian randomization study
Source: Psychol Med. 2026 Jan 27;56:e28. doi: 10.1017/S0033291725102444 (PMC12885341; doi:10.1017/S0033291725102444)
Supplement: Wang et al. supplementary material [file S0033291725102444sup001.docx]

**The causal interplay between depression and alcohol use from adolescence to young adulthood: a Mendelian randomisation study**

**Supplementary Materials**

**Method S1.** Measurement of depressive symptoms

**Method S2.** Measurement of alcohol use

**Method S3.** Measurement of personality

**Table S1.** The measurement of depressive symptom scores

**Table S2.** The item content in the Alcohol Use Disorders Identification Test (AUDIT)

**Table S3.** The item content in the Substance Use Risk Profile Scale (SURPS)

**Table S4.** The item content in the Anxiety Screening (ANXDX) scale

**Table S5.** The item content in the NEO Personality Inventory

**Table S6.** Characteristics of the males and females in the IMAGEN cohort

**Table S7.** The results of the IMAGEN cohort retention and missingness

**Table S8.** The correlation between depression and alcohol consumption/alcohol use problems in the IMAGEN cohort

**Table S9.** The mediation effects between alcohol consumption/alcohol use problems and depression in IMAGEN cohort

**Table S10.** The fit measures for the cross-lagged panel model in the IMAGEN cohort

**Table S11.** The results of improved Mendelian randomisation analysis in the IMAGEN cohort

**Table S12.** The results of improved Mendelian randomisation analysis in the IMAGEN cohort after excluding 19 individuals who never initiated alcohol use across all four waves

**Table S13.** The correlation between alcohol consumption/alcohol use problems and depression in the HCP cohort

**Table S14.** The results of improved Mendelian randomisation analysis in the HCP cohort with adjustment for age and sex

**Table S15.** The results of improved Mendelian Randomisation analysis in the HCP cohort with adjustment for age, sex, and top 10 principal components

**Table S16.** The mediation effects between valid-PRSs and alcohol/depression in the IMAGEN cohort

**Table S17.** Top SNPs (n = 10) for Alcohol Consumption GWAS and MDD GWAS

**Table S18.** The correlation between the GSCAN-based Polygenic Risk Score and alcohol consumption in the IMAGEN cohort

**Figure S1.** The cumulative adjusted Eta-square between Sites/Sex and the Top 10 genetic principal components in the IMAGEN cohort

**Figure S2.** Random-intercepts cross-lagged panel model of depression scores and alcohol consumption/alcohol use problems from ages 14 to 23 years in the IMAGEN cohort, adjusted for the other alcohol use behaviour (n = 2093)

**Figure S3.** Random-intercepts cross-lagged panel model of depression scores and alcohol consumption/alcohol use problems from ages 14 to 23 years in the IMAGEN cohort (controlled for the other alcohol use behaviour) (n = 932 with complete longitudinal data at all four time points)

**Figure S4.** Results of two-sample Mendelian randomisation analyses

**References**

**Method S1. Measurement of depressive symptoms**

The depressive symptoms of the IMAGEN participants at all ages were assessed with an integrative score from the Development and Well-Being Assessment (Goodman, Ford, Richards, Gatward, & Meltzer, 2000) (DAWBA; 6 items) and the Strengths and Difficulties Questionnaire (Goodman, 2001) (SDQ; 2 items) (Table S1) (Xie et al., 2021). DAWBA is a wide-ranging psychiatric screening questionnaire and has previously been used to define subthreshold clinical symptoms in neuroimaging studies of subclinical psychopathology (Vulser et al., 2015). The self-reported SDQ was also employed in the current investigation, as it aids in assigning diagnostic status within the DAWBA framework.

**Method S2. Measurement of alcohol use**

The alcohol use behaviour of the IMAGEN participants was assessed using the screening questions from the Alcohol Use Disorders Identification Test (AUDIT) (Allen, Litten, Fertig, & Babor, 1997). The AUDIT was developed by the World Health Organisation as a simple way to screen and identify people who are at risk of developing alcohol problems. The AUDIT test focuses on identifying the preliminary signs of Alcohol Consumption and Alcohol Use Problems (Table S2). It is used to detect alcohol problems experienced within the last year. It is one of the most accurate alcohol screening tests available (Cook, Chung, Kelly, & Clark, 2005).

**Method S3. Measurement of personality**

In this study, the risk of personality for substance abuse were assessed using the Substance Use Risk Profile Scale (SURPS) (Table S3) (Woicik, Stewart, Pihl, & Conrod, 2009). This scale comprises four subscale components, namely 'Anxiety Sensitivity', 'Impulsivity', 'Hopelessness' and 'Sensation Seeking'. Additionally, we conducted an assessment using the Anxiety Screening (ANXDX; out of the Composite Diagnostic Interview DIA-X/M-CIDI) scale (Knappe et al., 2014; Lebeau et al., 2012) (Table S4) and included it in the mediation analysis. Externalizing symptoms (SDQ: Conduct problems scores and Hyperactivity scores) and socioeconomic status (Family stresses: Socioeconomic/housing score) were measured by DAWBA (Goodman et al., 2000). Personality traits were assessed using the NEO Personality Inventory (NEO–PI) (Table S5) (Costa & McCrae, 1992).

**Table S1. The measurement of depressive symptom scores**

| **Num.** | **Items (1-6 DAWBA, 7-8 SDQ)** | **Rating (0: No, 1: Yes)** | |
| --- | --- | --- | --- |
| 1 | Depression.sad | 0 | 1 |
| 2 | Depression.irritable | 0 | 1 |
| 3 | Depression.loss.of.interest | 0 | 1 |
| 4 | Depression.recent.talk.of.dsh | 0 | 1 |
| 5 | Depression.dsh.recently | 0 | 1 |
| 6 | Depression.dsh.ever | 0 | 1 |
| 7 | Headache/stomach ach | 0 | 1 |
| 8 | Unhappy | 0 | 1 |

Abbreviations: dsh, Deliberate self-harm

**Table S2. The item content in the Alcohol Use Disorders Identification Test (AUDIT)**

| **Categories** | **Items** | **Content** | **Rating** |
| --- | --- | --- | --- |
| Alcohol Consumption | Alcohol Use Frequency | How often do you have a drink containing alcohol? | 0 'Never'  1 'Monthly or less'  2 'Two to four times a month'  3 'Two to three times a week'  4 'Four or more times a week' |
|  | Typical Daily Quantity | How many drinks containing alcohol do you have on a typical day when you are drinking? | -1 'Never'  0 '1 or 2'  1 '3 or 4'  2 '5 or 6'  3 '7 to 9'  4 '10 or more' |
|  | Binge-drinking Frequency | How often do you have six or more drinks on one occasion? | 0 'Never'  1 'Less than monthly'  2 'Monthly'  3 'Weekly'  4 'Daily or almost daily |
| Alcohol Use Problems | Cannot Stop Drinking | How often during the last year have you found that you were not able to stop drinking once you had started? | 0 'Never'  1 'Less than monthly'  2 'Monthly'  3 'Weekly'  4 'Daily or almost daily |
|  | Failure because of Drinking | How often during the last year have you failed to do what was normally expected from you because of drinking? | 0 'Never'  1 'Less than monthly'  2 'Monthly'  3 'Weekly'  4 'Daily or almost daily |
|  | Start with a Drink | How often during the last year have you needed a first drink in the morning to get yourself going after a heavy drinking session? | 0 'Never'  1 'Less than monthly'  2 'Monthly'  3 'Weekly'  4 'Daily or almost daily |
|  | Feel Guilty after Drinking | How often during the last year have you had a feeling of guilt or remorse after drinking? | 0 'Never'  1 'Less than monthly'  2 'Monthly'  3 'Weekly'  4 'Daily or almost daily |
|  | Blackout because of Drinking | How often during the last year have you been unable to remember what happened the night before because you had been drinking? | 0 'Never'  1 'Less than monthly'  2 'Monthly'  3 'Weekly'  4 'Daily or almost daily |
|  | Injury because of Drinking | Have you or someone else been injured as a result of your drinking? | 0 'No'  2 'Yes, but not in the last year'  4 'Yes, during the last year' |
|  | Concerns from Others | Has a relative or friend, or a doctor or other health worker been concerned about your drinking or suggested you cut down? | 0 'No'  2 'Yes, but not in the last year'  4 'Yes, during the last year' |

**Table S3. The item content in the Substance Use Risk Profile Scale (SURPS)**

| **Items** | **Content** | **strongly disagree** | **disagree** | **agree** | **strongly agree** |
| --- | --- | --- | --- | --- | --- |
| Anxiety Sensitivity | It’s frightening to feel dizzy or faint. | 1 | 2 | 3 | 4 |
|  | It frightens me when I feel my heart beat change. | 1 | 2 | 3 | 4 |
|  | I get scared when I’m too nervous. | 1 | 2 | 3 | 4 |
|  | I get scared when I experience unusual body sensations. | 1 | 2 | 3 | 4 |
|  | It scares me when I’m unable to focus on a task. | 1 | 2 | 3 | 4 |
| Hopelessness | I am content. | 4 | 3 | 2 | 1 |
|  | I am happy. | 4 | 3 | 2 | 1 |
|  | I have faith that my future holds great promise. | 4 | 3 | 2 | 1 |
|  | I feel proud of my accomplishments. | 4 | 3 | 2 | 1 |
|  | I feel that I’m a failure. | 1 | 2 | 3 | 4 |
|  | I feel pleasant. | 4 | 3 | 2 | 1 |
|  | I am very enthusiastic about my future. | 1 | 2 | 3 | 4 |
| Impulsivity | I often don’t think things through before I speak. | 1 | 2 | 3 | 4 |
|  | I often involve myself in situations that I later regret being involved in. | 1 | 2 | 3 | 4 |
|  | I usually act without stopping to think. | 1 | 2 | 3 | 4 |
|  | Generally, I am an impulsive person. | 1 | 2 | 3 | 4 |
|  | I feel I have to be manipulative to get what I want. | 1 | 2 | 3 | 4 |
| Sensation Seeking | I would like to skydive. | 1 | 2 | 3 | 4 |
|  | I enjoy new and exciting experiences even if they are unconventional. | 1 | 2 | 3 | 4 |
|  | I like doing things that frighten me a little. | 1 | 2 | 3 | 4 |
|  | I would like to learn how to drive a motorcycle. | 1 | 2 | 3 | 4 |
|  | I would enjoy hiking long distances in wild and uninhabited territory. | 1 | 2 | 3 | 4 |

**Table S4. The item content in the Anxiety Screening (ANXDX) scale**

| **Num.** | **Items** | **Rating (0: Never, 1: Occasionally, 2: Half of the time, 3: Most of the Time, 4: All of the time)** | | | | |
| --- | --- | --- | --- | --- | --- | --- |
| 1 | felt moments of sudden terror, fear or fright | 0 | 1 | 2 | 3 | 4 |
| 2 | felt anxious, worried, or nervous | 0 | 1 | 2 | 3 | 4 |
| 3 | had thoughts of bad things happening | 0 | 1 | 2 | 3 | 4 |
| 4 | felt a racing heart, sweaty, trouble breathing, faint, or shaky | 0 | 1 | 2 | 3 | 4 |
| 5 | felt tense muscles, on edge or restless, or trouble relaxing or sleeping | 0 | 1 | 2 | 3 | 4 |
| 6 | avoided, or did not approach or enter, situations that made me anxious | 0 | 1 | 2 | 3 | 4 |
| 7 | moved away from or left situations early, remained near exits, or participated only minimally because of anxiety | 0 | 1 | 2 | 3 | 4 |
| 8 | not taken care of important issues (e.g. work, private, leisure) because of my anxiety, worry or fear | 0 | 1 | 2 | 3 | 4 |
| 9 | spent a lot of time preparing for, or procrastinating about  (putting off), things because of anxiety | 0 | 1 | 2 | 3 | 4 |
| 10 | distracted myself to avoid thinking about things that made me anxious | 0 | 1 | 2 | 3 | 4 |
| 11 | needed help to cope with anxiety (e.g., alcohol or medications, superstitious objects, other people) | 0 | 1 | 2 | 3 | 4 |
| 12 | felt stressed and down because of my anxiety, worry or fear | 0 | 1 | 2 | 3 | 4 |

**Table S5. The item content in the NEO Personality Inventory**

| **Items** | **Content** | **Strongly Disagree** | **Disagree** | **Neutral** | **Agree** | **Strongly Agree** |
| --- | --- | --- | --- | --- | --- | --- |
| Neuroticism | I am not a worrier. | 4 | 3 | 2 | 1 | 0 |
|  | I often feel that I’m not as good as others. | 0 | 1 | 2 | 3 | 4 |
|  | When I’m under a great deal of stress, sometimes I feel like I’m going to pieces. | 0 | 1 | 2 | 3 | 4 |
|  | I rarely feel lonely or blue. | 4 | 3 | 2 | 1 | 0 |
|  | I often feel tense and jittery. | 0 | 1 | 2 | 3 | 4 |
|  | Sometimes I feel completely worthless. | 0 | 1 | 2 | 3 | 4 |
|  | I rarely feel fearful or anxious. | 4 | 3 | 2 | 1 | 0 |
|  | I often get angry at the way people treat me. | 0 | 1 | 2 | 3 | 4 |
|  | Too often, when things go wrong, I get discouraged and feel like giving up. | 0 | 1 | 2 | 3 | 4 |
|  | I am seldom sad or depressed. | 4 | 3 | 2 | 1 | 0 |
|  | I often feel helpless and want someone else to solve my problems. | 0 | 1 | 2 | 3 | 4 |
|  | At times I have been so ashamed I just wanted to hide. | 0 | 1 | 2 | 3 | 4 |
| Extraversion | I like to have a lot of people around me. | 0 | 1 | 2 | 3 | 4 |
|  | I laugh easily. | 0 | 1 | 2 | 3 | 4 |
|  | I’m not happy-go-lucky. | 4 | 3 | 2 | 1 | 0 |
|  | I really enjoy talking to people. | 0 | 1 | 2 | 3 | 4 |
|  | I like to be where the action is. | 0 | 1 | 2 | 3 | 4 |
|  | I usually prefer to do things alone. | 4 | 3 | 2 | 1 | 0 |
|  | I often feel as if I’m bursting with energy. | 0 | 1 | 2 | 3 | 4 |
|  | I am a cheerful, high-spirited person. | 0 | 1 | 2 | 3 | 4 |
|  | I am not a cheerful optimist. | 4 | 3 | 2 | 1 | 0 |
|  | My life is fast-paced. | 0 | 1 | 2 | 3 | 4 |
|  | I am a very active person. | 0 | 1 | 2 | 3 | 4 |
|  | I would rather go my own way than be a leader of others. | 4 | 3 | 2 | 1 | 0 |
| Openness | I don’t like to waste my time daydreaming. | 4 | 3 | 2 | 1 | 0 |
|  | Once I find the right way to do something, I stick to it. | 4 | 3 | 2 | 1 | 0 |
|  | I am intrigued by the patterns I find in art and nature. | 0 | 1 | 2 | 3 | 4 |
|  | I believe letting students hear controversial speakers can only confuse and mislead them. | 4 | 3 | 2 | 1 | 0 |
|  | Poetry has little or no effect on me. | 4 | 3 | 2 | 1 | 0 |
|  | I often try new and foreign foods. | 0 | 1 | 2 | 3 | 4 |
|  | I seldom notice the moods or feelings that different environments produce. | 4 | 3 | 2 | 1 | 0 |
|  | I believe we should look to our religious authorities for decisions on moral issues. | 4 | 3 | 2 | 1 | 0 |
|  | Sometimes when I am reading poetry or looking at a work of art, I feel a chill or wave of excitement. | 0 | 1 | 2 | 3 | 4 |
|  | I have little interest in speculating on the nature of the universe or human condition. | 4 | 3 | 2 | 1 | 0 |
|  | I have a lot of intellectual curiosity. | 0 | 1 | 2 | 3 | 4 |
|  | I often enjoy playing with theories or abstract ideas. | 0 | 1 | 2 | 3 | 4 |
| Agreeableness | I try to be courteous to everyone I meet. | 0 | 1 | 2 | 3 | 4 |
|  | I often get into arguments with my family and co-workers. | 4 | 3 | 2 | 1 | 0 |
|  | Some people think I’m selfish and egotistical. | 4 | 3 | 2 | 1 | 0 |
|  | I would rather cooperate with others than compete with them. | 0 | 1 | 2 | 3 | 4 |
|  | Often, people aren’t as nice as they seem to be. | 4 | 3 | 2 | 1 | 0 |
|  | I believe that most people will take advantage of you if you let them. | 4 | 3 | 2 | 1 | 0 |
|  | Most people I know like me. | 0 | 1 | 2 | 3 | 4 |
|  | Some people think of me as cold and calculating. | 4 | 3 | 2 | 1 | 0 |
|  | I don’t worry much about the homeless. | 4 | 3 | 2 | 1 | 0 |
|  | I generally try to be thoughtful and considerate. | 0 | 1 | 2 | 3 | 4 |
|  | If I don’t like people, I let them know it. | 4 | 3 | 2 | 1 | 0 |
|  | If necessary, I am willing to manipulate people to get what I want. | 4 | 3 | 2 | 1 | 0 |
| Conscientiousness | I keep my belongings neat and clean. | 0 | 1 | 2 | 3 | 4 |
|  | I’m pretty good about pacing myself so as to get things done on time. | 0 | 1 | 2 | 3 | 4 |
|  | I’m not a very orderly or methodical person. | 4 | 3 | 2 | 1 | 0 |
|  | I try to perform all the tasks assigned to me conscientiously. | 0 | 1 | 2 | 3 | 4 |
|  | I have a clear set of goals and work toward them in an orderly fashion. | 0 | 1 | 2 | 3 | 4 |
|  | I waste a lot of time before settling down to work. | 4 | 3 | 2 | 1 | 0 |
|  | I work hard to accomplish my goals. | 0 | 1 | 2 | 3 | 4 |
|  | When I make a commitment, I can always be counted on to follow  through. | 0 | 1 | 2 | 3 | 4 |
|  | Sometimes I’m not as dependable or reliable as I should be. | 4 | 3 | 2 | 1 | 0 |
|  | I am a productive person who always gets the job done. | 0 | 1 | 2 | 3 | 4 |
|  | I never seem to be able to get organised. | 4 | 3 | 2 | 1 | 0 |
|  | I strive for excellence in everything I do. | 0 | 1 | 2 | 3 | 4 |

**Table S6. Characteristics of the males and females in the IMAGEN cohort**

| **Depression scores (Males vs. Females)** | | | | |
| --- | --- | --- | --- | --- |
| **Time** | **Mean ± SD (Males)** | **Mean ± SD (Females)** | ***t* value** | ***p* value** |
| Baseline | 1.51 ± 1.56 | 2.56 ± 1.99 | -13.52 | **5.50E-40** |
| Follow-up 1 | 1.55 ± 1.73 | 2.90 ± 2.22 | -13.62 | **3.90E-40** |
| Follow-up 2 | 1.15* ± 1.57 | 2.23* ± 2.06 | -10.51 | **6.80E-25** |
| Follow-up 3 | 1.13* ± 1.62 | 2.00* ± 1.98 | -8.51 | **4.93E-17** |
| **Alcohol consumption (Males vs. Females)** | | | | |
| **Time** | **Mean ± SD (Males)** | **Mean ± SD (Females)** | ***t* value** | ***p* value** |
| Baseline | 1.05 ± 1.50 | 1.10 ± 1.52 | -0.74 | 0.46 |
| Follow-up 1 | 3.24 ± 2.53 | 2.52 ± 2.05 | 6.71 | **2.65E-11** |
| Follow-up 2 | 4.77 ± 2.61 | 3.75 ± 2.38 | 8.46 | **6.36E-17** |
| Follow-up 3 | 4.69 ± 2.32 | 3.66 ± 2.07 | 8.89 | **1.94E-18** |
| **Alcohol use problems (Males vs. Females)** | | | | |
| **Time** | **Mean ± SD (Males)** | **Mean ± SD (Females)** | ***t* value** | ***p* value** |
| Baseline | 0.44 ± 1.66 | 0.48 ± 1.50 | -0.57 | 0.57 |
| Follow-up 1 | 1.11 ± 2.12 | 0.98 ± 2.06 | 1.23 | 0.22 |
| Follow-up 2 | 1.82 ± 2.84 | 1.50 ± 2.76 | 2.45 | **0.01** |
| Follow-up 3 | 2.35 ± 3.26 | 1.57 ± 2.68 | 4.98 | **7.23E-07** |

* The Depression Scores were the sum of 8 items, with 'irritable' missing during both FU2 and FU3, resulting in the sum of 7 items.

**Table S7. The results of the IMAGEN cohort retention and missingness**

| **Depression Scores** | | **Total N at baseline** | | **N retained at follow-up** | | **N dropped** | | **Attrition rate** | | **Depression** | | | | **Sex** | | | |
| --- | --- | --- | --- | --- | --- | --- | --- | --- | --- | --- | --- | --- | --- | --- | --- | --- | --- |
|  |  |  |  |  |  |  |  |  |  | ***t***  **value** | | ***p* value** | | **X-squared** | | ***p***  **value** | |
| **BL-FU1** | | 2109 | | 1657 | | 452 | | 21.4% | | 1.678 | | 0.094 | | 2.175 | | 0.1403 | |
| **BL-FU2** | | 2109 | | 1350 | | 759 | | 36.0% | | 0.502 | | 0.616 | | 9.734 | | 0.0018 | |
| **BL-FU3** | | 2109 | | 1279 | | 830 | | 39.4% | | 1.275 | | 0.202 | | 8.958 | | 0.0028 | |
|  | | | | | | | | | | | | | | | | | |
| **AUDIT** | **Total N at baseline** | | **N retained at follow-up** | | **N dropped** | **Attrition rate** | **Alcohol Consumption** | | | | **Alcohol Use Problems** | | | | **Sex** | | |
|  |  |  |  |  |  |  | ***t***  **value** | | ***p***  **value** | | ***t***  **value** | | ***p***  **value** | | **X-squared** | | ***p***  **value** |
| **BL-FU1** | 2110 | | 1665 | | 445 | 21.1% | 3.680 | | 3E-04 | | 3.310 | | 1E-03 | | 2.055 | | 0.1517 |
| **BL-FU2** | 2110 | | 1481 | | 629 | 29.8% | 3.793 | | 2E-04 | | 4.307 | | 0.000 | | 5.784 | | 0.0168 |
| **BL-FU3** | 2110 | | 1328 | | 782 | 37.1% | 3.213 | | 0.0013 | | 3.809 | | 1E-04 | | 8.473 | | 0.0036 |

**Table S8. The correlation between depression and alcohol consumption/alcohol use problems in the IMAGEN cohort.** All *p*-values and confidence intervals are reported as two-tailed.

| **Correlation between Depression Scores and Alcohol Consumption** | | | | | | |
| --- | --- | --- | --- | --- | --- | --- |
| **Controlled for sex and sites** | | | | | | |
| **Timepoint** | ***r (all)***  **[95% CI]** | ***p (all)*** | ***r (male)***  **[95% CI]** | ***p (male)*** | ***r (female)***  **[95% CI]** | ***p (female)*** |
| **Baseline** | 0.141  [0.098, 0.182] | **1.12E-10** | 0.064  [0.003, 0.125] | **0.039** | 0.195  [0.136, 0.252] | **1.61E-10** |
| **Follow-up 1** | 0.151  [0.103, 0.199] | **9.01E-10** | 0.088  [0.018, 0.157] | **0.014** | 0.218  [0.153, 0.282] | **1.84E-10** |
| **Follow-up 2** | 0.100  [0.046, 0.153] | **2.72E-04** | -1.58E-04  [-0.080, 0.079] | 0.997 | 0.176  [0.103, 0.246] | **2.57E-06** |
| **Follow-up 3** | -0.027  [-0.082, 0.028] | 0.336 | -0.053  [-0.134, 0.028] | 0.200 | -0.008  [-0.083, 0.068] | 0.841 |
| **Controlled for sex, sites, and Alcohol Use Problems** | | | | | | |
| **Timepoint** | ***r (all)***  **[95% CI]** | ***p (all)*** | ***r (male)***  **[95% CI]** | ***p (male)*** | ***r (female)***  **[95% CI]** | ***p (female)*** |
| **Baseline** | 0.094  [0.052, 0.137] | **1.58E-05** | 0.042  [-0.020, 0.103] | 0.184 | 0.125  [0.065, 0.184] | **4.42E-05** |
| **Follow-up 1** | 0.085  [0.037, 0.133] | **6.08E-04** | 0.031  [-0.039, 0.102] | 0.382 | 0.144  [0.077, 0.210] | **2.85E-05** |
| **Follow-up 2** | 0.050  [-0.004, 0.103] | 0.070 | -0.019  [-0.098, 0.061] | 0.644 | 0.104  [0.031, 0.177] | **0.005** |
| **Follow-up 3** | -0.113  [-0.167, -0.059] | **5.35E-05** | -0.145  [-0.223, -0.064] | **4.44E-04** | -0.097  [-0.171, -0.021] | **0.012** |
| **Controlled for sex, sites, Alcohol Use Problems, and externalizing symptoms** | | | | | | |
| **Timepoint** | ***r (all)***  **[95% CI]** | ***p (all)*** | ***r (male)***  **[95% CI]** | ***p (male)*** | ***r (female)***  **[95% CI]** | ***p (female)*** |
| **Baseline** | 0.051  [0.008, 0.093] | **0.021** | 0.007  [-0.054, 0.069] | 0.820 | 0.070  [0.010, 0.130] | **0.022** |
| **Follow-up 1** | 0.030  [-0.018, 0.079] | 0.222 | -0.009  [-0.079, 0.062] | 0.807 | 0.074  [0.006, 0.141] | **0.033** |
| **Follow-up 2** | 0.021  [-0.033, 0.075] | 0.444 | -0.028  [-0.107, 0.052] | 0.491 | 0.054  [-0.020, 0.127] | 0.154 |
| **Follow-up 3** | -0.104  [-0.158, -0.049] | **2.10E-04** | -0.120  [-0.199, -0.039] | **0.004** | -0.105  [-0.180, -0.030] | **6.21E-03** |
| **Controlled for sex, sites, Alcohol Use Problems, and socioeconomic status** | | | | | | |
| **Timepoint** | ***r (all)***  **[95% CI]** | ***p (all)*** | ***r (male)***  **[95% CI]** | ***p (male)*** | ***r (female)***  **[95% CI]** | ***p (female)*** |
| **Baseline** | 0.091  [0.048, 0.133] | **3.41E-05** | 0.037  [-0.024, 0.099] | 0.236 | 0.122  [0.062, 0.181] | **7.06E-05** |
| **Follow-up 1** | 0.090  [0.042, 0.139] | **2.89E-04** | 0.031  [-0.040, 0.101] | 0.389 | 0.153  [0.086, 0.218] | **9.90E-06** |
| **Follow-up 2** | 0.055  [0.002, 0.109] | **0.044** | -0.014  [-0.094, 0.066] | 0.729 | 0.110  [0.036, 0.182] | **3.54E-03** |
| **Follow-up 3** | -0.109  [-0.164, -0.055] | **1.05E-04** | -0.142  [-0.221, -0.061] | **6.22E-04** | -0.090  [-0.165, -0.015] | **0.020** |
| **Controlled for sex, sites, Alcohol Use Problems, and personality traits** | | | | | | |
| **Timepoint** | ***r (all)***  **[95% CI]** | ***p (all)*** | ***r (male)***  **[95% CI]** | ***p (male)*** | ***r (female)***  **[95% CI]** | ***p (female)*** |
| **Baseline** | 0.086  [0.043, 0.129] | **8.33E-05** | 0.060  [-0.001, 0.122] | 0.054 | 0.090  [0.030, 0.150] | **3.33E-03** |
| **Follow-up 1** | 0.104  [0.055, 0.152] | **2.98E-05** | 0.064  [-0.007, 0.134] | 0.077 | 0.143  [0.076, 0.209] | **3.61E-05** |
| **Follow-up 2** | 0.104  [0.050, 0.157] | **1.54E-04** | 0.045  [-0.035, 0.125] | 0.267 | 0.141  [0.068, 0.213] | **1.73E-04** |
| **Follow-up 3** | -0.003  [-0.059, 0.052] | 0.904 | -0.053  [-0.133, 0.029] | 0.208 | 0.034  [-0.042, 0.110] | 0.376 |

| **Correlation between Depression Scores and Alcohol Use Problems** | | | | | | |
| --- | --- | --- | --- | --- | --- | --- |
| **Controlled for sex and sites** | | | | | | |
| **Timepoint** | ***r (all)***  **[95% CI]** | ***p (all)*** | ***r (male)***  **[95% CI]** | ***p (male)*** | ***r (female)***  **[95% CI]** | ***p (female)*** |
| **Baseline** | 0.113  [0.070, 0.155] | **2.28E-07** | 0.054  [-0.007, 0.115] | 0.084 | 0.166  [0.107, 0.224] | **5.72E-08** |
| **Follow-up 1** | 0.155  [0.108, 0.203] | **3.21E-10** | 0.117  [0.047, 0.186] | **0.001** | 0.193  [0.126, 0.257] | **1.95E-08** |
| **Follow-up 2** | 0.114  [0.061, 0.167] | **3.16E-05** | 0.032  [-0.048, 0.111] | 0.433 | 0.173  [0.101, 0.244] | **3.54E-06** |
| **Follow-up 3** | 0.129  [0.074, 0.182] | **4.43E-06** | 0.128  [0.047, 0.207] | **0.002** | 0.139  [0.064, 0.212] | **3.03E-04** |
| **Controlled for sex, sites, and Alcohol Consumption** | | | | | | |
| **Timepoint** | ***r (all)***  **[95% CI]** | ***p (all)*** | ***r (male)***  **[95% CI]** | ***p (male)*** | ***r (female)***  **[95% CI]** | ***p (female)*** |
| **Baseline** | 0.043  (0, 0.086] | 0.051 | 0.022  [-0.039, 0.084] | 0.474 | 0.071  [0.010, 0.130] | **0.022** |
| **Follow-up 1** | 0.092  [0.044, 0.140] | **2.07E-04** | 0.083  [0.013, 0.153] | **0.020** | 0.100  [0.033, 0.167] | **3.67E-03** |
| **Follow-up 2** | 0.074  [0.021, 0.128] | **6.76E-03** | 0.037  [-0.043, 0.116] | 0.363 | 0.100  [0.027, 0.173] | **7.59E-03** |
| **Follow-up 3** | 0.169  [0.115, 0.222] | **1.57E-09** | 0.185  [0.105, 0.262] | **6.70E-06** | 0.168  [0.094, 0.241] | **1.11E-05** |
| **Controlled for sex, sites, Alcohol Consumption, and externalizing symptoms** | | | | | | |
| **Timepoint** | ***r (all)***  **[95% CI]** | ***p (all)*** | ***r (male)***  **[95% CI]** | ***p (male)*** | ***r (female)***  **[95% CI]** | ***p (female)*** |
| **Baseline** | 0.013  [-0.030, 0.056] | 0.546 | -0.003  [-0.065, 0.058] | 0.917 | 0.035  [-0.025, 0.095] | 0.253 |
| **Follow-up 1** | 0.079  [0.031, 0.128] | **0.001** | 0.071  (0, 0.141] | **0.049** | 0.087  [0.019, 0.154] | **0.012** |
| **Follow-up 2** | 0.030  [-0.024, 0.084] | 0.276 | -0.012  [-0.091, 0.068] | 0.777 | 0.062  [-0.011, 0.136] | 0.097 |
| **Follow-up 3** | 0.084  [0.029, 0.138] | **2.82E-03** | 0.085  [0.004, 0.165] | **0.040** | 0.100  [0.025, 0.175] | **0.009** |
| **Controlled for sex, sites, Alcohol Consumption, and socioeconomic status** | | | | | | |
| **Timepoint** | ***r (all)***  **[95% CI]** | ***p (all)*** | ***r (male)***  **[95% CI]** | ***p (male)*** | ***r (female)***  **[95% CI]** | ***p (female)*** |
| **Baseline** | 0.043  (0, 0.085] | 0.053 | 0.023  [-0.038, 0.085] | 0.462 | 0.070  [0.009, 0.129] | **0.024** |
| **Follow-up 1** | 0.086  [0.038, 0.134] | **5.44E-04** | 0.078  [0.007, 0.148] | **0.031** | 0.093  [0.025, 0.160] | **0.007** |
| **Follow-up 2** | 0.069  [0.015, 0.122] | **0.012** | 0.035  [-0.045, 0.114] | 0.389 | 0.091  [0.017, 0.163] | **0.016** |
| **Follow-up 3** | 0.172  [0.118, 0.225] | **8.27E-10** | 0.187  [0.107, 0.264] | **6.23E-06** | 0.173  [0.098, 0.245] | **7.20E-06** |
| **Controlled for sex, sites, Alcohol Consumption, and personality traits** | | | | | | |
| **Timepoint** | ***r (all)***  **[95% CI]** | ***p (all)*** | ***r (male)***  **[95% CI]** | ***p (male)*** | ***r (female)***  **[95% CI]** | ***p (female)*** |
| **Baseline** | 0.015  [-0.028, 0.058] | 0.495 | -0.009  [-0.071, 0.053] | 0.777 | 0.046  [-0.014, 0.106] | 0.136 |
| **Follow-up 1** | 0.029  [-0.020, 0.078] | 0.245 | 0.012  [-0.059, 0.083] | 0.742 | 0.041  [-0.027, 0.109] | 0.242 |
| **Follow-up 2** | 0.003  [-0.051, 0.057] | 0.908 | -0.016  [-0.096, 0.064] | 0.698 | 0.005  [-0.069, 0.079] | 0.902 |
| **Follow-up 3** | 0.048  [-0.007, 0.103] | 0.088 | 0.082  (0, 0.162] | **0.049** | 0.016  [-0.060, 0.092] | 0.684 |

| **Correlation between Depression Scores and Alcohol Consumption** | | | | | | |
| --- | --- | --- | --- | --- | --- | --- |
| **Controlled for sex, sites, Alcohol Use Problems, and average neuroticism score** | | | | | | |
| **Timepoint** | ***r (all)***  **[95% CI]** | ***p (all)*** | ***r (male)***  **[95% CI]** | ***p (male)*** | ***r (female)***  **[95% CI]** | ***p (female)*** |
| **Baseline** | 0.098  [0.055, 0.140] | **8.35E-06** | 0.073  [0.012, 0.134] | **0.020** | 0.102  [0.042, 0.161] | **9.21E-04** |
| **Follow-up 1** | 0.106  [0.057, 0.154] | **2.06E-05** | 0.064  [-0.006, 0.134] | 0.074 | 0.145  [0.078, 0.211] | **2.61E-05** |
| **Follow-up 2** | 0.087  [0.034, 0.141] | **1.48E-03** | 0.037  [-0.043, 0.116] | 0.365 | 0.123  [0.049, 0.195] | **0.001** |
| **Follow-up 3** | -0.016  [-0.071, 0.039] | 0.566 | -0.056  [-0.136, 0.026] | 0.181 | 0.011  [-0.065, 0.087] | 0.777 |
| **Controlled for sex, sites, Alcohol Use Problems, and average extraversion score** | | | | | | |
| **Timepoint** | ***r (all)***  **[95% CI]** | ***p (all)*** | ***r (male)***  **[95% CI]** | ***p (male)*** | ***r (female)***  **[95% CI]** | ***p (female)*** |
| **Baseline** | 0.110  [0.068, 0.153] | **4.60E-07** | 0.053  [-0.008, 0.114] | 0.090 | 0.145  [0.085, 0.203] | **2.33E-06** |
| **Follow-up 1** | 0.137  [0.089, 0.184] | **3.50E-08** | 0.080  [0.010, 0.150] | **0.026** | 0.193  [0.127, 0.258] | **1.91E-08** |
| **Follow-up 2** | 0.109  [0.055, 0.162] | **7.55E-05** | 0.037  [-0.042, 0.117] | 0.359 | 0.158  [0.085, 0.229] | **2.54E-05** |
| **Follow-up 3** | -0.048  [-0.103, 0.007] | 0.087 | -0.104  [-0.183, -0.022] | **0.013** | -0.005  [-0.081, 0.070] | 0.890 |
| **Controlled for sex, sites, Alcohol Use Problems, and average openness score** | | | | | | |
| **Timepoint** | ***r (all)***  **[95% CI]** | ***p (all)*** | ***r (male)***  **[95% CI]** | ***p (male)*** | ***r (female)***  **[95% CI]** | ***p (female)*** |
| **Baseline** | 0.094  [0.051, 0.136] | **1.94E-05** | 0.038  [-0.023, 0.099] | 0.225 | 0.125  [0.066, 0.184] | **4.38E-05** |
| **Follow-up 1** | 0.087  [0.038, 0.135] | **4.97E-04** | 0.035  [-0.036, 0.105] | 0.338 | 0.145  [0.077, 0.211] | **2.77E-05** |
| **Follow-up 2** | 0.048  [-0.006, 0.101] | 0.083 | -0.024  [-0.103, 0.056] | 0.559 | 0.104  [0.030, 0.176] | **5.76E-03** |
| **Follow-up 3** | -0.113  [-0.167, -0.058] | **6.23E-05** | -0.146  [-0.224, -0.065] | **4.23E-04** | -0.096  [-0.171, -0.021] | **0.013** |
| **Controlled for sex, sites, Alcohol Use Problems, and average agreeableness score** | | | | | | |
| **Timepoint** | ***r (all)***  **[95% CI]** | ***p (all)*** | ***r (male)***  **[95% CI]** | ***p (male)*** | ***r (female)***  **[95% CI]** | ***p (female)*** |
| **Baseline** | 0.062  [0.020, 0.105] | **0.004** | 0.030  [-0.031, 0.092] | 0.334 | 0.071  [0.011, 0.131] | **0.020** |
| **Follow-up 1** | 0.075  [0.026, 0.123] | **2.73E-03** | 0.028  [-0.042, 0.099] | 0.433 | 0.124  [0.056, 0.190] | **3.35E-04** |
| **Follow-up 2** | 0.049  [-0.005, 0.102] | 0.077 | -0.015  [-0.094, 0.065] | 0.713 | 0.097  [0.023, 0.170] | **0.010** |
| **Follow-up 3** | -0.094  [-0.148, -0.039] | **8.31E-04** | -0.126  [-0.205, -0.045] | **0.002** | -0.080  [-0.154, -0.004] | **0.039** |
| **Controlled for sex, sites, Alcohol Use Problems, and average conscientiousness score** | | | | | | |
| **Timepoint** | ***r (all)***  **[95% CI]** | ***p (all)*** | ***r (male)***  **[95% CI]** | ***p (male)*** | ***r (female)***  **[95% CI]** | ***p (female)*** |
| **Baseline** | 0.072  [0.029, 0.115] | **9.82E-04** | 0.025  [-0.037, 0.086] | 0.434 | 0.099  [0.039, 0.158] | **0.001** |
| **Follow-up 1** | 0.053  [0.004, 0.102] | **0.033** | 0.012  [-0.058, 0.083] | 0.732 | 0.097  [0.029, 0.164] | **0.005** |
| **Follow-up 2** | 0.034  [-0.020, 0.087] | 0.222 | -0.032  [-0.111, 0.048] | 0.430 | 0.085  [0.011, 0.157] | **0.025** |
| **Follow-up 3** | -0.119  [-0.173, -0.065] | **2.13E-05** | -0.144  [-0.223, -0.064] | **4.79E-04** | -0.109  [-0.183, -0.034] | **0.005** |

| **Correlation between Depression Scores and Alcohol Use Problems** | | | | | | |
| --- | --- | --- | --- | --- | --- | --- |
| **Controlled for sex, sites, Alcohol Consumption, and average neuroticism score** | | | | | | |
| **Timepoint** | ***r (all)***  **[95% CI]** | ***p (all)*** | ***r (male)***  **[95% CI]** | ***p (male)*** | ***r (female)***  **[95% CI]** | ***p (female)*** |
| **Baseline** | 0.015  [-0.028, 0.058] | 0.482 | -0.010  [-0.071, 0.052] | 0.755 | 0.047  [-0.014, 0.107] | 0.128 |
| **Follow-up 1** | 0.033  [-0.015, 0.082] | 0.179 | 0.026  [-0.045, 0.096] | 0.470 | 0.042  [-0.026, 0.110] | 0.222 |
| **Follow-up 2** | 0.002  [-0.052, 0.056] | 0.940 | -0.013  [-0.093, 0.066] | 0.743 | 0.007  [-0.067, 0.081] | 0.855 |
| **Follow-up 3** | 0.054  [-0.002, 0.108] | 0.057 | 0.087  [0.006, 0.167] | **0.036** | 0.030  [-0.046, 0.106] | 0.436 |
| **Controlled for sex, sites, Alcohol Consumption, and average extraversion score** | | | | | | |
| **Timepoint** | ***r (all)***  **[95% CI]** | ***p (all)*** | ***r (male)***  **[95% CI]** | ***p (male)*** | ***r (female)***  **[95% CI]** | ***p (female)*** |
| **Baseline** | 0.040  [-0.003, 0.083] | 0.070 | 0.025  [-0.037, 0.086] | 0.430 | 0.063  [0.003, 0.123] | **0.041** |
| **Follow-up 1** | 0.093  [0.045, 0.142] | **1.72E-04** | 0.085  [0.014, 0.154] | **0.019** | 0.103  [0.035, 0.169] | **0.003** |
| **Follow-up 2** | 0.073  [0.019, 0.127] | **0.008** | 0.052  [-0.028, 0.131] | 0.201 | 0.081  [0.007, 0.154] | **0.031** |
| **Follow-up 3** | 0.165  [0.111, 0.218] | **3.65E-09** | 0.196  [0.117, 0.273] | **1.93E-06** | 0.144  [0.069, 0.218] | **1.75E-04** |
| **Controlled for sex, sites, Alcohol Consumption, and average openness score** | | | | | | |
| **Timepoint** | ***r (all)***  **[95% CI]** | ***p (all)*** | ***r (male)***  **[95% CI]** | ***p (male)*** | ***r (female)***  **[95% CI]** | ***p (female)*** |
| **Baseline** | 0.044  [0.001, 0.086] | **0.047** | 0.028  [-0.034, 0.089] | 0.372 | 0.068  [0.008, 0.128] | **0.027** |
| **Follow-up 1** | 0.088  [0.040, 0.137] | **3.77E-04** | 0.071  [0.001, 0.141] | **0.048** | 0.099  [0.032, 0.166] | **0.004** |
| **Follow-up 2** | 0.073  [0.019, 0.127] | **0.008** | 0.028  [-0.052, 0.108] | 0.489 | 0.102  [0.029, 0.175] | **0.006** |
| **Follow-up 3** | 0.167  [0.113, 0.220] | **2.32E-09** | 0.180  [0.100, 0.258] | **1.24E-05** | 0.172  [0.098, 0.245] | **7.25E-06** |
| **Controlled for sex, sites, Alcohol Consumption, and average agreeableness score** | | | | | | |
| **Timepoint** | ***r (all)***  **[95% CI]** | ***p (all)*** | ***r (male)***  **[95% CI]** | ***p (male)*** | ***r (female)***  **[95% CI]** | ***p (female)*** |
| **Baseline** | 0.030  [-0.013, 0.073] | 0.167 | 0.003  [-0.059, 0.064] | 0.928 | 0.067  [0.007, 0.127] | **0.029** |
| **Follow-up 1** | 0.067  [0.018, 0.115] | **0.007** | 0.065  [-0.006, 0.135] | 0.072 | 0.069  [0.001, 0.136] | **0.046** |
| **Follow-up 2** | 0.045  [-0.009, 0.099] | 0.102 | 0.021  [-0.059, 0.101] | 0.602 | 0.059  [-0.015, 0.132] | 0.120 |
| **Follow-up 3** | 0.151  [0.096, 0.204] | **7.47E-08** | 0.168  [0.088, 0.246] | **4.89E-05** | 0.151  [0.077, 0.225] | **8.16E-05** |
| **Controlled for sex, sites, Alcohol Consumption, and average conscientiousness score** | | | | | | |
| **Timepoint** | ***r (all)***  **[95% CI]** | ***p (all)*** | ***r (male)***  **[95% CI]** | ***p (male)*** | ***r (female)***  **[95% CI]** | ***p (female)*** |
| **Baseline** | 0.037  [-0.006, 0.080] | 0.088 | 0.017  [-0.045, 0.078] | 0.589 | 0.066  [0.006, 0.126] | **0.031** |
| **Follow-up 1** | 0.089  [0.040, 0.137] | **3.49E-04** | 0.078  [0.007, 0.148] | **0.030** | 0.101  [0.034, 0.168] | **0.003** |
| **Follow-up 2** | 0.055  [0.001, 0.109] | **0.046** | 0.011  [-0.069, 0.091] | 0.788 | 0.086  [0.012, 0.159] | **0.022** |
| **Follow-up 3** | 0.130  [0.076, 0.184] | **3.45E-06** | 0.148  [0.068, 0.227] | **3.29E-04** | 0.126  [0.051, 0.200] | **0.001** |

**Table S9. The mediation effects between alcohol consumption/alcohol use problems and depression in the IMAGEN cohort.** The *p*-values of the mediation effects were obtained with 10,000 times bootstrap samplings. All *p*-values and confidence intervals are reported as one-tailed.

| **The mediation effects between alcohol consumption and depression** | | | | | | | | | | | | |
| --- | --- | --- | --- | --- | --- | --- | --- | --- | --- | --- | --- | --- |
| **All people** | | | | | | | | | | | | |
| **Independent variable (X)** | **Mediation variable (M)** | **Dependent variable (Y)** | **Total effect** | ***p _total_*** | **Effect of X on M** | ***p***  ***_X on M_*** | **Effect of M on Y** | ***p***  ***_M on Y_*** | **Direct effect** | ***p _direct_*** | **Mediation effect [95% CI]** | ***p***  ***_mediation_*** |
| **BL Alcohol Consumption** | **BL Neuroticism** | **BL Depression Scores** | 0.159 | 2.6E-07 | 0.031 | 0.002 | 1.47 | 7.31E-102 | 0.114 | 1.57E-05 | 0.045 [0.019, ∞] | **0.0016** |
| **FU1 Alcohol Consumption** | **FU1 Neuroticism** | **FU1 Depression Scores** | 0.130 | 1.15E-09 | 0.017 | 0.007 | 1.78 | 1.06E-128 | 0.101 | 1.43E-08 | 0.030 [0.010, ∞] | **0.007** |
| **FU2 Alcohol Consumption** | **FU2 Neuroticism** | **FU2 Depression Scores** | 0.098 | 5.34E-05 | 0.019 | 0.017 | 1.77 | 2.57E-110 | 0.064 | 0.0006 | 0.033 [0.006, ∞] | **0.017** |
| **FU3 Alcohol Consumption** |  | **FU3 Depression Scores** | 0.001 | 0.484 |  |  |  |  |  |  |  |  |
| **Males** | | | | | | | | | | | | |
| **Independent variable (X)** | **Mediation variable (M)** | **Dependent variable (Y)** | **Total effect** | ***p _total_*** | **Effect of X on M** | ***p***  ***_X on M_*** | **Effect of M on Y** | ***p***  ***_M on Y_*** | **Direct effect** | ***p _direct_*** | **Mediation effect [95% CI]** | ***p***  ***_mediation_*** |
| **BL Alcohol Consumption** | **BL Neuroticism** | **BL Depression Scores** | 0.074 | 0.035 | -0.002 | 0.443 | 1.29 | 4.88E-49 | 0.077 | 0.015 | -0.003  [-∞, 0.032] | 0.458 |
| **FU1 Alcohol Consumption** | **FU1 Neuroticism** | **FU1 Depression Scores** | 0.057 | 0.011 | 0.003 | 0.362 | 1.44 | 1.48E-49 | 0.052 | 0.007 | 0.004  [-0.017, ∞] | 0.358 |
| **FU2 Alcohol Consumption** |  | **FU2 Depression Scores** | 0.014 | 0.319 |  |  |  |  |  |  |  |  |
| **FU3 Alcohol Consumption** |  | **FU3 Depression Scores** | -0.004 | 0.456 |  |  |  |  |  |  |  |  |
| **Females** | | | | | | | | | | | | |
| **Independent variable (X)** | **Mediation variable (M)** | **Dependent variable (Y)** | **Total effect** | ***p _total_*** | **Effect of X on M** | ***p***  ***_X on M_*** | **Effect of M on Y** | ***p***  ***_M on Y_*** | **Direct effect** | ***p _direct_*** | **Mediation effect [95% CI]** | ***p***  ***_mediation_*** |
| **BL Alcohol Consumption** | **BL Neuroticism** | **BL Depression Scores** | 0.230 | 7.47E-07 | 0.058 | 5.63E-05 | 1.61 | 1.08E-53 | 0.136 | 0.0005 | 0.093 [0.055, ∞] | **< 0.0001** |
| **FU1 Alcohol Consumption** | **FU1 Neuroticism** | **FU1 Depression Scores** | 0.239 | 1.2E-10 | 0.038 | 0.0003 | 2.03 | 1.12E-78 | 0.162 | 5.01E-08 | 0.077 [0.040, ∞] | **0.0002** |
| **FU2 Alcohol Consumption** | **FU2 Neuroticism** | **FU2 Depression Scores** | 0.186 | 1.46E-06 | 0.045 | 0.0004 | 1.88 | 6.54E-66 | 0.102 | 0.0005 | 0.084 [0.039, ∞] | **0.001** |
| **FU3 Alcohol Consumption** |  | **FU3 Depression Scores** | 0.006 | 0.445 |  |  |  |  |  |  |  |  |

| **The mediation effects between alcohol use problems and depression** | | | | | | | | | | | | |
| --- | --- | --- | --- | --- | --- | --- | --- | --- | --- | --- | --- | --- |
| **All people** | | | | | | | | | | | | |
| **Independent variable (X)** | **Mediation variable (M)** | **Dependent variable (Y)** | **Total effect** | ***p _total_*** | **Effect of X on M** | ***p***  ***_X on M_*** | **Effect of M on Y** | ***p***  ***_M on Y_*** | **Direct effect** | ***p _direct_*** | **Mediation effect [95% CI]** | ***p***  ***_mediation_*** |
| **BL Alcohol use problems** | **BL Neuroticism** | **BL Depression Scores** | 0.146 | 4.13E-06 | 0.046 | 1.82E-05 | 1.47 | 6.94E-101 | 0.078 | 0.003 | 0.068 [0.042, ∞] | **< 0.0001** |
| **FU1 Alcohol use problems** | **FU1 Neuroticism** | **FU1 Depression Scores** | 0.142 | 1.48E-09 | 0.036 | 7.77E-07 | 1.77 | 1.89E-125 | 0.079 | 4.41E-05 | 0.064 [0.039, ∞] | **< 0.0001** |
| **FU2 Alcohol use problems** | **FU2 Neuroticism** | **FU2 Depression Scores** | 0.089 | 3.61E-05 | 0.035 | 6.03E-06 | 1.77 | 2.12E-108 | 0.028 | 0.056 | 0.061 [0.034, ∞] | **0.0002** |
| **FU3 Alcohol use problems** | **FU3 Neuroticism** | **FU3 Depression Scores** | 0.103 | 4.3E-07 | 0.044 | 5.34E-08 | 1.53 | 2.02E-101 | 0.036 | 0.017 | 0.067 [0.044, ∞] | **< 0.0001** |
| **Males** | | | | | | | | | | | | |
| **Independent variable (X)** | **Mediation variable (M)** | **Dependent variable (Y)** | **Total effect** | ***p _total_*** | **Effect of X on M** | ***p***  ***_X on M_*** | **Effect of M on Y** | ***p***  ***_M on Y_*** | **Direct effect** | ***p _direct_*** | **Mediation effect [95% CI]** | ***p***  ***_mediation_*** |
| **BL Alcohol use problems** | **BL Neuroticism** | **BL Depression Scores** | 0.086 | 0.017 | 0.034 | 0.016 | 1.28 | 4.66E-48 | 0.043 | 0.112 | 0.043 [0.010, ∞] | **0.010** |
| **FU1 Alcohol use problems** | **FU1 Neuroticism** | **FU1 Depression Scores** | 0.089 | 0.002 | 0.029 | 0.003 | 1.43 | 3.25E-48 | 0.048 | 0.036 | 0.042 [0.015, ∞] | **0.004** |
| **FU2 Alcohol use problems** |  | **FU2 Depression Scores** | 0.028 | 0.166 |  |  |  |  |  |  |  |  |
| **FU3 Alcohol use problems** | **FU3 Neuroticism** | **FU3 Depression Scores** | 0.108 | 3.33E-06 | 0.042 | 6.42E-05 | 1.24 | 3.15E-38 | 0.057 | 0.002 | 0.051 [0.026, ∞] | **0.0002** |
| **Females** | | | | | | | | | | | | |
| **Independent variable (X)** | **Mediation variable (M)** | **Dependent variable (Y)** | **Total effect** | ***p _total_*** | **Effect of X on M** | ***p***  ***_X on M_*** | **Effect of M on Y** | ***p***  ***_M on Y_*** | **Direct effect** | ***p _direct_*** | **Mediation effect [95% CI]** | ***p***  ***_mediation_*** |
| **BL Alcohol use problems** | **BL Neuroticism** | **BL Depression Scores** | 0.208 | 2.68E-05 | 0.060 | 0.0001 | 1.62 | 3.57E-54 | 0.111 | 0.006 | 0.097 [0.056, ∞] | **< 0.0001** |
| **FU1 Alcohol use problems** | **FU1 Neuroticism** | **FU1 Depression Scores** | 0.199 | 3.48E-08 | 0.044 | 1.72E-05 | 2.04 | 8.98E-78 | 0.108 | 0.0001 | 0.090 [0.049, ∞] | **0.0001** |
| **FU2 Alcohol use problems** | **FU2 Neuroticism** | **FU2 Depression Scores** | 0.148 | 6.09E-06 | 0.054 | 1.17E-06 | 1.89 | 8.39E-65 | 0.047 | 0.039 | 0.101 [0.064, ∞] | **< 0.0001** |
| **FU3 Alcohol use problems** | **FU3 Neuroticism** | **FU3 Depression Scores** | 0.101 | 0.002 | 0.049 | 5.1E-05 | 1.75 | 8.58E-65 | 0.016 | 0.277 | 0.085 [0.045, ∞] | **0.0001** |

**Table S10. The fit measures for the cross-lagged panel model in the IMAGEN cohort**

| **Model Fit Measures of Depression and Alcohol Consumption** | | | | | | | | | |
| --- | --- | --- | --- | --- | --- | --- | --- | --- | --- |
| **Time** | **All** | | | **Male** | | | **Female** | | |
|  | **RMSEA** | **CFI** | **SRMR** | **RMSEA** | **CFI** | **SRMR** | **RMSEA** | **CFI** | **SRMR** |
| BL & FU1 | 0.078 | 0.979 | 0.017 | 0.075 | 0.976 | 0.018 | 0.086 | 0.972 | 0.022 |
| FU1 & FU2 | 0.085 | 0.976 | 0.017 | 0.087 | 0.969 | 0.018 | 0.075 | 0.979 | 0.018 |
| FU2 & FU3 | 0.075 | 0.982 | 0.015 | 0.080 | 0.976 | 0.018 | 0.075 | 0.980 | 0.017 |
| **Model Fit Measures of Depression and Alcohol Use Problems** | | | | | | | | | |
| **Time** | **All** | | | **Male** | | | **Female** | | |
|  | **RMSEA** | **CFI** | **SRMR** | **RMSEA** | **CFI** | **SRMR** | **RMSEA** | **CFI** | **SRMR** |
| BL & FU1 | 0.005 | 0.999 | 0.005 | 0.009 | 0.999 | 0.006 | 0.052 | 0.988 | 0.014 |
| FU1 & FU2 | 0.011 | 0.999 | 0.004 | 0.014 | 0.999 | 0.008 | 0.026 | 0.997 | 0.008 |
| FU2 & FU3 | 0.054 | 0.989 | 0.009 | 0.052 | 0.988 | 0.010 | 0.052 | 0.990 | 0.011 |

Model fit measures: root mean square error of approximation (RMSEA), comparative fit index (CFI), and standardized root mean square residual (SRMR)

**Table S11. The results of improved Mendelian randomisation analysis in the IMAGEN cohort**

| **The correlation between the PRSs and the corresponding phenotype.** | | | | | | | | | | | | |
| --- | --- | --- | --- | --- | --- | --- | --- | --- | --- | --- | --- | --- |
| **Threshold** | **PRSs of Depression** | | | | | | | | | | | |
|  | **Baseline**  **All** | | **Baseline**  **Male** | | **Baseline Female** | | **Follow-up 1 All** | | **Follow-up 1 Male** | | **Follow-up 1 Female** | |
|  | ***r*** | ***p _one-tailed_*** | ***r*** | ***p _one-tailed_*** | ***r*** | ***p _one-tailed_*** | ***r*** | ***p _one-tailed_*** | ***r*** | ***p _one-tailed_*** | ***r*** | ***p _one-tailed_*** |
| 0.05 | 0.05 | 0.0081 | 0.04 | 0.1024 | 0.06 | 0.0224 | 0.09 | 0.0001 | 0.11 | 0.0017 | 0.08 | 0.0090 |
| 0.10 | 0.05 | 0.0099 | 0.04 | 0.1290 | 0.06 | 0.0230 | 0.09 | 0.0001 | 0.11 | 0.0018 | 0.08 | 0.0095 |
| 0.15 | 0.05 | 0.0142 | 0.04 | 0.1360 | 0.06 | 0.0316 | 0.09 | 0.0002 | 0.11 | 0.0013 | 0.08 | 0.0114 |
| 0.20 | 0.05 | 0.0228 | 0.04 | 0.1410 | 0.05 | 0.0498 | 0.09 | 0.0002 | 0.11 | 0.0012 | 0.08 | 0.0160 |
| 0.25 | 0.04 | 0.0330 | 0.04 | 0.1411 | 0.05 | 0.0723 | 0.09 | 0.0003 | 0.11 | 0.0012 | 0.07 | 0.0225 |
| 0.30 | 0.04 | 0.0367 | 0.03 | 0.1557 | 0.05 | 0.0735 | 0.08 | 0.0005 | 0.11 | 0.0011 | 0.07 | 0.0333 |
| 0.35 | 0.04 | 0.0407 | 0.03 | 0.1500 | 0.04 | 0.0845 | 0.08 | 0.0008 | 0.11 | 0.0011 | 0.06 | 0.0489 |
| 0.40 | 0.04 | 0.0558 | 0.03 | 0.1588 | 0.04 | 0.1121 | 0.08 | 0.0016 | 0.11 | 0.0014 | 0.05 | 0.0702 |
| 0.45 | 0.03 | 0.0857 | 0.03 | 0.1846 | 0.03 | 0.1555 | 0.07 | 0.0026 | 0.11 | 0.0015 | 0.05 | 0.0965 |
| 0.50 | 0.03 | 0.1214 | 0.03 | 0.2169 | 0.03 | 0.1951 | 0.07 | 0.0036 | 0.10 | 0.0023 | 0.05 | 0.1025 |
| ***p_p_*_ermutation_** | **0.028** | | 0.142 | | 0.062 | | **0.000** | | **0.000** | | **0.017** | |
| **Threshold** | **PRSs of Depression** | | | | | | | | | | | |
|  | **Follow-up 2 All** | | **Follow-up 2 Male** | | **Follow-up 2 Female** | | **Follow-up 3 All** | | **Follow-up 3 Male** | | **Follow-up 3 Female** | |
|  | ***r*** | ***p _one-tailed_*** | ***r*** | ***p _one-tailed_*** | ***r*** | ***p _one-tailed_*** | ***r*** | ***p _one-tailed_*** | ***r*** | ***p _one-tailed_*** | ***r*** | ***p _one-tailed_*** |
| 0.05 | 0.08 | 0.0017 | 0.06 | 0.0923 | 0.10 | 0.0044 | 0.08 | 0.0032 | 0.05 | 0.1082 | 0.10 | 0.0063 |
| 0.10 | 0.08 | 0.0024 | 0.05 | 0.0980 | 0.10 | 0.0060 | 0.07 | 0.0058 | 0.04 | 0.1700 | 0.10 | 0.0070 |
| 0.15 | 0.08 | 0.0030 | 0.06 | 0.0855 | 0.09 | 0.0090 | 0.07 | 0.0078 | 0.04 | 0.1587 | 0.09 | 0.0107 |
| 0.20 | 0.08 | 0.0040 | 0.06 | 0.0756 | 0.09 | 0.0138 | 0.07 | 0.0091 | 0.05 | 0.1325 | 0.09 | 0.0164 |
| 0.25 | 0.07 | 0.0062 | 0.06 | 0.0744 | 0.08 | 0.0216 | 0.07 | 0.0113 | 0.05 | 0.1216 | 0.08 | 0.0236 |
| 0.30 | 0.06 | 0.0128 | 0.06 | 0.0851 | 0.07 | 0.0417 | 0.06 | 0.0161 | 0.05 | 0.1202 | 0.07 | 0.0359 |
| 0.35 | 0.06 | 0.0183 | 0.06 | 0.0779 | 0.06 | 0.0649 | 0.06 | 0.0188 | 0.06 | 0.0977 | 0.07 | 0.0517 |
| 0.40 | 0.06 | 0.0263 | 0.06 | 0.0719 | 0.05 | 0.0998 | 0.06 | 0.0281 | 0.06 | 0.0958 | 0.06 | 0.0807 |
| 0.45 | 0.05 | 0.0281 | 0.07 | 0.0512 | 0.04 | 0.1329 | 0.06 | 0.0294 | 0.06 | 0.0782 | 0.05 | 0.1000 |
| 0.50 | 0.05 | 0.0299 | 0.07 | 0.0406 | 0.04 | 0.1611 | 0.05 | 0.0342 | 0.06 | 0.0784 | 0.05 | 0.1168 |
| ***p_p_*_ermutation_** | **0.006** | | 0.077 | | **0.031** | | **0.013** | | 0.114 | | **0.029** | |
| **longitudinal *p_permutation_*** | **All: *p* < 0.001; Male: *p* < 0.001; Female: *p* < 0.001** | | | | | | | | | | | |

| **The correlation between the PRSs and the corresponding phenotype.** | | | | | | | | | | | | |
| --- | --- | --- | --- | --- | --- | --- | --- | --- | --- | --- | --- | --- |
| **Threshold** | **PRSs of Alcohol Consumption** | | | | | | | | | | | |
|  | **Baseline**  **All** | | **Baseline**  **Male** | | **Baseline Female** | | **Follow-up 1 All** | | **Follow-up 1 Male** | | **Follow-up 1 Female** | |
|  | ***r*** | ***p _one-tailed_*** | ***r*** | ***p _one-tailed_*** | ***r*** | ***p _one-tailed_*** | ***r*** | ***p _one-tailed_*** | ***r*** | ***p _one-tailed_*** | ***r*** | ***p _one-tailed_*** |
| 0.05 | 0.06 | 0.0026 | 0.08 | 0.0080 | 0.05 | 0.0545 | 0.06 | 0.0086 | 0.07 | 0.0289 | 0.05 | 0.0654 |
| 0.10 | 0.07 | 0.0017 | 0.08 | 0.0071 | 0.06 | 0.0394 | 0.06 | 0.0105 | 0.07 | 0.0299 | 0.05 | 0.0792 |
| 0.15 | 0.06 | 0.0030 | 0.08 | 0.0104 | 0.05 | 0.0515 | 0.06 | 0.0116 | 0.07 | 0.0307 | 0.05 | 0.0870 |
| 0.20 | 0.06 | 0.0057 | 0.07 | 0.0151 | 0.05 | 0.0729 | 0.06 | 0.0127 | 0.07 | 0.0281 | 0.05 | 0.1027 |
| 0.25 | 0.06 | 0.0055 | 0.07 | 0.0152 | 0.05 | 0.0750 | 0.06 | 0.0100 | 0.07 | 0.0276 | 0.05 | 0.0837 |
| 0.30 | 0.06 | 0.0047 | 0.07 | 0.0133 | 0.05 | 0.0731 | 0.06 | 0.0067 | 0.07 | 0.0264 | 0.06 | 0.0570 |
| 0.35 | 0.06 | 0.0041 | 0.08 | 0.0089 | 0.04 | 0.0869 | 0.06 | 0.0056 | 0.07 | 0.0253 | 0.06 | 0.0477 |
| 0.40 | 0.06 | 0.0038 | 0.08 | 0.0066 | 0.04 | 0.0952 | 0.06 | 0.0055 | 0.07 | 0.0244 | 0.06 | 0.0490 |
| 0.45 | 0.06 | 0.0053 | 0.08 | 0.0063 | 0.04 | 0.1302 | 0.06 | 0.0076 | 0.07 | 0.0284 | 0.06 | 0.0586 |
| 0.50 | 0.06 | 0.0057 | 0.08 | 0.0046 | 0.03 | 0.1629 | 0.06 | 0.0068 | 0.07 | 0.0259 | 0.06 | 0.0549 |
| ***p_p_*_ermutation_** | **0.003** | | **0.005** | | 0.062 | | **0.004** | | **0.020** | | 0.054 | |
| **Threshold** | **PRSs of Alcohol Consumption** | | | | | | | | | | | |
|  | **Follow-up 2 All** | | **Follow-up 2 Male** | | **Follow-up2 Female** | | **Follow-up 3 All** | | **Follow-up 3 Male** | | **Follow-up 3 Female** | |
|  | ***r*** | ***p _one-tailed_*** | ***r*** | ***p _one-tailed_*** | ***r*** | ***p _one-tailed_*** | ***r*** | ***p _one-tailed_*** | ***r*** | ***p _one-tailed_*** | ***r*** | ***p _one-tailed_*** |
| 0.05 | 0.09 | 0.0004 | 0.08 | 0.0240 | 0.11 | 0.0024 | 0.08 | 0.0026 | 0.07 | 0.0543 | 0.09 | 0.0084 |
| 0.10 | 0.08 | 0.0010 | 0.07 | 0.0361 | 0.10 | 0.0045 | 0.07 | 0.0066 | 0.06 | 0.0753 | 0.08 | 0.0168 |
| 0.15 | 0.08 | 0.0018 | 0.07 | 0.0392 | 0.09 | 0.0087 | 0.07 | 0.0093 | 0.06 | 0.0911 | 0.08 | 0.0206 |
| 0.20 | 0.08 | 0.0021 | 0.07 | 0.0393 | 0.09 | 0.0106 | 0.07 | 0.0119 | 0.05 | 0.1030 | 0.08 | 0.0246 |
| 0.25 | 0.08 | 0.0026 | 0.07 | 0.0467 | 0.09 | 0.0110 | 0.06 | 0.0160 | 0.05 | 0.1094 | 0.07 | 0.0344 |
| 0.30 | 0.08 | 0.0024 | 0.07 | 0.0487 | 0.09 | 0.0091 | 0.06 | 0.0134 | 0.05 | 0.1095 | 0.08 | 0.0278 |
| 0.35 | 0.08 | 0.0028 | 0.06 | 0.0594 | 0.09 | 0.0084 | 0.07 | 0.0102 | 0.06 | 0.0964 | 0.08 | 0.0235 |
| 0.40 | 0.08 | 0.0025 | 0.06 | 0.0623 | 0.09 | 0.0069 | 0.07 | 0.0100 | 0.06 | 0.0865 | 0.08 | 0.0263 |
| 0.45 | 0.07 | 0.0041 | 0.06 | 0.0810 | 0.09 | 0.0090 | 0.06 | 0.0133 | 0.06 | 0.0935 | 0.07 | 0.0343 |
| 0.50 | 0.07 | 0.0052 | 0.05 | 0.0964 | 0.09 | 0.0094 | 0.06 | 0.0138 | 0.06 | 0.0868 | 0.07 | 0.0401 |
| ***p_p_*_ermutation_** | **0.001** | | **0.047** | | **0.004** | | **0.005** | | 0.084 | | **0.015** | |
| **longitudinal *p_permutation_*** | **All: *p* < 0.001; Male: *p* < 0.001; Female: *p* < 0.001** | | | | | | | | | | | |

| **The statistical results of improved Mendelian randomisation analysis.** | | | | | | | | | | | | |
| --- | --- | --- | --- | --- | --- | --- | --- | --- | --- | --- | --- | --- |
| **Threshold** | **Valid-PRS_MDD_ → Alcohol Use Problems (BL All)** | | **Valid-PRS_MDD_ → Alcohol Use Problems (BL Male)** | | **Valid-PRS_MDD_→ Alcohol Use Problems**  **(BL Female)** | | **Valid-PRS_MDD_ → Alcohol Use Problems (FU1 All)** | | **Valid-PRS_MDD_ → Alcohol Use Problems**  **(FU1 Male)** | | **Valid-PRS_MDD_ → Alcohol Use Problems**  **(FU1 Female)** | |
|  | ***r*** | ***p _one-tailed_*** | ***r*** | ***p _one-tailed_*** | ***r*** | ***p _one-tailed_*** | ***r*** | ***p _one-tailed_*** | ***r*** | ***p _one-tailed_*** | ***r*** | ***p _one-tailed_*** |
| 0.05 | 0.04 | 0.0365 | 0.04 | 0.0863 | 0.03 | 0.1761 | 0.07 | 0.0025 | 0.10 | 0.0034 | 0.04 | 0.1274 |
| 0.10 | 0.04 | 0.0330 | 0.05 | 0.0829 | 0.03 | 0.1662 | 0.07 | 0.0024 | 0.10 | 0.0025 | 0.04 | 0.1448 |
| 0.15 | 0.04 | 0.0372 | 0.04 | 0.0846 | 0.03 | 0.1875 | 0.07 | 0.0025 | 0.11 | 0.0021 | 0.03 | 0.1661 |
| 0.20 | 0.04 | 0.0447 | 0.05 | 0.0816 | 0.02 | 0.2369 | 0.07 | 0.0035 | 0.10 | 0.0030 | 0.03 | 0.1837 |
| 0.25 | 0.04 | 0.0481 | 0.04 | 0.0892 | 0.02 | 0.2422 | 0.07 | 0.0051 | 0.10 | 0.0044 | 0.03 | 0.2002 |
| 0.30 | 0.04 | 0.0463 | 0.05 | 0.0781 | 0.02 | 0.2642 | 0.06 | 0.0074 | 0.09 | 0.0053 | 0.03 | 0.2368 |
| 0.35 | 0.04 | 0.0461 | 0.05 | 0.0746 | 0.02 | 0.2754 | 0.06 | 0.0133 | 0.09 | 0.0084 | 0.02 | 0.2870 |
| 0.40 | 0.04 | 0.0365 | 0.05 | 0.0676 | 0.02 | 0.2408 | 0.06 | 0.0140 | 0.09 | 0.0102 | 0.02 | 0.2727 |
| 0.45 | 0.04 | 0.0277 | 0.05 | 0.0538 | 0.02 | 0.2263 | 0.06 | 0.0122 | 0.08 | 0.0124 | 0.03 | 0.2249 |
| 0.50 | 0.05 | 0.0179 | 0.06 | 0.0413 | 0.03 | 0.1818 | 0.06 | 0.0092 | 0.08 | 0.0133 | 0.03 | 0.1740 |
| ***p_p_*_ermutation_** | **0.028** | | 0.061 | | 0.220 | | **0.002** | | **0.001** | | 0.179 | |
| **Threshold** | **Valid-PRS_MDD_ → Alcohol Use Problems (FU2 All)** | | **Valid-PRS_MDD_ → Alcohol Use Problem (FU2 Male)** | | **Valid-PRS_MDD_ → Alcohol Use Problems**  (**FU2 Female)** | | **Valid-PRS_MDD_ → Alcohol Use Problems (FU3 All)** | | **Valid-PRS_MDD_ → Alcohol Use Problems**  **(FU3 Male)** | | **Valid-PRS_MDD_ → Alcohol Use Problems**  **(FU3 Female)** | |
|  | ***r*** | ***p _one-tailed_*** | ***r*** | ***p _one-tailed_*** | ***r*** | ***p _one-tailed_*** | ***r*** | ***p _one-tailed_*** | ***r*** | ***p _one-tailed_*** | ***r*** | ***p _one-tailed_*** |
| 0.05 | 0.03 | 0.1626 | 0.06 | 0.0815 | -0.001 | 0.5140 | 0.05 | 0.0390 | 0.02 | 0.3335 | 0.09 | 0.0148 |
| 0.10 | 0.02 | 0.2071 | 0.05 | 0.1004 | -0.01 | 0.5597 | 0.05 | 0.0424 | 0.02 | 0.3393 | 0.08 | 0.0161 |
| 0.15 | 0.02 | 0.2143 | 0.05 | 0.0993 | -0.01 | 0.5849 | 0.05 | 0.0467 | 0.02 | 0.3197 | 0.08 | 0.0232 |
| 0.20 | 0.02 | 0.2253 | 0.05 | 0.0951 | -0.01 | 0.6219 | 0.05 | 0.0500 | 0.02 | 0.2940 | 0.07 | 0.0319 |
| 0.25 | 0.02 | 0.2373 | 0.05 | 0.0870 | -0.02 | 0.6689 | 0.05 | 0.0486 | 0.03 | 0.2744 | 0.07 | 0.0362 |
| 0.30 | 0.02 | 0.2523 | 0.06 | 0.0653 | -0.03 | 0.7522 | 0.05 | 0.0541 | 0.03 | 0.2671 | 0.07 | 0.0450 |
| 0.35 | 0.02 | 0.2642 | 0.06 | 0.0545 | -0.03 | 0.8021 | 0.05 | 0.0444 | 0.03 | 0.2242 | 0.07 | 0.0469 |
| 0.40 | 0.02 | 0.2595 | 0.06 | 0.0516 | -0.03 | 0.8063 | 0.05 | 0.0473 | 0.03 | 0.2071 | 0.06 | 0.0584 |
| 0.45 | 0.02 | 0.2660 | 0.06 | 0.0596 | -0.03 | 0.7919 | 0.05 | 0.0476 | 0.03 | 0.2143 | 0.06 | 0.0553 |
| 0.50 | 0.02 | 0.2656 | 0.06 | 0.0547 | -0.03 | 0.8015 | 0.05 | 0.0586 | 0.03 | 0.2373 | 0.06 | 0.0635 |
| ***p_p_*_ermutation_** | 0.209 | | 0.057 | | 0.687 | | **0.038** | | 0.281 | | **0.026** | |
| **longitudinal *p_permutation_*** | **All: *p* < 0.001; Male: *p* < 0.001; Female: *p* = 0.058** | | | | | | | | | | | |

| **The statistical results of improved Mendelian randomisation analysis.** | | | | | | | | | | | | |
| --- | --- | --- | --- | --- | --- | --- | --- | --- | --- | --- | --- | --- |
| **Threshold** | **Valid-PRS_alcohol_ →**  **Depression**  **(BL All)** | | **Valid-PRS_alcohol_ →**  **Depression (BL Male)** | | **Valid-PRS_alcohol_ →**  **Depression (BL Female)** | | **Valid-PRS_alcohol_ →**  **Depression (FU1 All)** | | **Valid-PRS_alcohol_ →**  **Depression (FU1 Male)** | | **Valid-PRS_alcohol_ →**  **Depression (FU1 Female)** | |
|  | ***r*** | ***p _one-tailed_*** | ***r*** | ***p _one-tailed_*** | ***r*** | ***p _one-tailed_*** | ***r*** | ***p _one-tailed_*** | ***r*** | ***p _one-tailed_*** | ***r*** | ***p _one-tailed_*** |
| 0.05 | 0.01 | 0.6570 | 0.03 | 0.8056 | -4.4E-04 | 0.4945 | -0.03 | 0.1178 | -0.04 | 0.1145 | -0.02 | 0.2736 |
| 0.10 | 0.01 | 0.7035 | 0.03 | 0.8214 | 0.004 | 0.5448 | -0.03 | 0.1127 | -0.04 | 0.1247 | -0.02 | 0.2526 |
| 0.15 | 0.01 | 0.7225 | 0.03 | 0.8432 | 0.004 | 0.5480 | -0.03 | 0.0917 | -0.04 | 0.1186 | -0.03 | 0.2156 |
| 0.20 | 0.01 | 0.6992 | 0.03 | 0.8141 | 0.003 | 0.5415 | -0.03 | 0.0912 | -0.05 | 0.0930 | -0.03 | 0.2422 |
| 0.25 | 0.01 | 0.6945 | 0.03 | 0.8243 | 0.001 | 0.5169 | -0.03 | 0.0933 | -0.05 | 0.0759 | -0.02 | 0.2679 |
| 0.30 | 0.01 | 0.7191 | 0.03 | 0.8298 | 0.003 | 0.5379 | -0.03 | 0.1064 | -0.05 | 0.0805 | -0.02 | 0.2861 |
| 0.35 | 0.02 | 0.7736 | 0.03 | 0.8222 | 0.01 | 0.6239 | -0.03 | 0.1232 | -0.05 | 0.0787 | -0.02 | 0.3234 |
| 0.40 | 0.02 | 0.8027 | 0.03 | 0.7932 | 0.02 | 0.6992 | -0.03 | 0.1402 | -0.05 | 0.0865 | -0.01 | 0.3466 |
| 0.45 | 0.02 | 0.8209 | 0.03 | 0.7999 | 0.02 | 0.7161 | -0.03 | 0.1280 | -0.05 | 0.1090 | -0.02 | 0.2922 |
| 0.50 | 0.02 | 0.8317 | 0.03 | 0.8181 | 0.02 | 0.7103 | -0.03 | 0.1459 | -0.04 | 0.1489 | -0.02 | 0.2856 |
| ***p_p_*_ermutation_** | 0.734 | | 0.829 | | 0.591 | | 0.108 | | 0.090 | | 0.277 | |
| **Threshold** | **Valid-PRS_alcohol_ →**  **Depression (FU2 All)** | | **Valid-PRS_alcohol_ →**  **Depression (FU2 Male)** | | **Valid-PRS_alcohol_ →**  **Depression (FU2 Female)** | | **Valid-PRS_alcohol_ →**  **Depression (FU3 All)** | | **Valid-PRS_alcohol_ →**  **Depression (FU3 Male)** | | **Valid-PRS_alcohol_ →**  **Depression (FU3 Female)** | |
|  | ***r*** | ***p _one-tailed_*** | ***r*** | ***p _one-tailed_*** | ***r*** | ***p _one-tailed_*** | ***r*** | ***p _one-tailed_*** | ***r*** | ***p _one-tailed_*** | ***r*** | ***p _one-tailed_*** |
| 0.05 | -0.01 | 0.4300 | 0.01 | 0.5869 | -0.01 | 0.3616 | -0.05 | 0.0587 | 0.01 | 0.6028 | -0.08 | 0.0190 |
| 0.10 | -0.004 | 0.4510 | 0.01 | 0.5837 | -0.01 | 0.3845 | -0.05 | 0.0401 | 0.01 | 0.5913 | -0.09 | 0.0108 |
| 0.15 | -0.01 | 0.3707 | 0.01 | 0.5733 | -0.02 | 0.3011 | -0.06 | 0.0268 | 0.01 | 0.6033 | -0.10 | 0.0056 |
| 0.20 | -0.01 | 0.3306 | 0.001 | 0.5081 | -0.02 | 0.2893 | -0.06 | 0.0271 | 0.01 | 0.6102 | -0.10 | 0.0054 |
| 0.25 | -0.02 | 0.2827 | -0.003 | 0.4721 | -0.03 | 0.2508 | -0.06 | 0.0210 | 0.01 | 0.5790 | -0.11 | 0.0043 |
| 0.30 | -0.02 | 0.2475 | -0.004 | 0.4581 | -0.03 | 0.2148 | -0.06 | 0.0226 | 0.01 | 0.5611 | -0.10 | 0.0052 |
| 0.35 | -0.02 | 0.2220 | -0.01 | 0.3975 | -0.03 | 0.2166 | -0.05 | 0.0325 | 0.003 | 0.5255 | -0.09 | 0.0105 |
| 0.40 | -0.03 | 0.1836 | -0.02 | 0.3256 | -0.03 | 0.2051 | -0.05 | 0.0346 | -9.9E-05 | 0.4991 | -0.09 | 0.0130 |
| 0.45 | -0.03 | 0.1258 | -0.03 | 0.2259 | -0.04 | 0.1781 | -0.05 | 0.0378 | 0.001 | 0.5135 | -0.09 | 0.0136 |
| 0.50 | -0.04 | 0.0877 | -0.04 | 0.1616 | -0.04 | 0.1570 | -0.05 | 0.0472 | 0.003 | 0.5247 | -0.09 | 0.0171 |
| ***p_p_*_ermutation_** | 0.262 | | 0.420 | | 0.263 | | **0.024** | | 0.536 | | **0.006** | |
| **longitudinal *p_permutation_*** | **All: *p* = 0.043; Male: *p* = 0.385; Female: *p* = 0.027** | | | | | | | | | | | |

**Table S12. The results of improved Mendelian randomisation analysis in the IMAGEN cohort after excluding 19 individuals who never initiated alcohol use across all four waves**

| **The correlation between the PRSs and the corresponding phenotype.** | | | | | | | | | | | | |
| --- | --- | --- | --- | --- | --- | --- | --- | --- | --- | --- | --- | --- |
| **Threshold** | **PRSs of Depression** | | | | | | | | | | | |
|  | **Baseline**  **All** | | **Baseline**  **Male** | | **Baseline Female** | | **Follow-up 1 All** | | **Follow-up 1 Male** | | **Follow-up 1 Female** | |
|  | ***r*** | ***p _one-tailed_*** | ***r*** | ***p _one-tailed_*** | ***r*** | ***p _one-tailed_*** | ***r*** | ***p _one-tailed_*** | ***r*** | ***p _one-tailed_*** | ***r*** | ***p _one-tailed_*** |
| 0.05 | 0.06 | 0.0073 | 0.04 | 0.1083 | 0.07 | 0.0191 | 0.10 | 0.0001 | 0.11 | 0.0017 | 0.09 | 0.0064 |
| 0.10 | 0.05 | 0.0086 | 0.04 | 0.1337 | 0.07 | 0.0192 | 0.10 | 0.0001 | 0.11 | 0.0018 | 0.09 | 0.0068 |
| 0.15 | 0.05 | 0.0125 | 0.04 | 0.1408 | 0.06 | 0.0266 | 0.10 | 0.0001 | 0.11 | 0.0013 | 0.09 | 0.0083 |
| 0.20 | 0.05 | 0.0202 | 0.03 | 0.1450 | 0.06 | 0.0429 | 0.09 | 0.0001 | 0.11 | 0.0012 | 0.08 | 0.0121 |
| 0.25 | 0.04 | 0.0301 | 0.03 | 0.1448 | 0.05 | 0.0644 | 0.09 | 0.0002 | 0.11 | 0.0011 | 0.08 | 0.0182 |
| 0.30 | 0.04 | 0.0338 | 0.03 | 0.1595 | 0.05 | 0.0661 | 0.09 | 0.0004 | 0.11 | 0.0011 | 0.07 | 0.0282 |
| 0.35 | 0.04 | 0.0379 | 0.03 | 0.1549 | 0.05 | 0.0764 | 0.08 | 0.0006 | 0.11 | 0.0010 | 0.06 | 0.0421 |
| 0.40 | 0.04 | 0.0518 | 0.03 | 0.1653 | 0.04 | 0.1001 | 0.08 | 0.0012 | 0.11 | 0.0013 | 0.06 | 0.0610 |
| 0.45 | 0.03 | 0.0795 | 0.03 | 0.1916 | 0.04 | 0.1392 | 0.07 | 0.0021 | 0.11 | 0.0015 | 0.05 | 0.0849 |
| 0.50 | 0.03 | 0.1120 | 0.03 | 0.2225 | 0.03 | 0.1747 | 0.07 | 0.0028 | 0.11 | 0.0021 | 0.05 | 0.0894 |
| ***p_p_*_ermutation_** | **0.021** | | 0.142 | | **0.046** | | **0.000** | | **0.002** | | **0.020** | |
| **Threshold** | **PRSs of Depression** | | | | | | | | | | | |
|  | **Follow-up 2 All** | | **Follow-up 2 Male** | | **Follow-up 2 Female** | | **Follow-up 3 All** | | **Follow-up 3 Male** | | **Follow-up 3 Female** | |
|  | ***r*** | ***p _one-tailed_*** | ***r*** | ***p _one-tailed_*** | ***r*** | ***p _one-tailed_*** | ***r*** | ***p _one-tailed_*** | ***r*** | ***p _one-tailed_*** | ***r*** | ***p _one-tailed_*** |
| 0.05 | 0.08 | 0.0015 | 0.05 | 0.0981 | 0.11 | 0.0035 | 0.08 | 0.0029 | 0.05 | 0.1119 | 0.10 | 0.0052 |
| 0.10 | 0.08 | 0.0021 | 0.05 | 0.1029 | 0.10 | 0.0050 | 0.08 | 0.0055 | 0.04 | 0.1752 | 0.10 | 0.0060 |
| 0.15 | 0.08 | 0.0028 | 0.06 | 0.0899 | 0.09 | 0.0077 | 0.07 | 0.0075 | 0.04 | 0.1627 | 0.10 | 0.0096 |
| 0.20 | 0.08 | 0.0037 | 0.06 | 0.0786 | 0.09 | 0.0120 | 0.07 | 0.0090 | 0.05 | 0.1347 | 0.09 | 0.0154 |
| 0.25 | 0.07 | 0.0054 | 0.06 | 0.0766 | 0.08 | 0.0184 | 0.07 | 0.0109 | 0.05 | 0.1227 | 0.08 | 0.0222 |
| 0.30 | 0.07 | 0.0112 | 0.06 | 0.0868 | 0.07 | 0.0358 | 0.06 | 0.0155 | 0.05 | 0.1200 | 0.07 | 0.0340 |
| 0.35 | 0.06 | 0.0163 | 0.06 | 0.0796 | 0.06 | 0.0572 | 0.06 | 0.0186 | 0.06 | 0.0967 | 0.07 | 0.0509 |
| 0.40 | 0.06 | 0.0243 | 0.06 | 0.0740 | 0.05 | 0.0907 | 0.06 | 0.0278 | 0.06 | 0.0944 | 0.06 | 0.0799 |
| 0.45 | 0.06 | 0.0263 | 0.07 | 0.0523 | 0.05 | 0.1239 | 0.06 | 0.0290 | 0.06 | 0.0768 | 0.05 | 0.0994 |
| 0.50 | 0.05 | 0.0281 | 0.07 | 0.0411 | 0.04 | 0.1525 | 0.05 | 0.0340 | 0.06 | 0.0765 | 0.05 | 0.1174 |
| ***p_p_*_ermutation_** | **0.009** | | 0.070 | | **0.018** | | **0.007** | | 0.090 | | **0.022** | |
| **longitudinal *p_permutation_*** | **All: *p* < 0.001; Male: *p* < 0.001; Female: *p* < 0.001** | | | | | | | | | | | |

| **The correlation between the PRSs and the corresponding phenotype.** | | | | | | | | | | | | |
| --- | --- | --- | --- | --- | --- | --- | --- | --- | --- | --- | --- | --- |
| **Threshold** | **PRSs of Alcohol Consumption** | | | | | | | | | | | |
|  | **Baseline**  **All** | | **Baseline**  **Male** | | **Baseline Female** | | **Follow-up 1 All** | | **Follow-up 1 Male** | | **Follow-up 1 Female** | |
|  | ***r*** | ***p _one-tailed_*** | ***r*** | ***p _one-tailed_*** | ***r*** | ***p _one-tailed_*** | ***r*** | ***p _one-tailed_*** | ***r*** | ***p _one-tailed_*** | ***r*** | ***p _one-tailed_*** |
| 0.05 | 0.06 | 0.0028 | 0.08 | 0.0077 | 0.05 | 0.0629 | 0.06 | 0.0098 | 0.07 | 0.0249 | 0.05 | 0.0866 |
| 0.10 | 0.07 | 0.0018 | 0.08 | 0.0067 | 0.05 | 0.0448 | 0.06 | 0.0113 | 0.07 | 0.0252 | 0.05 | 0.1002 |
| 0.15 | 0.06 | 0.0032 | 0.08 | 0.0096 | 0.05 | 0.0587 | 0.06 | 0.0121 | 0.07 | 0.0245 | 0.04 | 0.1111 |
| 0.20 | 0.06 | 0.0060 | 0.07 | 0.0140 | 0.04 | 0.0828 | 0.06 | 0.0134 | 0.07 | 0.0225 | 0.04 | 0.1309 |
| 0.25 | 0.06 | 0.0058 | 0.07 | 0.0140 | 0.04 | 0.0846 | 0.06 | 0.0103 | 0.08 | 0.0214 | 0.04 | 0.1064 |
| 0.30 | 0.06 | 0.0048 | 0.07 | 0.0122 | 0.05 | 0.0804 | 0.06 | 0.0065 | 0.08 | 0.0203 | 0.05 | 0.0699 |
| 0.35 | 0.06 | 0.0041 | 0.08 | 0.0080 | 0.04 | 0.0935 | 0.07 | 0.0051 | 0.08 | 0.0191 | 0.06 | 0.0565 |
| 0.40 | 0.06 | 0.0036 | 0.08 | 0.0060 | 0.04 | 0.0998 | 0.07 | 0.0048 | 0.08 | 0.0184 | 0.06 | 0.0549 |
| 0.45 | 0.06 | 0.0051 | 0.08 | 0.0057 | 0.04 | 0.1338 | 0.06 | 0.0065 | 0.07 | 0.0224 | 0.05 | 0.0627 |
| 0.50 | 0.06 | 0.0055 | 0.09 | 0.0043 | 0.03 | 0.1664 | 0.06 | 0.0058 | 0.08 | 0.0208 | 0.06 | 0.0577 |
| ***p_p_*_ermutation_** | **0.003** | | **0.002** | | 0.074 | | **0.000** | | **0.024** | | 0.076 | |
| **Threshold** | **PRSs of Alcohol Consumption** | | | | | | | | | | | |
|  | **Follow-up 2 All** | | **Follow-up 2 Male** | | **Follow-up 2 Female** | | **Follow-up 3 All** | | **Follow-up 3 Male** | | **Follow-up 3 Female** | |
|  | ***r*** | ***p _one-tailed_*** | ***r*** | ***p _one-tailed_*** | ***r*** | ***p _one-tailed_*** | ***r*** | ***p _one-tailed_*** | ***r*** | ***p _one-tailed_*** | ***r*** | ***p _one-tailed_*** |
| 0.05 | 0.09 | 0.0006 | 0.08 | 0.0192 | 0.10 | 0.0052 | 0.08 | 0.0041 | 0.07 | 0.0418 | 0.08 | 0.0202 |
| 0.10 | 0.08 | 0.0013 | 0.08 | 0.0289 | 0.09 | 0.0090 | 0.07 | 0.0092 | 0.07 | 0.0576 | 0.07 | 0.0357 |
| 0.15 | 0.08 | 0.0023 | 0.08 | 0.0296 | 0.08 | 0.0169 | 0.07 | 0.0124 | 0.06 | 0.0662 | 0.07 | 0.0434 |
| 0.20 | 0.08 | 0.0027 | 0.08 | 0.0299 | 0.08 | 0.0206 | 0.06 | 0.0159 | 0.06 | 0.0761 | 0.06 | 0.0514 |
| 0.25 | 0.07 | 0.0033 | 0.07 | 0.0349 | 0.08 | 0.0209 | 0.06 | 0.0204 | 0.06 | 0.0789 | 0.06 | 0.0679 |
| 0.30 | 0.08 | 0.0027 | 0.07 | 0.0363 | 0.08 | 0.0159 | 0.06 | 0.0158 | 0.06 | 0.0786 | 0.07 | 0.0509 |
| 0.35 | 0.08 | 0.0030 | 0.07 | 0.0441 | 0.08 | 0.0139 | 0.07 | 0.0112 | 0.06 | 0.0671 | 0.07 | 0.0406 |
| 0.40 | 0.08 | 0.0025 | 0.07 | 0.0467 | 0.09 | 0.0107 | 0.07 | 0.0104 | 0.07 | 0.0601 | 0.07 | 0.0418 |
| 0.45 | 0.07 | 0.0042 | 0.06 | 0.0643 | 0.08 | 0.0128 | 0.06 | 0.0135 | 0.06 | 0.0688 | 0.07 | 0.0494 |
| 0.50 | 0.07 | 0.0052 | 0.06 | 0.0792 | 0.08 | 0.0127 | 0.06 | 0.0136 | 0.06 | 0.0652 | 0.06 | 0.0545 |
| ***p_p_*_ermutation_** | **0.002** | | **0.028** | | **0.006** | | **0.011** | | **0.048** | | **0.037** | |
| **longitudinal *p_permutation_*** | **All: *p* < 0.001; Male: *p* < 0.001; Female: *p* < 0.001** | | | | | | | | | | | |

| **The statistical results of improved Mendelian randomisation analysis.** | | | | | | | | | | | | | |
| --- | --- | --- | --- | --- | --- | --- | --- | --- | --- | --- | --- | --- | --- |
| **Threshold** | **Valid-PRS_MDD_ → Alcohol Use Problems (BL All)** | | **Valid-PRS_MDD_ → Alcohol Use Problems (BL Male)** | | **Valid-PRS_MDD_→ Alcohol Use Problems**  **(BL Female)** | | **Valid-PRS_MDD_ → Alcohol Use Problems (FU1 All)** | | **Valid-PRS_MDD_ → Alcohol Use Problems**  **(FU1 Male)** | | | **Valid-PRS_MDD_ → Alcohol Use Problems**  **(FU1 Female)** | |
|  | ***r*** | ***p _one-tailed_*** | ***r*** | ***p _one-tailed_*** | ***r*** | ***p _one-tailed_*** | ***r*** | ***p _one-tailed_*** | ***r*** | ***p _one-tailed_*** | | ***r*** | ***p _one-tailed_*** |
| 0.05 | 0.04 | 0.0387 | 0.04 | 0.0881 | 0.03 | 0.1827 | 0.07 | 0.0027 | 0.10 | | 0.0038 | 0.04 | 0.1248 |
| 0.10 | 0.04 | 0.0348 | 0.05 | 0.0845 | 0.03 | 0.1717 | 0.07 | 0.0025 | 0.10 | | 0.0027 | 0.04 | 0.1410 |
| 0.15 | 0.04 | 0.0391 | 0.04 | 0.0861 | 0.03 | 0.1933 | 0.07 | 0.0026 | 0.10 | | 0.0023 | 0.04 | 0.1621 |
| 0.20 | 0.04 | 0.0471 | 0.05 | 0.0831 | 0.02 | 0.2444 | 0.07 | 0.0037 | 0.10 | | 0.0032 | 0.03 | 0.1813 |
| 0.25 | 0.04 | 0.0508 | 0.04 | 0.0909 | 0.02 | 0.2507 | 0.07 | 0.0054 | 0.10 | | 0.0048 | 0.03 | 0.1995 |
| 0.30 | 0.04 | 0.0492 | 0.05 | 0.0797 | 0.02 | 0.2735 | 0.06 | 0.0079 | 0.09 | | 0.0058 | 0.03 | 0.2372 |
| 0.35 | 0.04 | 0.0491 | 0.05 | 0.0763 | 0.02 | 0.2851 | 0.06 | 0.0143 | 0.09 | | 0.0091 | 0.02 | 0.2885 |
| 0.40 | 0.04 | 0.0388 | 0.05 | 0.0690 | 0.02 | 0.2495 | 0.06 | 0.0149 | 0.08 | | 0.0109 | 0.02 | 0.2741 |
| 0.45 | 0.04 | 0.0297 | 0.05 | 0.0551 | 0.02 | 0.2353 | 0.06 | 0.0133 | 0.08 | | 0.0134 | 0.03 | 0.2280 |
| 0.50 | 0.05 | 0.0193 | 0.06 | 0.0425 | 0.03 | 0.1887 | 0.06 | 0.0100 | 0.08 | | 0.0144 | 0.03 | 0.1754 |
| ***p_p_*_ermutation_** | **0.032** | | 0.062 | | 0.214 | | **0.007** | | **0.003** | | | 0.192 | |
| **Threshold** | **Valid-PRS_MDD_ → Alcohol Use Problems (FU2 All)** | | **Valid-PRS_MDD_ → Alcohol Use Problem (FU2 Male)** | | **Valid-PRS_MDD_ → Alcohol Use Problems** (**FU2 Female)** | | **Valid-PRS_MDD_ → Alcohol Use Problems (FU3 All)** | | **Valid-PRS_MDD_ → Alcohol Use Problems (FU3 Male)** | | | **Valid-PRS_MDD_ → Alcohol Use Problems (FU3 Female)** | |
|  | ***r*** | ***p _one-tailed_*** | ***r*** | ***p _one-tailed_*** | ***r*** | ***p _one-tailed_*** | ***r*** | ***p _one-tailed_*** | ***r*** | | ***p _one-tailed_*** | ***r*** | ***p _one-tailed_*** |
| 0.05 | 0.03 | 0.1646 | 0.05 | 0.0871 | 7.3E-05 | 0.4992 | 0.05 | 0.0413 | 0.02 | | 0.3506 | 0.09 | 0.0133 |
| 0.10 | 0.02 | 0.2072 | 0.05 | 0.1056 | -0.004 | 0.5436 | 0.05 | 0.0439 | 0.02 | | 0.3532 | 0.09 | 0.0143 |
| 0.15 | 0.02 | 0.2135 | 0.05 | 0.1036 | -0.01 | 0.5700 | 0.05 | 0.0479 | 0.02 | | 0.3310 | 0.08 | 0.0209 |
| 0.20 | 0.02 | 0.2260 | 0.05 | 0.0989 | -0.01 | 0.6113 | 0.05 | 0.0519 | 0.02 | | 0.3046 | 0.08 | 0.0297 |
| 0.25 | 0.02 | 0.2396 | 0.05 | 0.0904 | -0.02 | 0.6626 | 0.05 | 0.0511 | 0.02 | | 0.2843 | 0.07 | 0.0346 |
| 0.30 | 0.02 | 0.2564 | 0.06 | 0.0683 | -0.03 | 0.7489 | 0.05 | 0.0575 | 0.03 | | 0.2777 | 0.07 | 0.0439 |
| 0.35 | 0.02 | 0.2703 | 0.06 | 0.0575 | -0.03 | 0.8010 | 0.05 | 0.0480 | 0.03 | | 0.2351 | 0.07 | 0.0462 |
| 0.40 | 0.02 | 0.2650 | 0.06 | 0.0541 | -0.03 | 0.8056 | 0.05 | 0.0506 | 0.03 | | 0.2161 | 0.06 | 0.0575 |
| 0.45 | 0.02 | 0.2745 | 0.06 | 0.0629 | -0.03 | 0.7938 | 0.05 | 0.0520 | 0.03 | | 0.2250 | 0.06 | 0.0555 |
| 0.50 | 0.02 | 0.2733 | 0.06 | 0.0579 | -0.03 | 0.8017 | 0.04 | 0.0634 | 0.03 | | 0.2496 | 0.06 | 0.0628 |
| ***p_p_*_ermutation_** | 0.232 | | 0.067 | | 0.697 | | **0.047** | | 0.300 | | | **0.029** | |
| **longitudinal *p_permutation_*** | **All: *p* < 0.001; Male: *p* < 0.001; Female: *p* = 0.063** | | | | | | | | | | | | |

| **The statistical results of improved Mendelian randomisation analysis.** | | | | | | | | | | | | |
| --- | --- | --- | --- | --- | --- | --- | --- | --- | --- | --- | --- | --- |
| **Threshold** | **Valid-PRS_alcohol_ →**  **Depression**  **(BL All)** | | **Valid-PRS_alcohol_ →**  **Depression (BL Male)** | | **Valid-PRS_alcohol_ →**  **Depression (BL Female)** | | **Valid-PRS_alcohol_ →**  **Depression (FU1 All)** | | **Valid-PRS_alcohol_ →**  **Depression (FU1 Male)** | | **Valid-PRS_alcohol_ →**  **Depression (FU1 Female)** | |
|  | ***r*** | ***p _one-tailed_*** | ***r*** | ***p _one-tailed_*** | ***r*** | ***p _one-tailed_*** | ***r*** | ***p _one-tailed_*** | ***r*** | ***p _one-tailed_*** | ***r*** | ***p _one-tailed_*** |
| 0.05 | 0.01 | 0.6168 | 0.03 | 0.8146 | -0.006 | 0.4303 | -0.04 | 0.0868 | -0.04 | 0.1239 | -0.03 | 0.2027 |
| 0.10 | 0.01 | 0.6638 | 0.03 | 0.8290 | -0.002 | 0.4792 | -0.04 | 0.0810 | -0.04 | 0.1333 | -0.03 | 0.1827 |
| 0.15 | 0.01 | 0.6835 | 0.03 | 0.8500 | -0.002 | 0.4811 | -0.04 | 0.0632 | -0.04 | 0.1252 | -0.04 | 0.1500 |
| 0.20 | 0.01 | 0.6571 | 0.03 | 0.8206 | -0.002 | 0.4733 | -0.04 | 0.0621 | -0.05 | 0.0995 | -0.03 | 0.1691 |
| 0.25 | 0.01 | 0.6532 | 0.03 | 0.8322 | -0.004 | 0.4483 | -0.04 | 0.0637 | -0.05 | 0.0820 | -0.03 | 0.1903 |
| 0.30 | 0.01 | 0.6816 | 0.03 | 0.8376 | -0.002 | 0.4733 | -0.04 | 0.0752 | -0.05 | 0.0864 | -0.03 | 0.2103 |
| 0.35 | 0.01 | 0.7383 | 0.03 | 0.8289 | 0.005 | 0.5606 | -0.04 | 0.0867 | -0.05 | 0.0825 | -0.03 | 0.2417 |
| 0.40 | 0.02 | 0.7743 | 0.03 | 0.8021 | 0.012 | 0.6469 | -0.03 | 0.1004 | -0.05 | 0.0902 | -0.02 | 0.2638 |
| 0.45 | 0.02 | 0.7997 | 0.03 | 0.8113 | 0.014 | 0.6723 | -0.03 | 0.0928 | -0.04 | 0.1137 | -0.03 | 0.2201 |
| 0.50 | 0.02 | 0.8166 | 0.03 | 0.8293 | 0.015 | 0.6743 | -0.03 | 0.1114 | -0.04 | 0.1550 | -0.03 | 0.2210 |
| ***p_p_*_ermutation_** | 0.691 | | 0.848 | | 0.546 | | 0.075 | | 0.096 | | 0.195 | |
| **Threshold** | **Valid-PRS_alcohol_ →**  **Depression (FU2 All)** | | **Valid-PRS_alcohol_ →**  **Depression (FU2 Male)** | | **Valid-PRS_alcohol_ →**  **Depression (FU2 Female)** | | **Valid-PRS_alcohol_ →**  **Depression (FU3 All)** | | **Valid-PRS_alcohol_ →**  **Depression (FU3 Male)** | | **Valid-PRS_alcohol_ →**  **Depression (FU3 Female)** | |
|  | ***r*** | ***p _one-tailed_*** | ***r*** | ***p _one-tailed_*** | ***r*** | ***p _one-tailed_*** | ***r*** | ***p _one-tailed_*** | ***r*** | ***p _one-tailed_*** | ***r*** | ***p _one-tailed_*** |
| 0.05 | 0.001 | 0.5179 | 0.009 | 0.5818 | -0.003 | 0.4717 | -0.04 | 0.0895 | 0.01 | 0.6036 | -0.07 | 0.0358 |
| 0.10 | 0.003 | 0.5371 | 0.008 | 0.5794 | -6.1E-04 | 0.4938 | -0.05 | 0.0620 | 0.01 | 0.5918 | -0.08 | 0.0209 |
| 0.15 | -0.003 | 0.4525 | 0.008 | 0.5707 | -0.01 | 0.3996 | -0.05 | 0.0418 | 0.01 | 0.6044 | -0.09 | 0.0110 |
| 0.20 | -0.007 | 0.4065 | 0.001 | 0.5055 | -0.01 | 0.3838 | -0.05 | 0.0420 | 0.01 | 0.6162 | -0.09 | 0.0103 |
| 0.25 | -0.01 | 0.3538 | -0.003 | 0.4693 | -0.02 | 0.3397 | -0.05 | 0.0327 | 0.009 | 0.5838 | -0.10 | 0.0082 |
| 0.30 | -0.01 | 0.3140 | -0.005 | 0.4551 | -0.02 | 0.2969 | -0.05 | 0.0346 | 0.007 | 0.5656 | -0.09 | 0.0097 |
| 0.35 | -0.02 | 0.2813 | -0.01 | 0.3939 | -0.02 | 0.2949 | -0.05 | 0.0470 | 0.003 | 0.5269 | -0.09 | 0.0182 |
| 0.40 | -0.02 | 0.2389 | -0.02 | 0.3248 | -0.02 | 0.2820 | -0.05 | 0.0500 | 0.000 | 0.5019 | -0.08 | 0.0221 |
| 0.45 | -0.03 | 0.1694 | -0.03 | 0.2277 | -0.03 | 0.2478 | -0.05 | 0.0549 | 0.002 | 0.5154 | -0.08 | 0.0234 |
| 0.50 | -0.03 | 0.1195 | -0.04 | 0.1644 | -0.03 | 0.2177 | -0.04 | 0.0686 | 0.003 | 0.5283 | -0.08 | 0.0295 |
| ***p_p_*_ermutation_** | 0.314 | | 0.397 | | 0.339 | | **0.042** | | 0.544 | | **0.017** | |
| **longitudinal *p_permutation_*** | **All: *p* = 0.041; Male: *p* = 0.410; Female: *p* = 0.021** | | | | | | | | | | | |

**Table S13. The correlation between alcohol consumption/alcohol use problems and depression in the HCP cohort**

| **Correlation between Depression Scores and Alcohol Use Behaviours** | | | | | |
| --- | --- | --- | --- | --- | --- |
| **Controlled for sex and age** | | | | | |
| **Depression Scores and**  **Alcohol Consumption** | ***r***  **[95% CI]** | ***p*** | **Depression Scores and**  **Alcohol Use Problems** | ***r***  **[95% CI]** | ***p*** |
| **All** | -0.061  [-0.144, 0.022] | 0.160 | **All** | 0.207  [0.127, 0.293] | **8.28E-07** |
| **Male** | -0.087  [-0.201, 0.026] | 0.139 | **Male** | 0.195  [0.084, 0.312] | **6.77E-04** |
| **Female** | -0.036  [-0.158, 0.086] | 0.574 | **Female** | 0.221  [0.103, 0.347] | **3.35E-04** |
| **Controlled for sex, age, and the other alcohol use behaviour** | | | | | |
| **Depression Scores and Alcohol Consumption (Controlled for Alcohol Use Problems)** | ***r***  **[95% CI]** | ***p*** | **Depression Scores and Alcohol Use Problems (Controlled for Alcohol Consumption)** | ***r***  **[95% CI]** | ***p*** |
| **All** | -0.131  [-0.215, -0.049] | **0.002** | **All** | 0.235  [0.157, 0.323] | **3.40E-08** |
| **Male** | -0.158  [-0.273, -0.045] | **0.007** | **Male** | 0.237  [0.127, 0.355] | **4.97E-05** |
| **Female** | -0.100  [-0.223, 0.021] | 0.115 | **Female** | 0.232  [0.114, 0.358] | **2.27E-04** |

**Table S14. The results of improved Mendelian Randomisation analysis in the HCP cohort** **with adjustment for age and sex**

| **The statistical results of improved Mendelian Randomisation analysis.** | | | | | | | | | | | | |
| --- | --- | --- | --- | --- | --- | --- | --- | --- | --- | --- | --- | --- |
| **Threshold** | **Valid-PRS_MDD_ → Alcohol** **Use Problems (All)** | | **Valid-PRS_MDD_ → Alcohol Use Problems (Male)** | | **Valid-PRS_MDD_ → Alcohol Use Problems (Female)** | | **Valid-PRS_alcohol_ →**  **Depression (ASR DSM) (All)** | | **Valid-PRS_alcohol_ →**  **Depression (ASR DSM) (Male)** | | **Valid-PRS_alcohol_ →**  **Depression (ASR DSM) (Female)** | |
|  | ***r*** | ***p _one-tailed_*** | ***r*** | ***p _one-tailed_*** | ***r*** | ***p _one-tailed_*** | ***r*** | ***p _one-tailed_*** | ***r*** | ***p _one-tailed_*** | ***r*** | ***p _one-tailed_*** |
| 0.05 | 0.11 | 0.0478 | 0.11 | 0.1258 | 0.12 | 0.1152 | -0.06 | 0.1916 | 0.02 | 0.5827 | -0.18 | 0.0382 |
| 0.10 | 0.11 | 0.0495 | 0.11 | 0.1207 | 0.11 | 0.1353 | -0.04 | 0.2643 | 0.03 | 0.6380 | -0.17 | 0.0536 |
| 0.15 | 0.12 | 0.0410 | 0.11 | 0.1099 | 0.12 | 0.1248 | -0.05 | 0.2467 | 0.03 | 0.6428 | -0.18 | 0.0415 |
| 0.20 | 0.12 | 0.0388 | 0.12 | 0.0988 | 0.11 | 0.1350 | -0.04 | 0.2713 | 0.04 | 0.6501 | -0.16 | 0.0552 |
| 0.25 | 0.12 | 0.0380 | 0.12 | 0.0965 | 0.11 | 0.1384 | -0.04 | 0.2809 | 0.04 | 0.6633 | -0.16 | 0.0596 |
| 0.30 | 0.11 | 0.0501 | 0.11 | 0.1168 | 0.11 | 0.1460 | -0.05 | 0.2555 | 0.03 | 0.6383 | -0.16 | 0.0598 |
| 0.35 | 0.11 | 0.0588 | 0.10 | 0.1323 | 0.11 | 0.1485 | -0.05 | 0.2496 | 0.03 | 0.6064 | -0.15 | 0.0701 |
| 0.40 | 0.10 | 0.0831 | 0.09 | 0.1734 | 0.11 | 0.1472 | -0.06 | 0.2088 | 0.02 | 0.5748 | -0.17 | 0.0531 |
| 0.45 | 0.10 | 0.0724 | 0.10 | 0.1389 | 0.10 | 0.1650 | -0.05 | 0.2338 | 0.03 | 0.6084 | -0.16 | 0.0580 |
| 0.50 | 0.11 | 0.0542 | 0.12 | 0.1027 | 0.10 | 0.1675 | -0.06 | 0.2118 | 0.02 | 0.5795 | -0.16 | 0.0579 |
| ***p_p_*_ermutation_** | **0.041** | | 0.103 | | 0.117 | | 0.234 | | 0.592 | | **0.047** | |

| **The statistical results of improved Mendelian Randomisation analysis between valid-PRS_alcohol_ and the number of endorsed depressive symptoms meeting DSM-IV criteria for major depression over an individual’s lifetime** | | | | | | |
| --- | --- | --- | --- | --- | --- | --- |
| **Threshold** | **Valid-PRS_alcohol_ →**  **Depression (DSM-IV)**  **(All)** | | **Valid-PRS_alcohol_ →**  **Depression (DSM-IV)**  **(Male)** | | **Valid-PRS_alcohol_ →**  **Depression (DSM-IV) (Female)** | |
|  | ***r*** | ***p _one-tailed_*** | ***r*** | ***p _one-tailed_*** | ***r*** | ***p _one-tailed_*** |
| 0.05 | -0.02 | 0.3768 | 0.12 | 0.8968 | -0.20 | 0.0279 |
| 0.10 | -0.02 | 0.3890 | 0.12 | 0.9015 | -0.21 | 0.0239 |
| 0.15 | -0.03 | 0.3207 | 0.12 | 0.8905 | -0.23 | 0.0140 |
| 0.20 | -0.04 | 0.2703 | 0.11 | 0.8858 | -0.25 | 0.0093 |
| 0.25 | -0.06 | 0.2056 | 0.11 | 0.8880 | -0.27 | 0.0043 |
| 0.30 | -7.13E-02 | 0.1550 | 0.11 | 0.8718 | -0.29 | 0.0030 |
| 0.35 | -6.89E-02 | 0.1631 | 0.10 | 0.8604 | -0.27 | 0.0043 |
| 0.40 | -0.07 | 0.1454 | 0.09 | 0.8393 | -0.28 | 0.0036 |
| 0.45 | -0.07 | 0.1555 | 0.10 | 0.8493 | -0.27 | 0.0043 |
| 0.50 | -0.08 | 0.1334 | 0.09 | 0.8406 | -0.28 | 0.0035 |
| ***p_p_*_ermutation_** | 0.223 | | 0.903 | | **0.002** | |

**Table S15. The results of improved Mendelian Randomisation analysis in the HCP cohort with adjustment for age, sex, and top 10 principal components**

| **The statistical results of improved Mendelian Randomisation analysis.** | | | | | | | | | | | | |
| --- | --- | --- | --- | --- | --- | --- | --- | --- | --- | --- | --- | --- |
| **Threshold** | **Valid-PRS_MDD_ → Alcohol** **Use Problems (All)** | | **Valid-PRS_MDD_ → Alcohol Use Problems (Male)** | | **Valid-PRS_MDD_ → Alcohol Use Problems (Female)** | | **Valid-PRS_alcohol_ →**  **Depression (ASR DSM) (All)** | | **Valid-PRS_alcohol_ →**  **Depression (ASR DSM) (Male)** | | **Valid-PRS_alcohol_ →**  **Depression (ASR DSM) (Female)** | |
|  | ***r*** | ***p _one-tailed_*** | ***r*** | ***p _one-tailed_*** | ***r*** | ***p _one-tailed_*** | ***r*** | ***p _one-tailed_*** | ***r*** | ***p _one-tailed_*** | ***r*** | ***p _one-tailed_*** |
| 0.05 | 0.11 | 0.0672 | 0.10 | 0.1638 | 0.12 | 0.1152 | 0.04 | 0.7282 | 0.10 | 0.8524 | -0.07 | 0.2520 |
| 0.10 | 0.11 | 0.0664 | 0.10 | 0.1470 | 0.11 | 0.1353 | 0.06 | 0.7843 | 0.12 | 0.8859 | -0.06 | 0.2903 |
| 0.15 | 0.11 | 0.0541 | 0.11 | 0.1311 | 0.12 | 0.1248 | 0.05 | 0.7631 | 0.12 | 0.8859 | -0.08 | 0.2457 |
| 0.20 | 0.11 | 0.0518 | 0.11 | 0.1209 | 0.11 | 0.1350 | 0.05 | 0.7782 | 0.12 | 0.8871 | -0.07 | 0.2699 |
| 0.25 | 0.12 | 0.0499 | 0.11 | 0.1221 | 0.11 | 0.1384 | 0.05 | 0.7784 | 0.12 | 0.8983 | -0.07 | 0.2498 |
| 0.30 | 0.11 | 0.0616 | 0.11 | 0.1356 | 0.11 | 0.1460 | 0.05 | 0.7510 | 0.11 | 0.8775 | -0.07 | 0.2484 |
| 0.35 | 0.10 | 0.0701 | 0.10 | 0.1421 | 0.11 | 0.1485 | 0.04 | 0.7193 | 0.10 | 0.8489 | -0.07 | 0.2486 |
| 0.40 | 0.09 | 0.0980 | 0.09 | 0.1836 | 0.11 | 0.1472 | 0.03 | 0.6551 | 0.09 | 0.8211 | -0.10 | 0.1910 |
| 0.45 | 0.10 | 0.0830 | 0.10 | 0.1487 | 0.10 | 0.1650 | 0.02 | 0.6322 | 0.09 | 0.8359 | -0.11 | 0.1606 |
| 0.50 | 0.11 | 0.0626 | 0.12 | 0.1168 | 0.10 | 0.1675 | 0.01 | 0.5693 | 0.08 | 0.8060 | -0.12 | 0.1312 |
| ***p_p_*_ermutation_** | **0.049** | | 0.144 | | 0.189 | | 0.744 | | 0.859 | | 0.233 | |

| **The statistical results of improved Mendelian Randomisation analysis between valid-PRS_alcohol_ and the number of endorsed depressive symptoms meeting DSM-IV criteria for major depression over an individual’s lifetime** | | | | | | |
| --- | --- | --- | --- | --- | --- | --- |
| **Threshold** | **Valid-PRS_alcohol_ →**  **Depression (DSM-IV)**  **(All)** | | **Valid-PRS_alcohol_ →**  **Depression (DSM-IV)**  **(Male)** | | **Valid-PRS_alcohol_ →**  **Depression (DSM-IV) (Female)** | |
|  | ***r*** | ***p _one-tailed_*** | ***r*** | ***p _one-tailed_*** | ***r*** | ***p _one-tailed_*** |
| 0.05 | 0.06 | 0.8045 | 0.23 | 0.9900 | -0.13 | 0.1173 |
| 0.10 | 0.06 | 0.7957 | 0.23 | 0.9903 | -0.14 | 0.1017 |
| 0.15 | 0.04 | 0.7322 | 0.22 | 0.9889 | -0.17 | 0.0636 |
| 0.20 | 0.03 | 0.6685 | 0.22 | 0.9881 | -0.20 | 0.0385 |
| 0.25 | 0.01 | 0.5715 | 0.23 | 0.9900 | -0.23 | 0.0178 |
| 0.30 | -2.04E-03 | 0.4887 | 0.22 | 0.9868 | -0.25 | 0.0125 |
| 0.35 | -2.44E-03 | 0.4865 | 0.21 | 0.9826 | -0.24 | 0.0154 |
| 0.40 | -0.01 | 0.4527 | 0.20 | 0.9764 | -0.25 | 0.0122 |
| 0.45 | -0.01 | 0.4344 | 0.19 | 0.9753 | -0.25 | 0.0121 |
| 0.50 | -0.02 | 0.3742 | 0.18 | 0.9694 | -0.26 | 0.0088 |
| ***p_p_*_ermutation_** | 0.586 | | 0.988 | | **0.021** | |

**Table S16. The mediation effects between valid-PRSs and alcohol/depression in the IMAGEN cohort.** The *p*-values of the mediation effects were obtained with 10,000 times bootstrap samplings. All *p*-values and confidence intervals are reported as one-tailed.

| **All people** | | | | | | | | | | | | |
| --- | --- | --- | --- | --- | --- | --- | --- | --- | --- | --- | --- | --- |
| **Independent variable (X)** | **Mediation variable (M)** | **Dependent variable (Y)** | **Total effect** | ***p _total_*** | **Effect of X on M** | ***p***  ***_X on M_*** | **Effect of M on Y** | ***p***  ***_M on Y_*** | **Direct effect** | ***p _direct_*** | **Mediation effect [95% CI]** | ***p***  ***_mediation_*** |
| **Valid-PRS_MDD_** | **BL Hopelessness** | **BL Alcohol Use Problems** | 0.004 | 0.014 | 6.63E-05 | 0.439 | 0.34 | 9.11E-05 | 0.004 | 0.014 | 2.26E-05  [-2.29E-04, ∞] | 0.439 |
| **Valid-PRS_MDD_** | **BL Anxiety Sensitivity** | **BL Alcohol Use Problems** | 0.004 | 0.014 | -3.21E-04 | 0.257 | 2.58E-04 | 0.499 | 0.004 | 0.014 | -8.28E-08 [-∞, 7.56E-05] | 0.503 |
| **Valid-PRS_MDD_** | **BL Impulsivity** | **BL Alcohol Use Problems** | 0.004 | 0.014 | 7.64E-04 | 0.054 | 0.619 | 2.54E-14 | 0.003 | 0.026 | 4.73E-04  [-5.09E-05, ∞] | 0.057 |
| **Valid-PRS_MDD_** | **BL Sensation Seeking** | **BL Alcohol Use Problems** | 0.004 | 0.014 | 4E-04 | 0.223 | 0.305 | 2.56E-05 | 0.004 | 0.017 | 1.22E-04  [-1.52E-04, ∞] | 0.233 |
| **Valid-PRS_MDD_** | **FU1 Hopelessness** | **FU1 Alcohol Use Problems** | 0.004 | 0.004 | 9.82E-04 | 0.001 | 0.504 | 2.05E-05 | 0.003 | 0.011 | 5E-04 [1.65E-04, ∞] | **0.0007** |
| **Valid-PRS_MDD_** | **FU1 Anxiety Sensitivity** | **FU1 Alcohol Use Problems** | 0.004 | 0.004 | -5.81E-05 | 0.429 | 0.163 | 0.086 | 0.004 | 0.004 | -9.48E-06 [-∞, 9.89E-05] | 0.426 |
| **Valid-PRS_MDD_** | **FU1 Impulsivity** | **FU1 Alcohol Use Problems** | 0.004 | 0.004 | 7.27E-04 | 0.009 | 0.945 | 1.87E-14 | 0.003 | 0.014 | 6.87E-04 [1.6E-04, ∞] | **0.011** |
| **Valid-PRS_MDD_** | **FU1 Sensation Seeking** | **FU1 Alcohol Use Problems** | 0.004 | 0.004 | -2.5E-04 | 0.239 | 0.769 | 8.25E-13 | 0.004 | 0.003 | -1.92E-04 [-∞, 2.76E-04] | 0.247 |
| **Valid-PRS_MDD_** |  | **FU2 Alcohol Use Problems** | 0.002 | 0.259 |  |  |  |  |  |  |  |  |
| **Valid-PRS_MDD_** | **FU3 Hopelessness** | **FU3 Alcohol Use Problems** | 0.004 | 0.0497 | 0.0012 | 0.002 | 0.747 | 2.27E-06 | 0.003 | 0.103 | 9.2E-04 [2.7E-04, ∞] | **0.0009** |
| **Valid-PRS_MDD_** | **FU3 Anxiety Sensitivity** | **FU3 Alcohol Use Problems** | 0.004 | 0.0497 | 0.0004 | 0.187 | 0.577 | 0.0002 | 0.004 | 0.059 | 2.12E-04  [-2.42E-04, ∞] | 0.197 |
| **Valid-PRS_MDD_** | **FU3 Impulsivity** | **FU3 Alcohol Use Problems** | 0.004 | 0.0497 | 0.0004 | 0.107 | 1.88 | 3.15E-23 | 0.003 | 0.088 | 8.2E-04  [-3.18E-04, ∞] | 0.118 |
| **Valid-PRS_MDD_** | **FU3 Sensation Seeking** | **FU3 Alcohol Use Problems** | 0.004 | 0.0497 | -0.0002 | 0.318 | 0.885 | 2.94E-09 | 0.004 | 0.040 | -1.87E-04 [-∞, 5.12E-04] | 0.322 |
| **Valid-PRS_alcohol_** |  | **BL Depression Scores** | 436 | 0.356 |  |  |  |  |  |  |  |  |
| **Valid-PRS_alcohol_** |  | **FU1 Depression Scores** | -2081 | 0.061 |  |  |  |  |  |  |  |  |
| **Valid-PRS_alcohol_** |  | **FU2 Depression Scores** | 2.05 | 0.499 |  |  |  |  |  |  |  |  |
| **Valid-PRS_alcohol_** | **FU3 ANXDX Scores** | **FU3 Depression Scores** | -2358 | 0.021 | -10851 | 0.014 | 0.135 | 1.35E-100 | -894 | 0.174 | -1463  [-∞, -413] | **0.011** |
| **Valid-PRS_alcohol_** | **FU3 Neuroticism** | **FU3 Depression Scores** | -2756 | 0.016 | -926 | 0.035 | 1.55 | 2.3E-99 | -1320 | 0.095 | -1435  [-∞, -103] | **0.036** |

| **Males** | | | | | | | | | | | | |
| --- | --- | --- | --- | --- | --- | --- | --- | --- | --- | --- | --- | --- |
| **Independent variable (X)** | **Mediation variable (M)** | **Dependent variable (Y)** | **Total effect** | ***p _total_*** | **Effect of X on M** | ***p***  ***_X on M_*** | **Effect of M on Y** | ***p***  ***_M on Y_*** | **Direct effect** | ***p _direct_*** | **Mediation effect [95% CI]** | ***p***  ***_mediation_*** |
| **Valid-PRS_MDD_** | **BL Hopelessness** | **BL Alcohol Use Problems** | 0.005 | 0.033 | 7.45E-04 | 0.105 | 0.269 | 0.029 | 0.005 | 0.039 | 2E-04  [-2.38E-04, ∞] | 0.164 |
| **Valid-PRS_MDD_** | **BL Anxiety Sensitivity** | **BL Alcohol Use Problems** | 0.005 | 0.033 | 8.52E-04 | 0.109 | -0.011 | 4.63E-01 | 0.005 | 0.033 | -9.56E-06 [-∞, 2.13E-04] | 0.480 |
| **Valid-PRS_MDD_** | **BL Impulsivity** | **BL Alcohol Use Problems** | 0.005 | 0.033 | 0.001 | 0.014 | 0.548 | 5.56E-06 | 0.004 | 0.062 | 8.06E-04 [7.15E-05, ∞] | **0.016** |
| **Valid-PRS_MDD_** | **BL Sensation Seeking** | **BL Alcohol Use Problems** | 0.005 | 0.033 | -3.13E-04 | 0.332 | 0.304 | 4.52E-03 | 0.005 | 0.030 | -9.51E-05 [-∞, 2.93E-04] | 0.333 |
| **Valid-PRS_MDD_** | **FU1 Hopelessness** | **FU1 Alcohol Use Problems** | 0.005 | 0.006 | 6.10E-04 | 0.084 | 0.371 | 0.019 | 0.005 | 0.007 | 2.26E-04 [-1.05E-04, ∞] | 0.094 |
| **Valid-PRS_MDD_** | **FU1 Anxiety Sensitivity** | **FU1 Alcohol Use Problems** | 0.005 | 0.006 | -6.11E-05 | 0.446 | 0.033 | 0.425 | 0.005 | 0.006 | -2.03E-06 [-∞, 1.41E-04] | 0.451 |
| **Valid-PRS_MDD_** | **FU1 Impulsivity** | **FU1 Alcohol Use Problems** | 0.005 | 0.006 | 7.39E-04 | 0.048 | 0.830 | 1.55E-06 | 0.005 | 0.011 | 6.13E-04 [-6.15E-05, ∞] | 0.055 |
| **Valid-PRS_MDD_** | **FU1 Sensation Seeking** | **FU1 Alcohol Use Problems** | 0.005 | 0.006 | -5.32E-04 | 0.137 | 0.866 | 4.04E-08 | 0.006 | 0.003 | -4.61E-04 [-∞, 2.68E-04] | 0.140 |
| **Valid-PRS_MDD_** |  | **FU2 Alcohol Use Problems** | 0.008 | 0.067 |  |  |  |  |  |  |  |  |
| **Valid-PRS_MDD_** |  | **FU3 Alcohol Use Problems** | 0.001 | 0.380 |  |  |  |  |  |  |  |  |
| **Valid-PRS_alcohol_** |  | **BL Depression Scores** | 1044 | 0.239 |  |  |  |  |  |  |  |  |
| **Valid-PRS_alcohol_** |  | **FU1 Depression Scores** | -4349 | 0.005 | -3159 | 0.308 | 0.087 | 2.5E-14 | -4073 | 0.005 | -276  [-∞, 773] | 0.315 |
| **Valid-PRS_alcohol_** |  | **FU2 Depression Scores** | 303 | 0.414 |  |  |  |  |  |  |  |  |
| **Valid-PRS_alcohol_** |  | **FU3 Depression Scores** | 234 | 0.440 |  |  |  |  |  |  |  |  |

| **Females** | | | | | | | | | | | | |
| --- | --- | --- | --- | --- | --- | --- | --- | --- | --- | --- | --- | --- |
| **Independent variable (X)** | **Mediation variable (M)** | **Dependent variable (Y)** | **Total effect** | ***p _total_*** | **Effect of X on M** | ***p***  ***_X on M_*** | **Effect of M on Y** | ***p***  ***_M on Y_*** | **Direct effect** | ***p _direct_*** | **Mediation effect [95% CI]** | ***p***  ***_mediation_*** |
| **Valid-PRS_MDD_** |  | **BL Alcohol Use Problems** | 0.002 | 0.171 |  |  |  |  |  |  |  |  |
| **Valid-PRS_MDD_** |  | **FU1 Alcohol Use Problems** | 0.002 | 0.157 |  |  |  |  |  |  |  |  |
| **Valid-PRS_MDD_** |  | **FU2 Alcohol Use Problems** | -0.004 | 0.237 |  |  |  |  |  |  |  |  |
| **Valid-PRS_MDD_** | **FU3 Hopelessness** | **FU3 Alcohol Use Problems** | 0.006 | 0.017 | 0.001 | 0.006 | 0.908 | 2.2E-06 | 0.005 | 0.045 | 0.0013 [2.6E-04, ∞] | **0.0035** |
| **Valid-PRS_MDD_** | **FU3 Anxiety Sensitivity** | **FU3 Alcohol Use Problems** | 0.006 | 0.017 | 3.5E-04 | 0.269 | 0.495 | 0.006 | 0.006 | 0.019 | 1.7E-04  [-3.3E-04, ∞] | 0.27 |
| **Valid-PRS_MDD_** | **FU3 Impulsivity** | **FU3 Alcohol Use Problems** | 0.006 | 0.017 | 3.7E-04 | 0.218 | 1.64 | 1E-12 | 0.005 | 0.023 | 6E-4  [-6.6E-04, ∞] | 0.205 |
| **Valid-PRS_MDD_** | **FU3 Sensation Seeking** | **FU3 Alcohol Use Problems** | 0.006 | 0.017 | -3E-04 | 0.320 | 0.86 | 4.2E-07 | 0.006 | 0.012 | -2.6E-04  [-∞, 7.2E-04] | 0.326 |
| **Valid-PRS_alcohol_** |  | **BL Depression Scores** | -157 | 0.465 |  |  |  |  |  |  |  |  |
| **Valid-PRS_alcohol_** |  | **FU1 Depression Scores** | -318 | 0.439 |  |  |  |  |  |  |  |  |
| **Valid-PRS_alcohol_** |  | **FU2 Depression Scores** | -528 | 0.387 |  |  |  |  |  |  |  |  |
| **Valid-PRS_alcohol_** | **FU3 ANXDX Scores** | **FU3 Depression Scores** | -4427 | 0.005 | -20249 | 0.002 | 0.15 | 2.8E-63 | -1404 | 0.151 | -3023  [-∞, -1237] | **0.0015** |
| **Valid-PRS_alcohol_** | **FU3 Neuroticism** | **FU3 Depression Scores** | -4756 | 0.005 | -1432 | 0.018 | 1.75 | 7.1E-62 | -2256 | 0.056 | -2501  [-∞, -477] | **0.02** |

**Table S17. Top SNPs (n = 10) for Alcohol Consumption GWAS**

| **SNP** | **Effect allele** | **Other allele** | **BETA** | **SE** | **P value** |
| --- | --- | --- | --- | --- | --- |
| rs780094 | T | C | -0.0155 | 0.0026 | 3.65E-09 |
| rs1260326 | T | C | -0.0148 | 0.0026 | 1.32E-08 |
| rs780093 | T | C | -0.014 | 0.0026 | 5.07E-08 |
| rs197273 | A | G | -0.0141 | 0.0026 | 9.80E-08 |
| rs10950202 | C | G | 0.0194 | 0.0038 | 2.90E-07 |
| rs13222337 | T | C | -0.0188 | 0.0037 | 2.94E-07 |
| rs11940694 | A | G | -0.0137 | 0.0027 | 3.20E-07 |
| rs350721 | C | G | 0.0206 | 0.004 | 3.23E-07 |
| rs350732 | C | G | 0.0205 | 0.004 | 3.60E-07 |
| rs2603388 | T | C | -0.0205 | 0.0041 | 4.33E-07 |

**Top SNPs (n = 10) for MDD GWAS**

| **SNP** | **effect allele** | **other allele** | **OR** | **SE** | ***p* value** |
| --- | --- | --- | --- | --- | --- |
| rs1936365 | C | G | 0.92886 | 0.0112 | 3.86E-11 |
| rs853676 | T | C | 0.92941 | 0.0112 | 5.13E-11 |
| rs853685 | T | C | 0.9296 | 0.0112 | 6.16E-11 |
| rs853678 | A | T | 0.92979 | 0.0112 | 6.89E-11 |
| rs853679 | A | C | 0.92988 | 0.0112 | 7.43E-11 |
| rs853681 | A | C | 0.93016 | 0.0112 | 8.62E-11 |
| rs1592757 | C | G | 1.05422 | 0.0083 | 1.90E-10 |
| rs10479296 | A | G | 0.95237 | 0.0079 | 6.81E-10 |
| rs10053368 | A | G | 1.04991 | 0.0079 | 7.77E-10 |
| rs396755 | C | G | 1.05064 | 0.008 | 8.01E-10 |

**Table S18. The correlation between the GSCAN-based Polygenic Risk Score and alcohol consumption in the IMAGEN cohort**

| **The correlation between the GSCAN-based PRS and Alcohol Consumption** | | |
| --- | --- | --- |
| **Time** | ***r*** | ***p*** |
| **Baseline** | 0.0116 | 0.6116 |
| **Follow-up 1** | -0.0299 | 0.2426 |
| **Follow-up 2** | 0.0102 | 0.7081 |
| **Follow-up 3** | 0.0404 | 0.1616 |


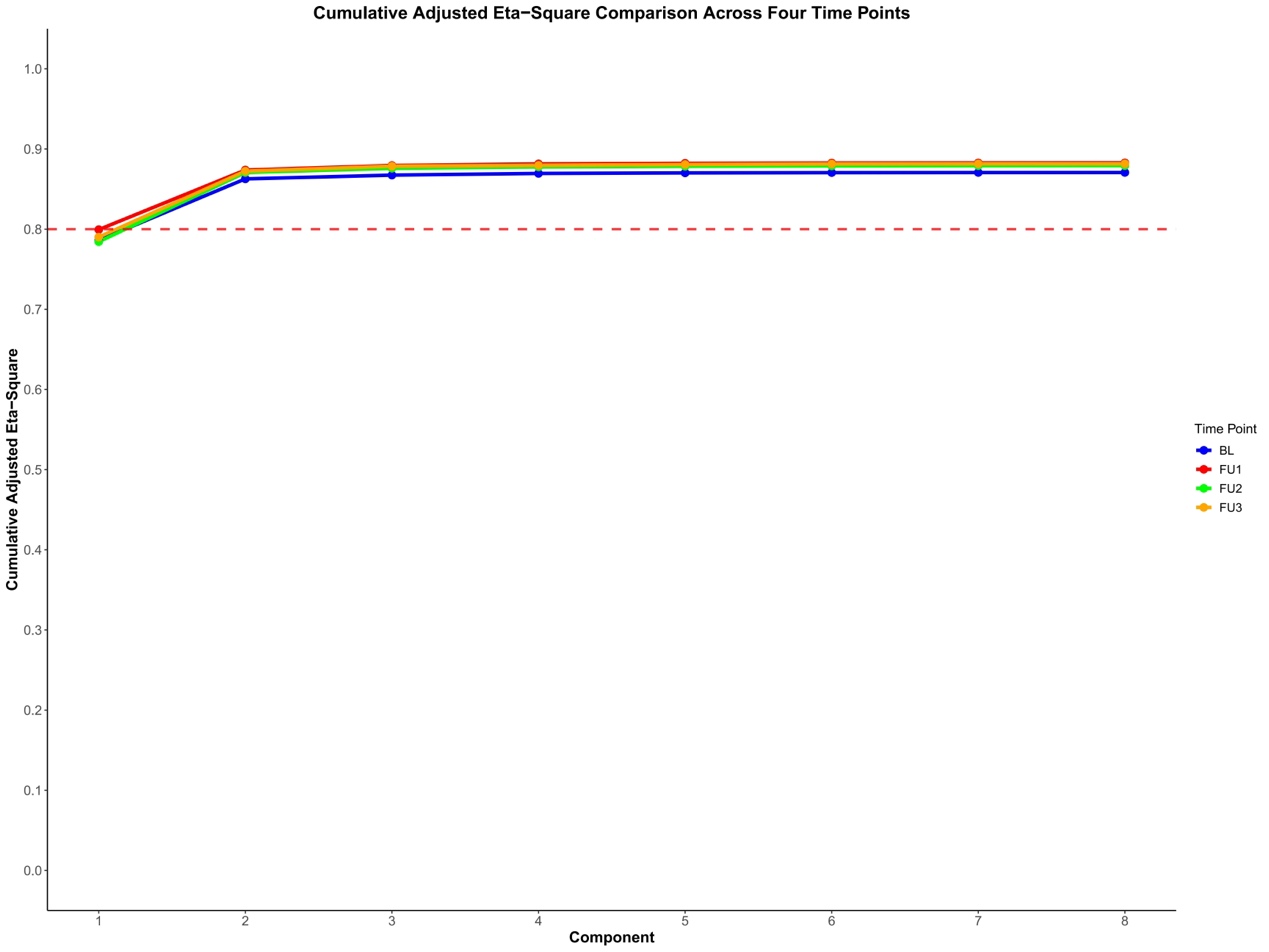


**Figure S1. The cumulative adjusted Eta-square between Sites/Sex and the Top 10 genetic principal components in the IMAGEN cohort**


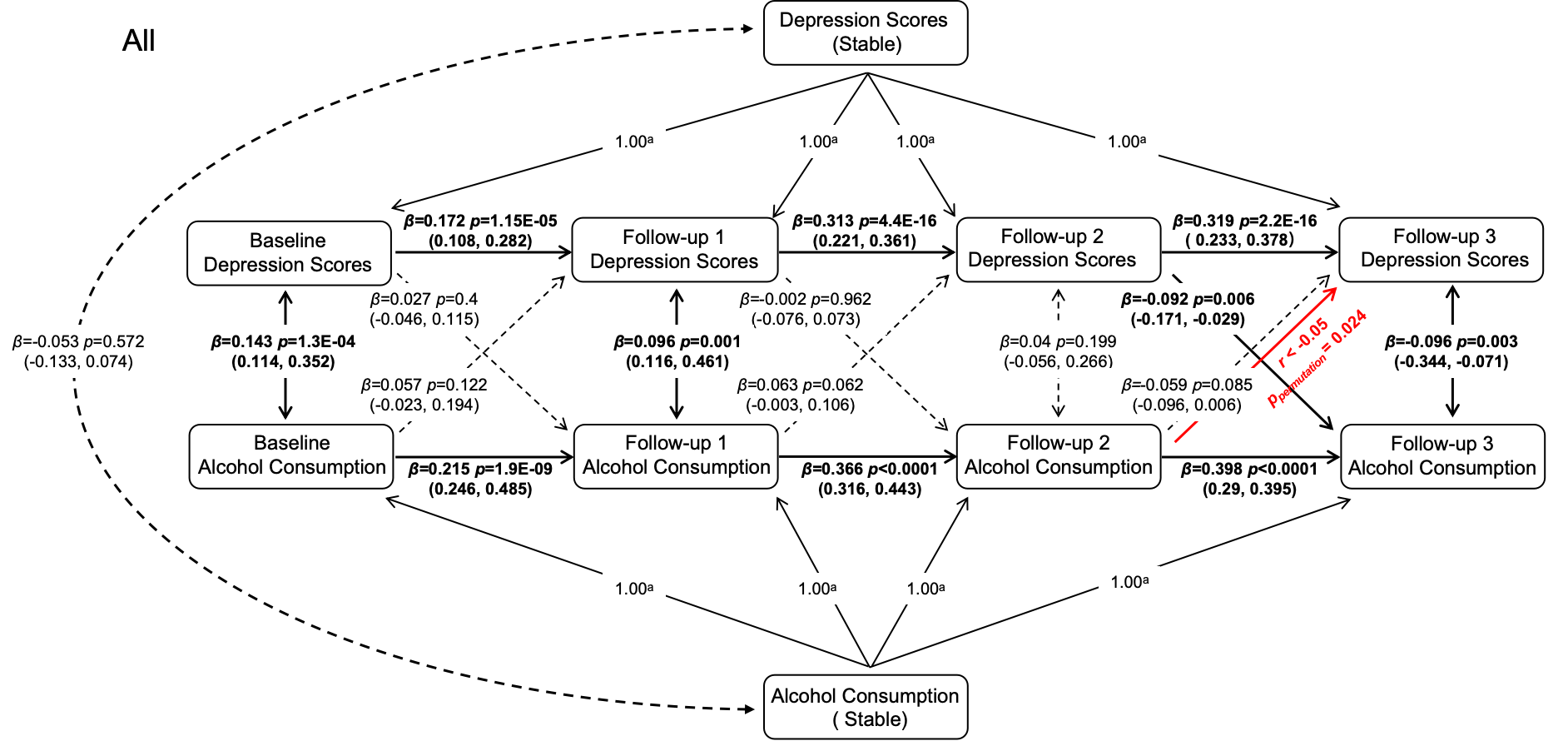


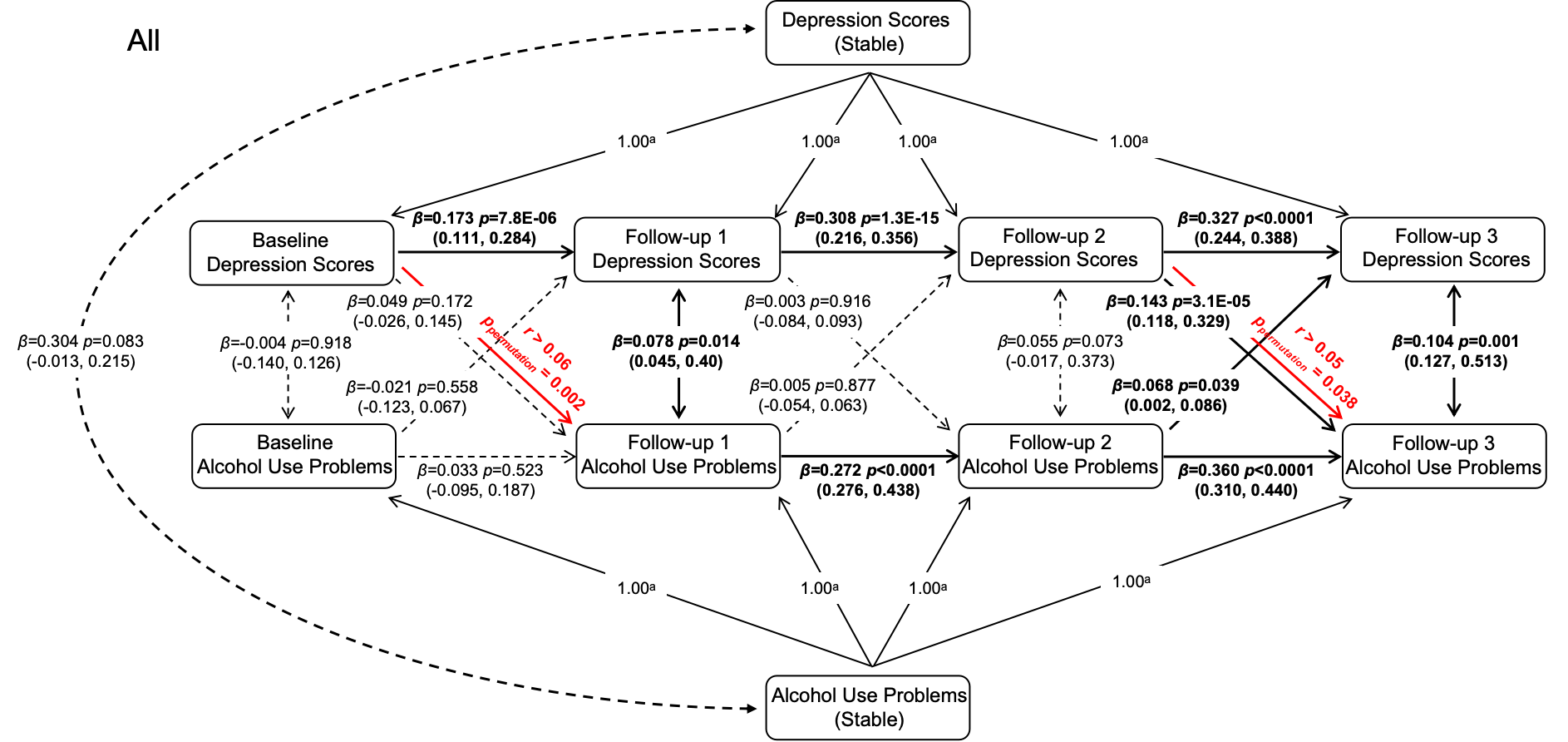


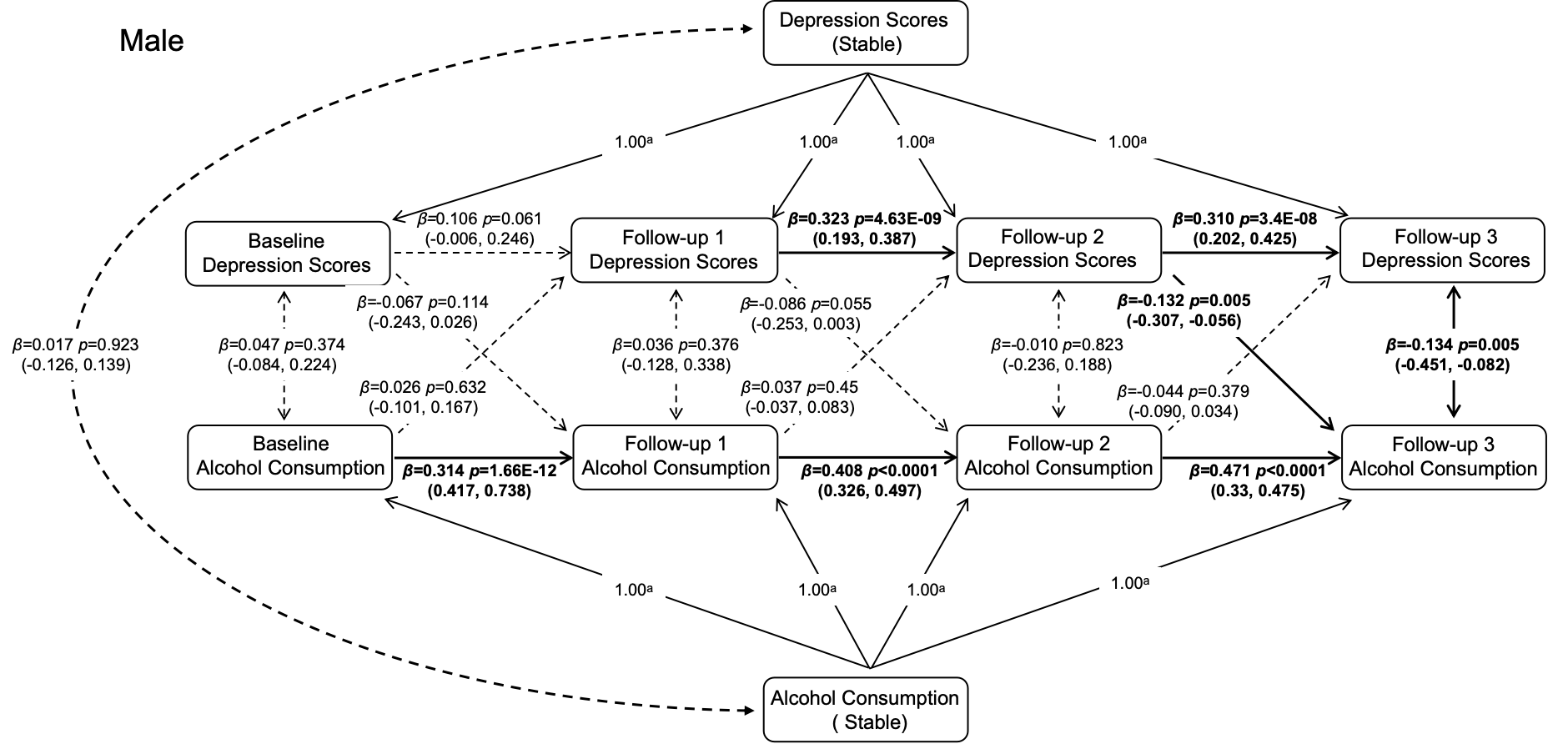


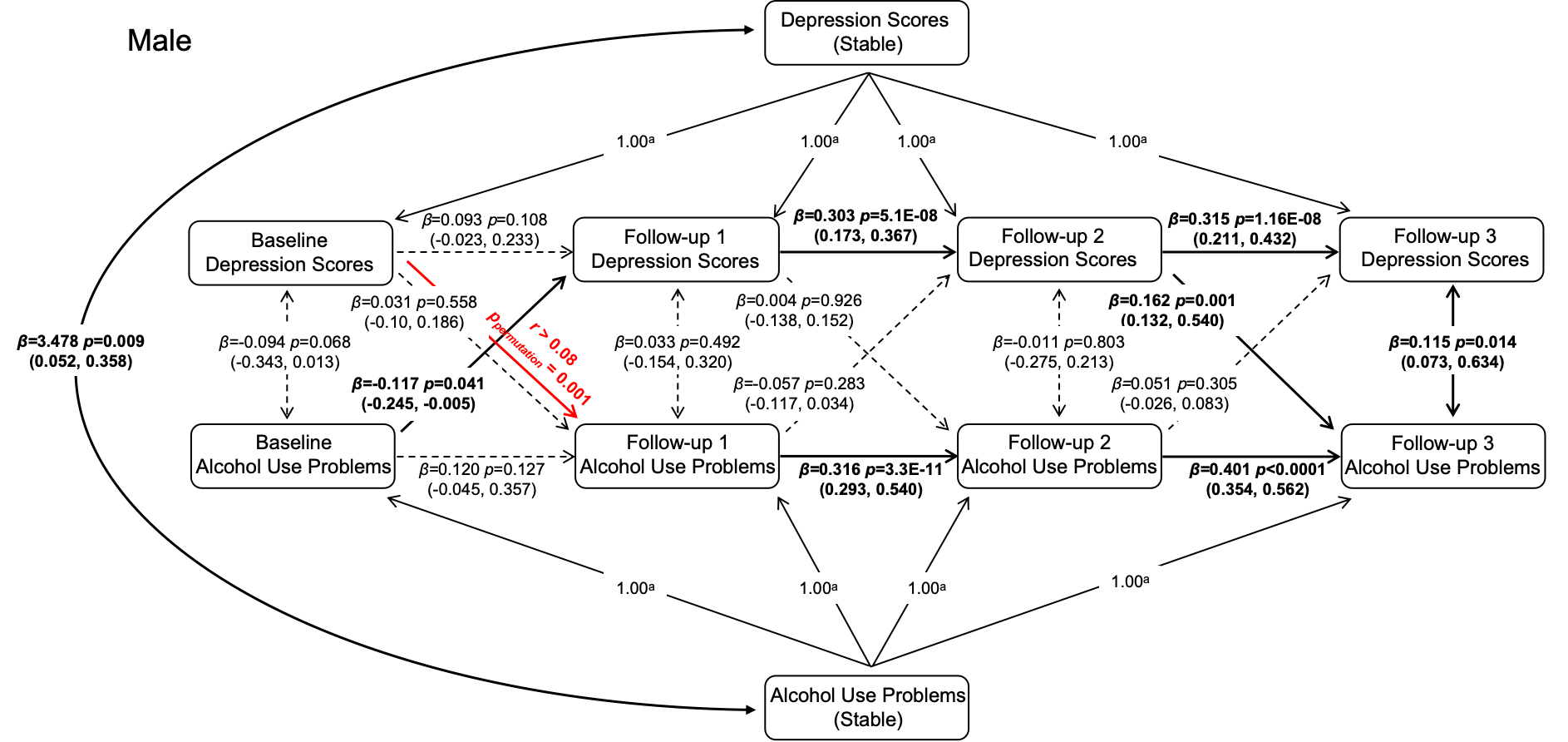


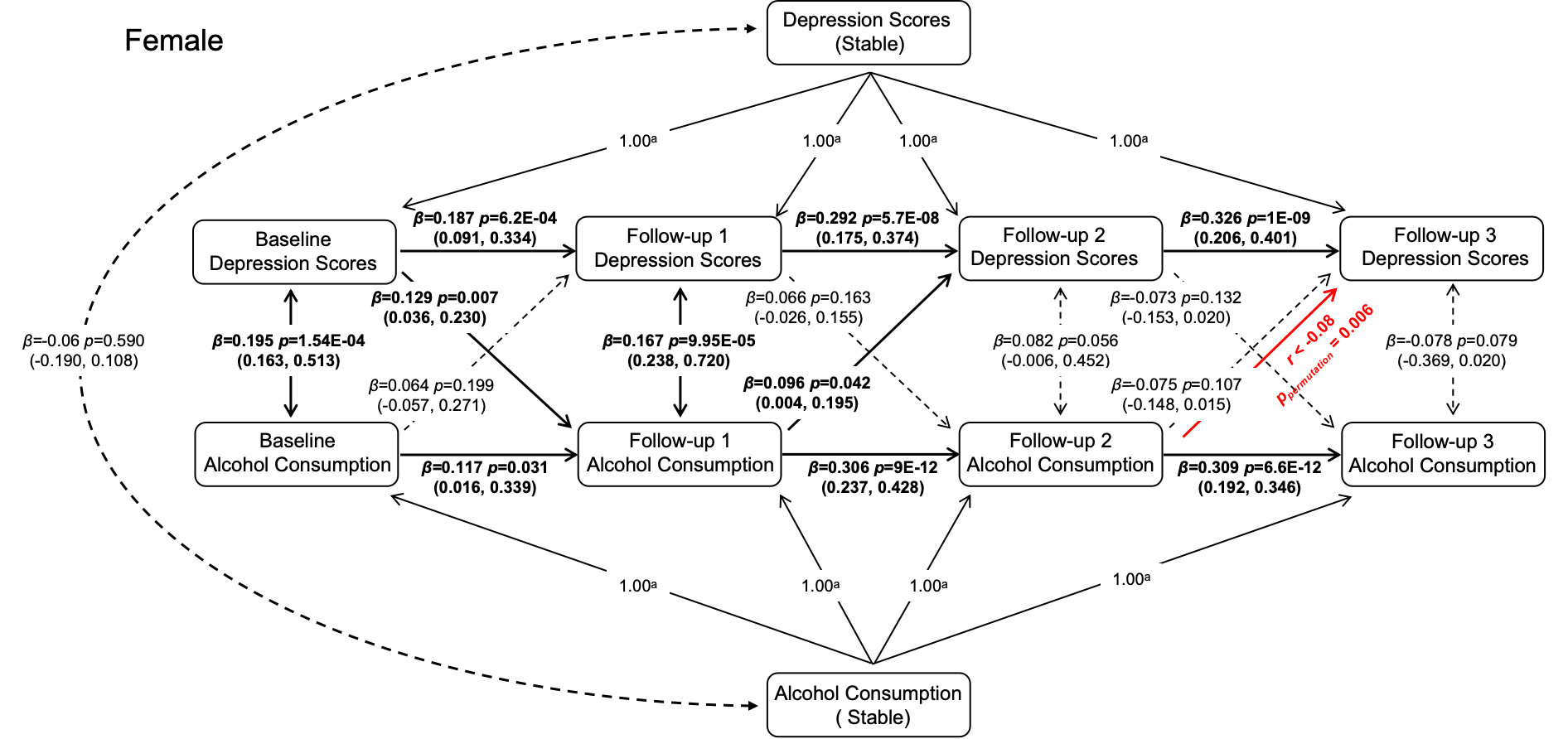


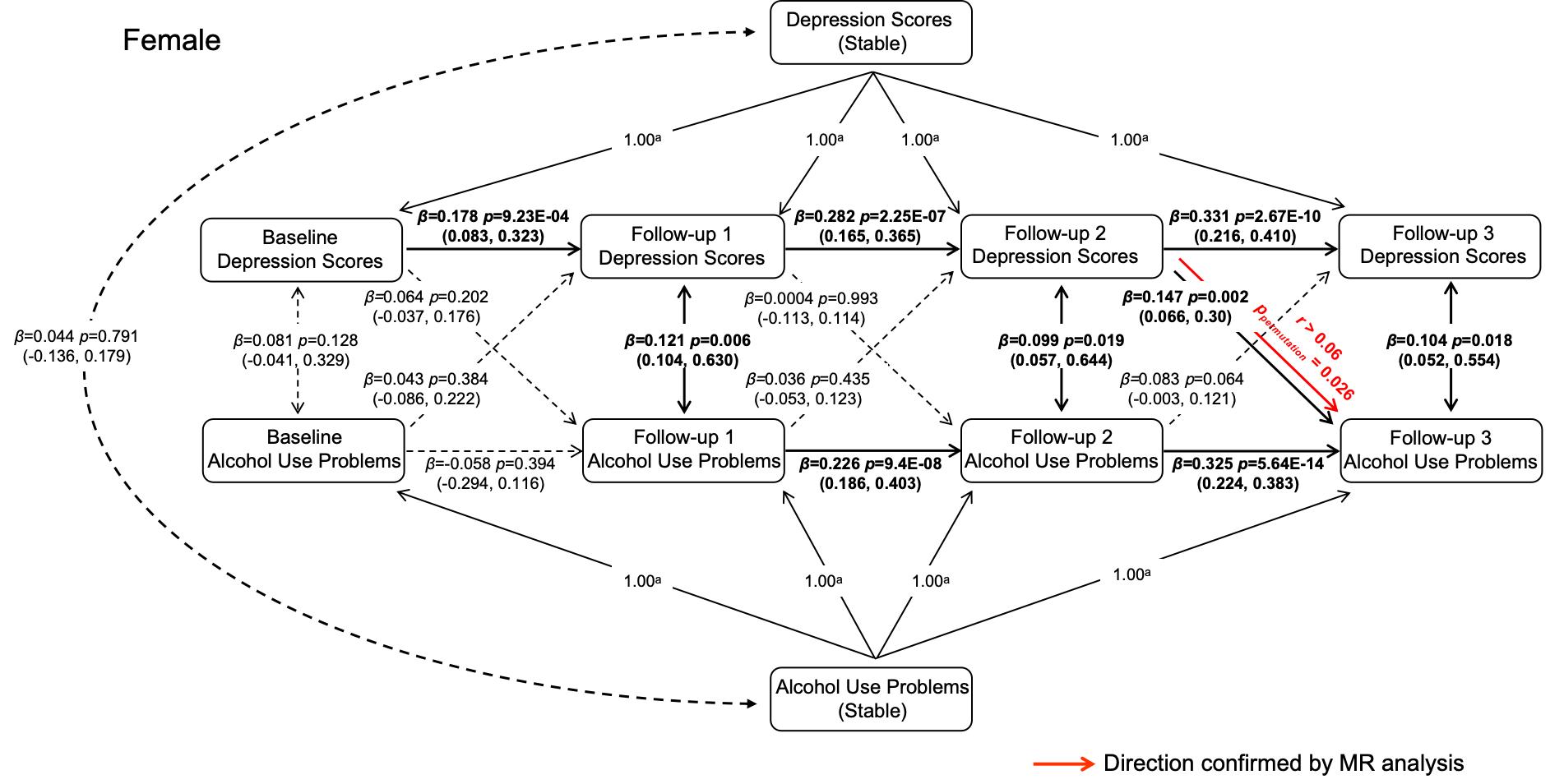


**Figure S2. Random-intercepts cross-lagged panel model of depression scores and alcohol consumption/alcohol use problems from ages 14 to 23 years in the IMAGEN cohort, adjusted for the other alcohol use behaviour (n = 2093)**. Standardized estimates are presented with 95% confidence intervals. Solid lines represent significant associations (two-tailed *p* < .05), while dashed lines represent nonsignificant associations (two-tailed *p* > .05). Model fit: root mean square error of approximation < .045; comparative fit index > .975; standardized root mean square residual < .032. ᵃ Pathways were constrained to 1.00 to isolate the between-person factor. **Causal inferences (highlighted in red) were confirmed with improved Mendelian randomization (MR).** *r* is the correlation between the valid-PRS and the corresponding behaviour, and *p_permutation_* is the value of the permutation test (see Table S11).


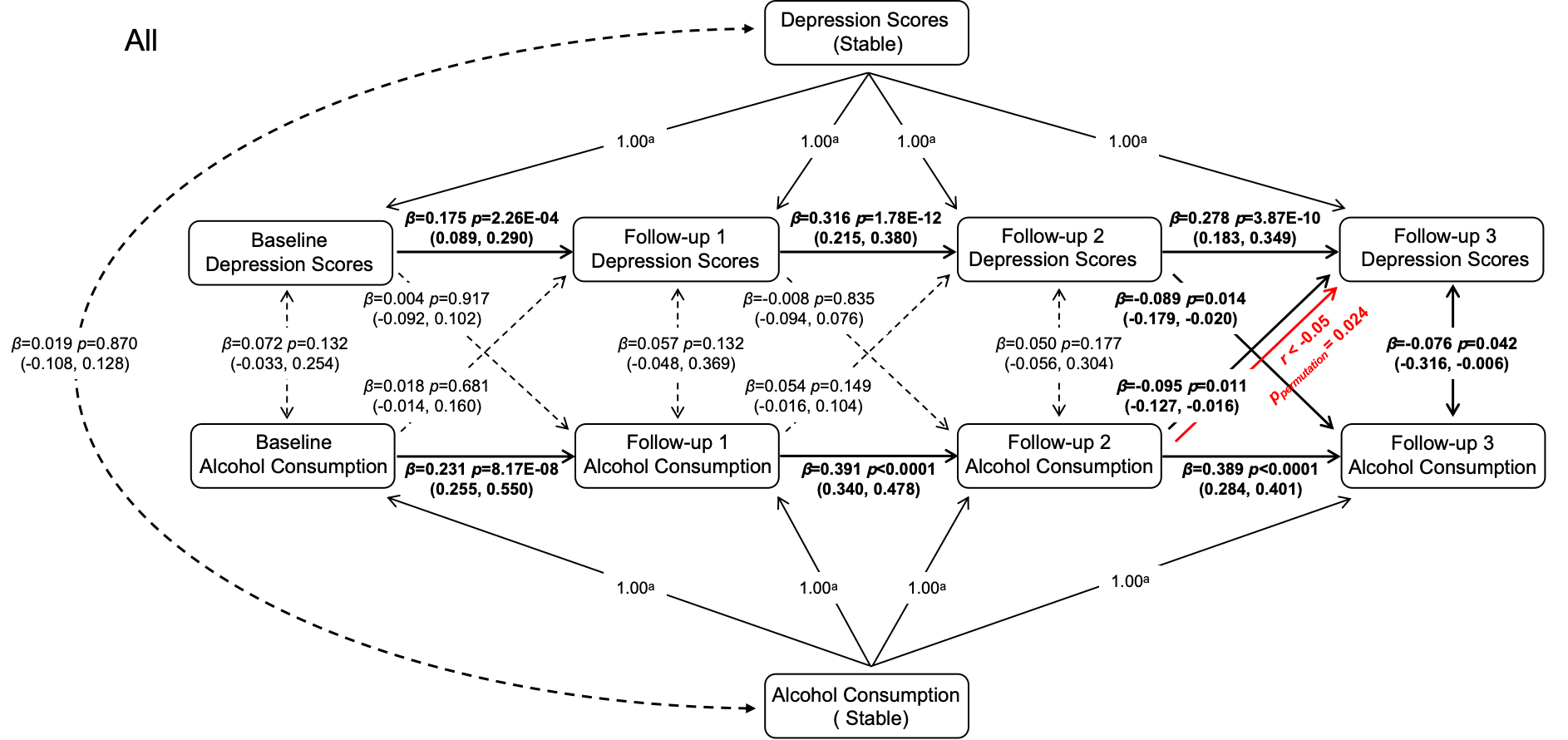


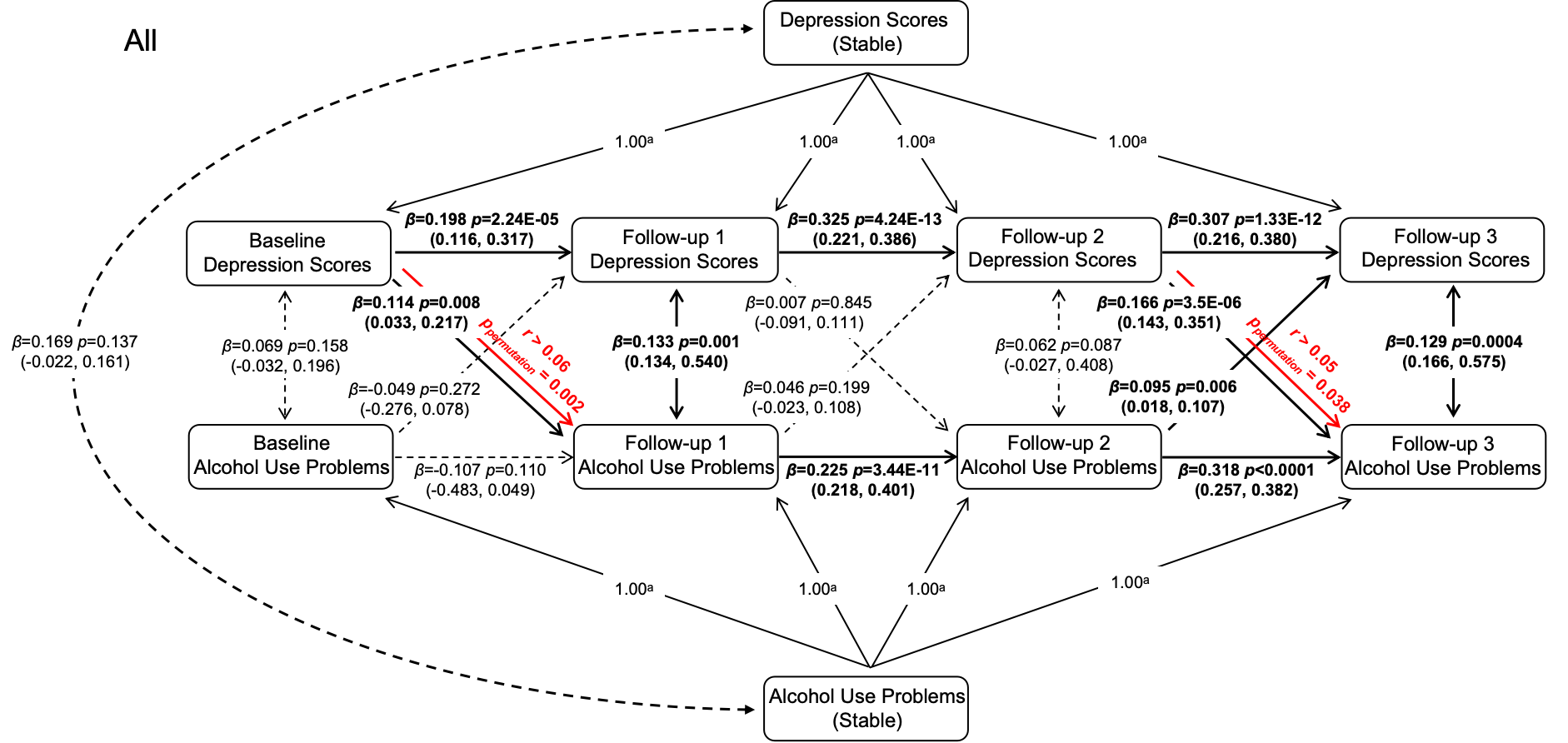


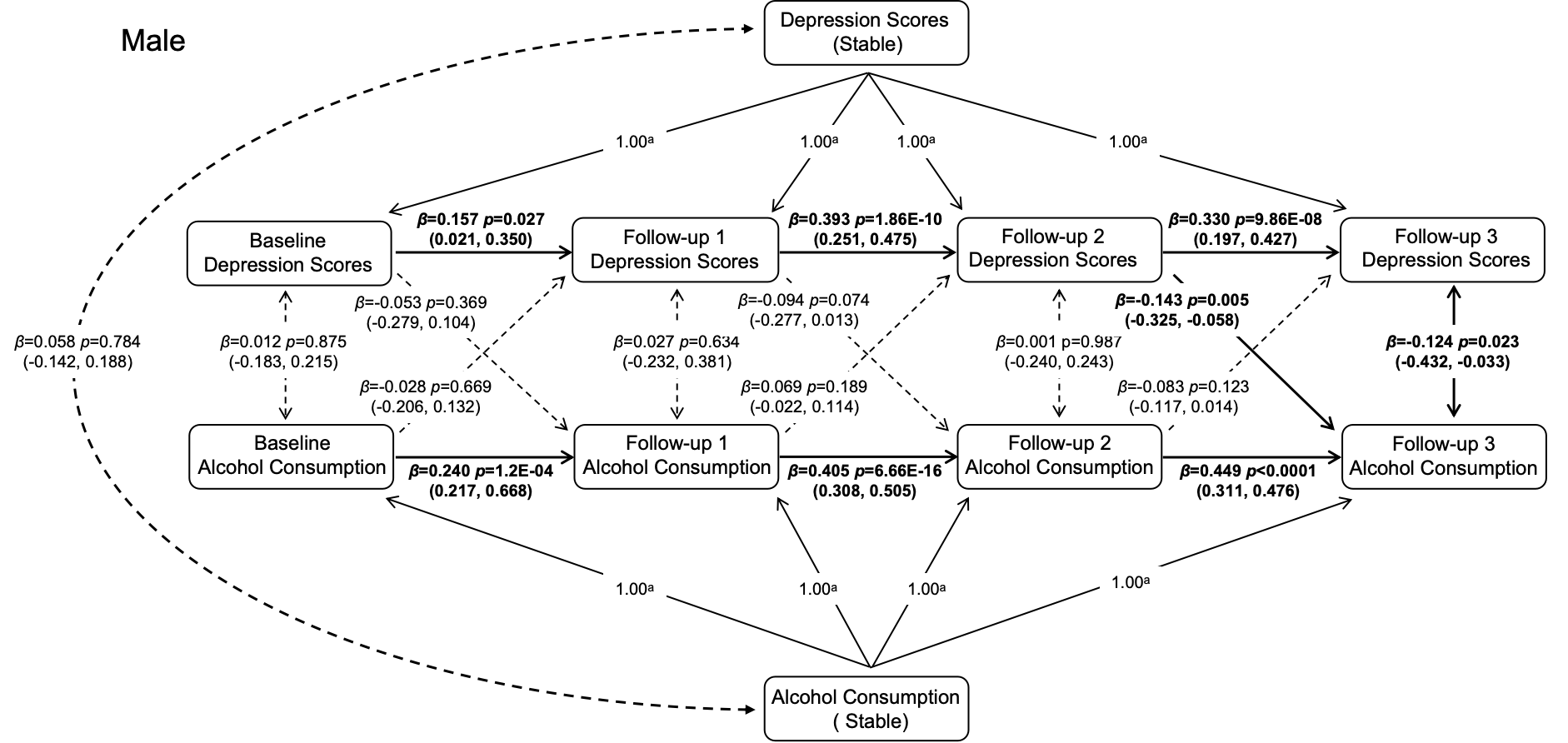


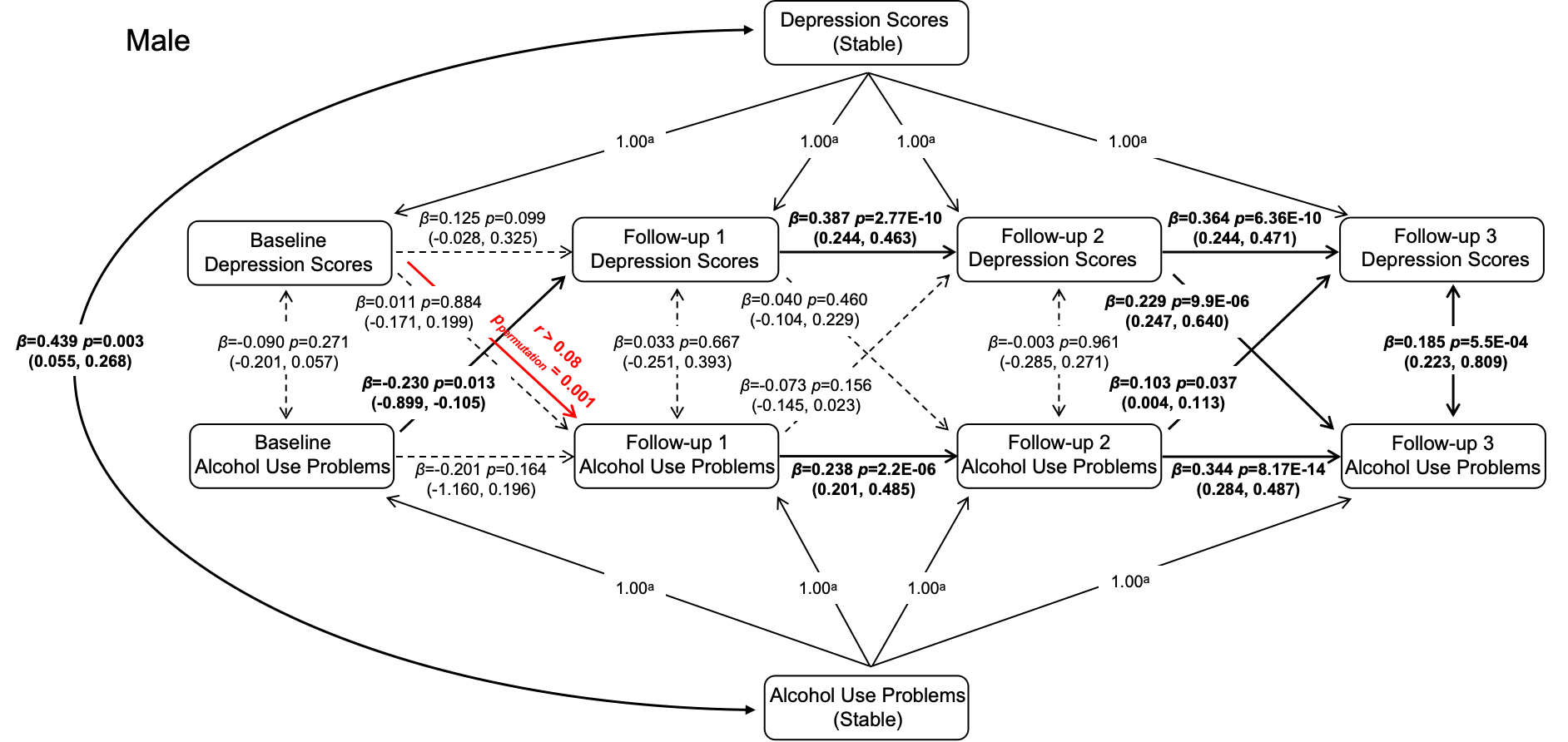


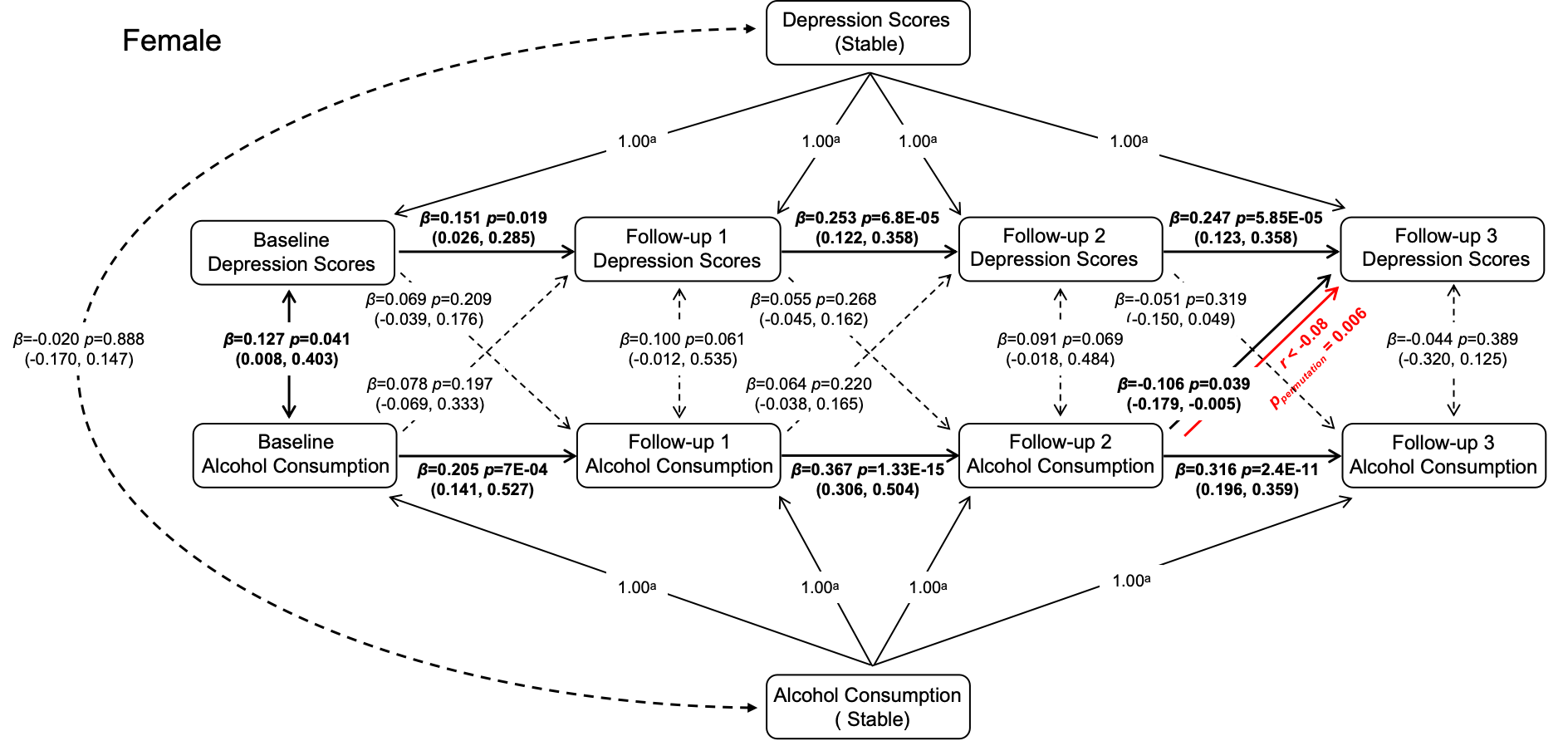


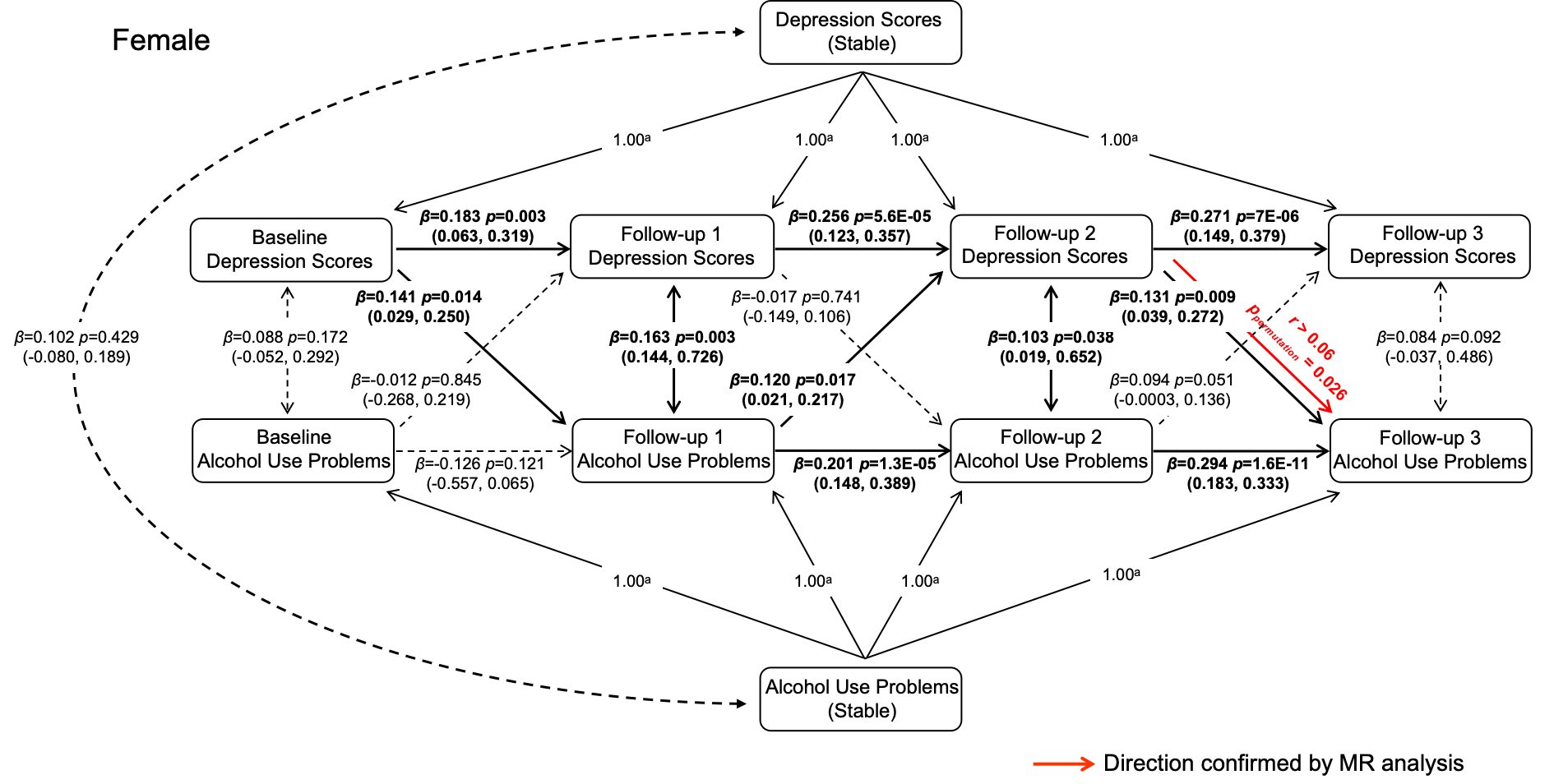


**Figure S3. Random-intercepts cross-lagged panel model of depression scores and alcohol consumption/alcohol use problems from ages 14 to 23 years in the IMAGEN cohort (controlled for the other alcohol use behaviour) (n = 932 with complete longitudinal data at all four time points)**. Standardized estimates are presented with 95% confidence intervals. Solid lines represent significant associations (two-tailed *p* < .05), whereas dashed lines represent nonsignificant associations (two-tailed *p* > .05). Model fit: root mean square error of approximation < .055; comparative fit index > .977; standardized root mean square residual < .031. ᵃ Pathways constrained to 1.00 to isolate between-person factor. **Causal inferences (highlighted in red) were confirmed with improved Mendelian randomization (MR).** *r* is the correlation between the valid-PRS and the corresponding behaviour, and *p_permutation_* is the value of the permutation test (see Table S11).


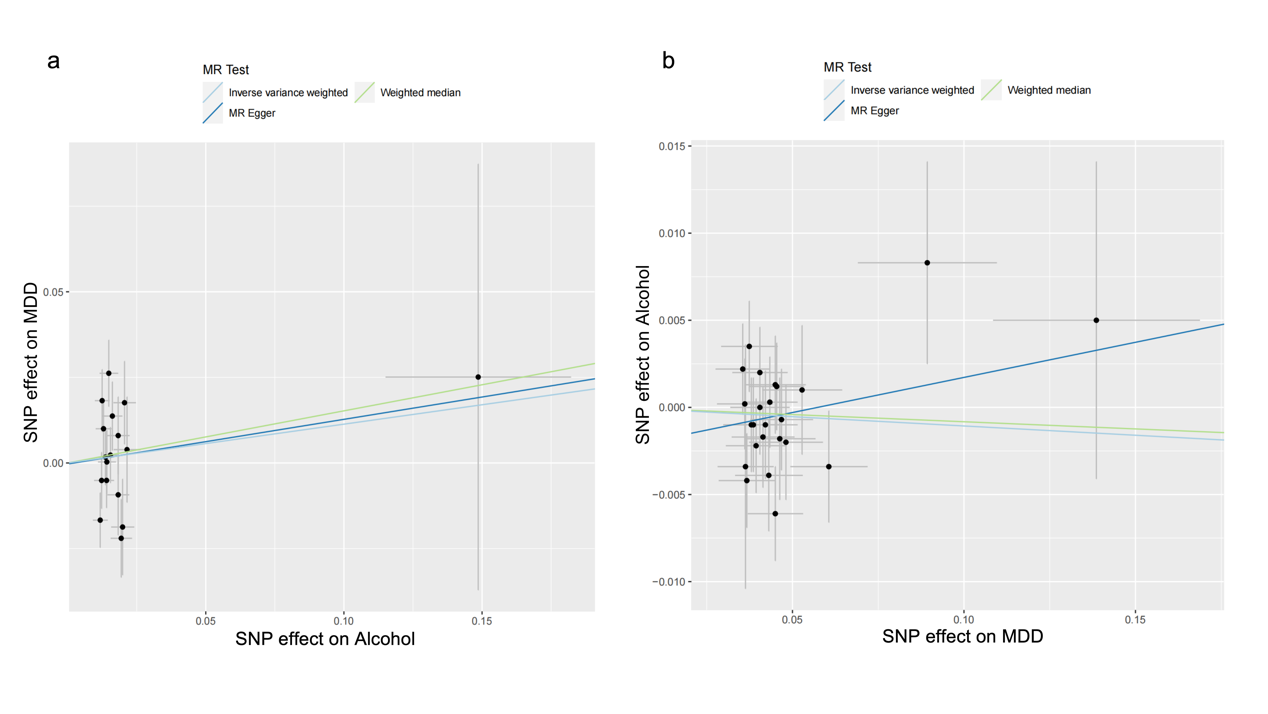


**Figure S4. Results of two-sample Mendelian randomisation analyses.** (a). Each point represents the effect estimate. of each SNP and its standard error. The horizontal axis represents the effect estimates of SNP on Alcohol. The vertical axis represents the effect estimates of SNP on MDD. The slope of the regression line is an estimate of the causal effect of Alcohol on MDD. Using the IVW meta-analysis (Davies, Holmes, & Davey Smith, 2018) of MR based on SNP genetic instruments, we found a not significant causal role of Alcohol over MDD (*β* = 0.11, *se* = 0.20, *p* = 0.56). The same conclusion was obtained using the weighted median analysis (Bowden, Davey Smith, Haycock, & Burgess, 2016) (*β* = 0.15, *se* = 0.22, *p* = 0.48) and the MR–Egger regression (Bowden, Davey Smith, & Burgess, 2015) (*β* = 0.13, *se* = 0.56, *p* = 0.82). (b). Each point represents the effect estimate of each SNP and its standard error. The horizontal axis represents the effect estimates of SNP on MDD. The vertical axis represents the effect estimates of SNP on Alcohol. The slope of the regression line is an estimate of the causal effect of MDD on Alcohol. Using the IVW meta-analysis of MR based on SNP genetic instruments, we found a not significant causal role of MDD over Alcohol (*β* = -0.011, *se* = 0.014, *p* = 0.44). The same conclusion was obtained using the weighted median analysis (*β* = -0.0082, *se* = 0.019, *p* = 0.67). The contrary conclusion was obtained using the MR–Egger regression (*β* = 0.04, *se* = 0.063, *p* = 0.53).

**Reference**

Allen, J. P., Litten, R. Z., Fertig, J. B., & Babor, T. (1997). A review of research on the Alcohol Use Disorders Identification Test (AUDIT). *Alcohol Clin Exp Res, 21*(4), 613-619.

Bowden, J., Davey Smith, G., & Burgess, S. (2015). Mendelian randomization with invalid instruments: effect estimation and bias detection through Egger regression. *Int J Epidemiol, 44*(2), 512-525. doi:10.1093/ije/dyv080

Bowden, J., Davey Smith, G., Haycock, P. C., & Burgess, S. (2016). Consistent Estimation in Mendelian Randomization with Some Invalid Instruments Using a Weighted Median Estimator. *Genet Epidemiol, 40*(4), 304-314. doi:10.1002/gepi.21965

Cook, R. L., Chung, T., Kelly, T. M., & Clark, D. B. (2005). Alcohol screening in young persons attending a sexually transmitted disease clinic. Comparison of AUDIT, CRAFFT, and CAGE instruments. *J Gen Intern Med, 20*(1), 1-6. doi:10.1111/j.1525-1497.2005.40052.x

Costa, P. T., & McCrae, R. R. (1992). Normal personality assessment in clinical practice: The NEO Personality Inventory. *Psychological Assessment, 4*(1), 5-13. doi:10.1037/1040-3590.4.1.5

Davies, N. M., Holmes, M. V., & Davey Smith, G. (2018). Reading Mendelian randomisation studies: a guide, glossary, and checklist for clinicians. *Bmj, 362*, k601. doi:10.1136/bmj.k601

Goodman, R. (2001). Psychometric properties of the strengths and difficulties questionnaire. *J Am Acad Child Adolesc Psychiatry, 40*(11), 1337-1345. doi:10.1097/00004583-200111000-00015

Goodman, R., Ford, T., Richards, H., Gatward, R., & Meltzer, H. (2000). The Development and Well-Being Assessment: description and initial validation of an integrated assessment of child and adolescent psychopathology. *J Child Psychol Psychiatry, 41*(5), 645-655.

Knappe, S., Klotsche, J., Heyde, F., Hiob, S., Siegert, J., Hoyer, J., . . . Beesdo-Baum, K. (2014). Test-retest reliability and sensitivity to change of the dimensional anxiety scales for DSM-5. *CNS Spectr, 19*(3), 256-267. doi:10.1017/s1092852913000710

Lebeau, R. T., Glenn, D. E., Hanover, L. N., Beesdo-Baum, K., Wittchen, H. U., & Craske, M. G. (2012). A dimensional approach to measuring anxiety for DSM-5. *Int J Methods Psychiatr Res, 21*(4), 258-272. doi:10.1002/mpr.1369

Vulser, H., Lemaitre, H., Artiges, E., Miranda, R., Penttilä, J., Struve, M., . . . Paillère-Martinot, M. L. (2015). Subthreshold depression and regional brain volumes in young community adolescents. *J Am Acad Child Adolesc Psychiatry, 54*(10), 832-840. doi:10.1016/j.jaac.2015.07.006

Woicik, P. A., Stewart, S. H., Pihl, R. O., & Conrod, P. J. (2009). The Substance Use Risk Profile Scale: a scale measuring traits linked to reinforcement-specific substance use profiles. *Addict Behav, 34*(12), 1042-1055. doi:10.1016/j.addbeh.2009.07.001

Xie, C., Jia, T., Rolls, E. T., Robbins, T. W., Sahakian, B. J., Zhang, J., . . . Feng, J. (2021). Reward Versus Nonreward Sensitivity of the Medial Versus Lateral Orbitofrontal Cortex Relates to the Severity of Depressive Symptoms. *Biol Psychiatry Cogn Neurosci Neuroimaging, 6*(3), 259-269. doi:10.1016/j.bpsc.2020.08.017
